# Bacterial community and arsenic functional genes diversity in arsenic contaminated soils from different geographic locations

**DOI:** 10.1371/journal.pone.0176696

**Published:** 2017-05-05

**Authors:** Yunfu Gu, Joy D. Van Nostrand, Liyou Wu, Zhili He, Yujia Qin, Fang-Jie Zhao, Jizhong Zhou

**Affiliations:** 1 Department of Microbiology, College of Resources Sciences and Technology, Sichuan Agricultural University, Chengdu, China; 2 Department of Microbiology and Plant Biology, Institute for Environmental Genomics, University of Oklahoma, Norman, Oklahoma, United States of America; 3 College of Resources and Environmental Sciences, Nanjing Agricultural University, Nanjing, China; 4 Sustainable Soil and Grassland Systems Department, Rothamsted Research, Harpenden, Hertfordshire, United Kingdom; 5 State Key Joint Laboratory of Environment Simulation and Pollution Control, School of Environment, Tsinghua University, Beijing, China; 6 Earth Sciences Division, Lawrence Berkeley National Laboratory, Berkeley, California, United States of America; Wageningen University, NETHERLANDS

## Abstract

To understand how soil microbial communities and arsenic (As) functional genes respond to soil arsenic (As) contamination, five soils contaminated with As at different levels were collected from diverse geographic locations, incubated for 54 days under flooded conditions, and examined by both MiSeq sequencing of 16S rRNA gene amplicons and functional gene microarray (GeoChip 4.0). The results showed that both bacterial community structure and As functional gene structure differed among geographical locations. The diversity of As functional genes correlated positively with the diversity of 16S rRNA genes (P< 0.05). Higher diversities of As functional genes and 16S rRNA genes were observed in the soils with higher available As. Soil pH, phosphate-extractable As, and amorphous Fe content were the most important factors in shaping the bacterial community structure and As transformation functional genes. Geographic location was also important in controlling both the bacterial community and As transformation functional potential. These findings provide insights into the variation of As transformation functional genes in soils contaminated with different levels of As at different geographic locations, and the impact of environmental As contamination on the soil bacterial community.

## Introduction

Arsenic (As) is a naturally occurring metalloid element present in virtually all environmental media and is a well-known carcinogen even at low levels [1, 2]. Anthropogenic activities, such as mining and smelting, use of As-containing agrochemicals, and contamination of some water supplies with As-laden groundwater have led to serious environmental pollution and public health problems in some parts of the world. The worst affected areas are in South and Southeast Asia, where millions of people have been exposed to high concentrations of As in drinking water extracted from shallow tube-wells [3]. Large volumes of As-contaminated groundwater have also been used to irrigate paddy rice, resulting in As accumulation in the paddy soil and elevated transfer of As to the food chain [4, 5].

Recent studies have shown that rice is the most significant dietary source of inorganic As for populations consuming rice as a staple food [5, 6]. This is because the generally anaerobic conditions in the paddy environment are conducive to As reduction and methylation, leading to As mobilization and increased uptake by rice plants [7, 8]. It is now clear that microorganisms play a key role in the transformation of As, including arsenite (As(III)) oxidation, arsenate (As(V)) respiration, As(V) reduction, and As(III) methylation, thereby having an impact on the As geochemical cycle and posing an environmental risk [9, 10]. As speciation in rice grains exhibits a distinct geographic pattern, with rice produced in the southern USA containing proportionally more organic As and Asian rice more inorganic As. This pattern is thought to be linked to different microbial communities in paddy soils in different regions [11]. On the other hand, elevated levels of As in soil can impose a selection pressure on soil microbial communities [12, 13]. Thus, it is important to analyze the microbial populations and functional genes associated with As transformation in soil.

An elevated abundance of As in the environment can result in genetic modifications in microorganisms. Among the modifications is the arsenic resistance (*ars*) system, which is widely distributed and has been extensively studied. The core genes of the *ars* systems encode the transcriptional repressor ArsR, the arsenite efflux pump ArsB, and the major arsenate reductase ArsC [14]. In addition, ArsA, which acts as the catalytic subunit of the ArsAB arsenite extrusion pump [15], has been identified as an additional component of the *ars* system [16]. As(III) oxidation, which is catalyzed by As(III) oxidases (comprising the AoxA and AoxB subunits, which have been renamed as AioA and AioB, respectively), is also to be a detoxification pathway in microorganisms. Methylation, which is catalyzed by the arsenite *S*-adenosylmethionine methyltransferase (ArsM) enzyme, has been proposed as an additional detoxification strategy [17]. It was shown that As contamination and soil conditions exerted strong selective pressures on soil microbial communities [18–20]. Although previous studies had focused on the functional gene abundance and diversity associated with arsenic transformation or overall functional microbial composition using qPCR [21, 22] or a functional gene array (GeoChip 3) [19, 20], to date, there are few in-depth, comprehensive investigations of the whole microbial community and their functional potential in response to soil physicochemical parameters and variation in a given geographical location.

In this study, soils with different histories of As contamination were collected from diverse geographic locations. The bacterial communities and the associated As functional genes in the soils were characterized via 16S rRNA gene sequencing and GeoChip 4.0 [23], respectively. The objectives of this study were (1) to investigate the abundance and diversity of the As functional genes and structure of the bacterial community in As contaminated soils from diverse geographic locations, and (2) to understand the relationships between the bacterial community structure, As functional genes, and soil properties.

## Methods

### Ethics statement

No specific permits were required for the described field studies. No specific permissions were required for these locations/activities because sample collection did not involve endangered or protected species or privately owned location.

### Collection and characterization of soil samples

Five soils from diverse geographic locations were used in this study: two paddy soil samples from Bangladesh (Faridpur and Sonagaon, designated as B1, B2, respectively) that had been contaminated with As by irrigation of groundwater, two paddy soils collected from south-central China (Chenzhou and Qiyang, named as C1, C2, respectively), which had high concentrations of As due to nearby mining activities (C1) or geogenic weathering (C2), and one upland arable soil from the United Kingdom (Rothamsted, southeast England, referred to as UK), which had an elevated As concentration, probably due to a relatively high geogenic background. Soils were collected from the plow layer (0–20 cm). A single bulk sample of approximately 100 kg was taken from each site after the crop had been harvested and paddy water drained. For the incubation experiment, sub-samples were taken from the bulk of 100 kg soil, which had been thoroughly homogenized. In addition, three replicates were included for each soil. Fresh soils were collected from the fields and transported to our lab by a carrier. The soils were air-dried at ambient room temperature (~25°C) and sieved through a 6-mm sieve for soil property analysis. Triplicate samples were created for incubation, and the details had been described by Zhao et al. [24], in which the relationships between As methylation in soil, soil properties, and the abundance and diversity of microbial *arsM* genes were discussed. The cropping system for the sites in Bangladesh and China was double rice crops per year, with flooding during the rice growing season. The cropping system for the UK soil was a rotation of wheat and oilseed rape without irrigation.

Soil properties, including soil pH (using a soil-to-water ratio of 1:1), soil organic carbon (SOC, measured with the dichromate oxidization method), available phosphorus (by 0.5M NaHCO_3_), soil amorphous Fe (by ammonium oxalate oxalic acid extraction), soil total As (by aqua-regia digestion) and soil phosphate-extractable As (by 0.05 M NH_4_H_2_PO_4_ extraction), were determined as described previously [25] (Table A in S1 File). To induce reducing conditions in the soils, triplicate 500 g soil samples were placed in plastic pots and flooded with deionized water to maintain a 2-cm layer of standing water above the soil surface. The soils were incubated at room temperature (20–25°C) for 30 days, and the concentrations of As species (arsenate, arsenite, monomethylarsonic acid (MMA), and dimethylarsinic acid (DMA)) in the soil pore water were monitored as described by Zhao et al. [24]. Twenty grams of soil from the center of each incubation pot were collected and stored at -80°C for later DNA extraction.

### DNA extraction

Triplicate 5 g soil samples were used for total soil DNA extraction with a freeze grinding, SDS cell lysis method as described previously [26]. The crude DNA was purified using a low melting agarose gel, followed by phenol–chloroform–butanol extraction. DNA quality was assessed based on the spectrometry absorbance ratios at 260/280 nm and 260/230 nm using a NanoDrop ND-1000 Spectrophotometer (NanoDrop Technologies Inc., Wilmington, DE). DNA concentration was measured by Pico Green using a FLUOstar OPTIMA fluorescence plate reader (BMG LABTECH, Jena, Germany). DNA samples were diluted to 2 ng /μl and stored at -80°C for further sequencing analysis.

### GeoChip analysis

GeoChip 4.0, a microbial functional gene array, synthesized by NimbleGen (Madison, Wisconsin, USA) in their 12-plex format [23] was used to detect the As related functional genes within the bacterial communities of the five soils. The purified soil DNA (1 μg) was labeled with Cy-3 dye and then hybridized at 42°C for 16 h on a Hybridization Station (MAUI, BioMicro Systems, Salt Lake City, UT) [23]. Low quality spots (signal intensity < 1000) were removed prior to statistical analysis as described previously [23]. Spots were scored as positive if the signal-to-noise ratio (SNR) was ≥ 2.0 and the coefficient of variation (CV) of the background was < 0.8. Genes that were detected in only one sample were also removed. The signal intensity of each spot was normalized by the mean intensity of the slide. The quantified microarray data were preprocessed using the microarray analysis pipeline on our website (http://ieg.ou.edu/microarray/) as described previously [27] and stored on our lab ftp server (ftp://129.15.40.240:8187/yunfu_gu/GeoChip_data_gu.xlsx).

### Amplification and MiSeq sequencing of 16S rRNA gene amplicons

The primer pair 515F (5’-GTGCCAGCMGCCGCGGTAA-3’) and 806R (5’- GGACTACHVGGGT- WTCTAAT- 3’), combined with an adapter sequence and barcode sequences for sample differentiation, were used to amplify the V4 region of the 16S rRNA gene [28]. The PCR reaction mixture consisted of 5 μl of 10 × AccuPrime PCR buffer II (INVITROGEN), 1 μl of each primer (10 μM), 5 μl template DNA and 0.2 μl AccuPrime High Fidelity Taq Polymerase, and 38.8 μl sterile water added to a final volume of 50 μl. Three replicates of amplification were performed for each sample. The PCR procedure was as follows: initial denaturation at 94°C for 1 min, followed by 32 cycles of 94°C for 20 s, 53°C for 25 s, and 68°C for 45 s, with final extension at 68°C for 10 min. PCR products (3 μl) were examined by 1% agarose gel electrophoresis, and then 5 μl of triplicate reactions were combined and quantified with PicoGreen. About 200 ng PCR products from each sample were pooled and purified with a QIAquick PCR Purification Kit (QIAGEN), and then re-quantified with PicoGreen. Denaturation was performed by mixing 10 μl of combined PCR products (2 nM) and 10 μl 0.1 N NaOH. Denatured DNA was diluted to 6 pM and mixed with an equal volume of 6 pM PhiX library. Finally, the 600 μl mixture was loaded into the reagent cartridge and run on a MiSeq sequencer (Illumina) for 300 cycles.

After primers and spacers were trimmed, forward and reverse sequences were combined with Flash [29]. Low quality fragments were then removed and sequences shorter than 240 bp were also trimmed before they were subjected to Chimera detection and removal by U-Chime [30]. All the sequence data were stored in our lab website (http://ieg.ou.edu/4download). OTU classification was performed using UCLUST at a 97% similarity level. An RDP classifier pipeline (http://zhoulab5.rccc.ou.edu) was used for taxonomic assignment of each OTU. OTUs with only one sequence (singleton) were not included in downstream analysis.

### Statistical analysis

The number of observed As functional genes and 16S rRNA gene species (Richness), Shannon index, and Evenness computed as the Hill's ratios between the Shannon indices and Richness were calculated to estimate within-sample diversity. The GeoChip hybridization intensity data were logarithmically transformed prior to statistical analysis. Data normality and variance homogeneity were analyzed using the Shapiro–Wilks test and the Levene’s test, respectively. Pearson correlation analysis with a Tukey's post-hoc test was used to reveal the linkage between the alpha-diversity of the whole microbial community and that of the As functional genes. Principle coordinate analysis (PCoA) with Bray-Curtis distance was used to estimate the bacterial community composition and As functional gene structure heterogeneity by using the relative abundances of OTUs or normalized gene intensity. Mantel tests and PCoA with the envfit function methods were used to evaluate the linkages between soil microbial community composition, As functional gene structure and soil properties. Adonis, Analysis of similarities (ANOSIM) and multi-response permutation procedure (MRPP) were used to test for dissimilarities between any two of the five As contaminated soils based on the null hypothesis [31]. The contributions of soil properties and geographic locations to the variances observed within the microbial communities were assessed with variance partitioning analysis (VPA). Spatial variables measured as latitude-longitude coordinates were converted into projected coordinates and were represented by a cubictrend- surface polynomial to capture broad-scale spatial trends. All the above analyses were performed using functions in the VEGAN package (v.1.15–1) [32] in R v.3.0.2 [33].

## Results

### Soil properties

The physicochemical properties that play important roles in mediating As transformation and microbial metabolism were analyzed in the five soils (Table A in S1 File) [24]. Both the soils collected from Bangladesh (B1 and B2) and the United Kingdom (UK) were silty clay loam, while the soils from China (C1 and C2) were clay. The soil sample with the highest pH was from Bangladesh (B1, pH 8.2), whereas the soil sample with the lowest pH was from the UK (pH 6.8). Total soil As and phosphate-extractable As ranged from 8.7 to 81.2 mg kg^-1^ and 0.5 to 7.9 mg kg^-1^, respectively. C1 soil had the highest concentrations amounts of total As and phosphate extractable As, whereas B2 and UK soils had the lowest concentrations of total As and phosphate extractable As, respectively. The amorphous iron in the soils varied from 610 to 3, 835 mg kg^-1^, with B1 and C2 soils having the lowest and highest concentration, respectively. The available phosphorus (AP) in the soil samples ranged from 5.9 to 15.2 mg kg^-1^. Finally, soil organic carbon (SOC) concentrations were high in the two Chinese soils (22.3−46.6 g kg^−1^), but relatively low in the UK soil (9.6 g kg^−1^).

### Composition of the soil microbial communities

**Fig 1:**
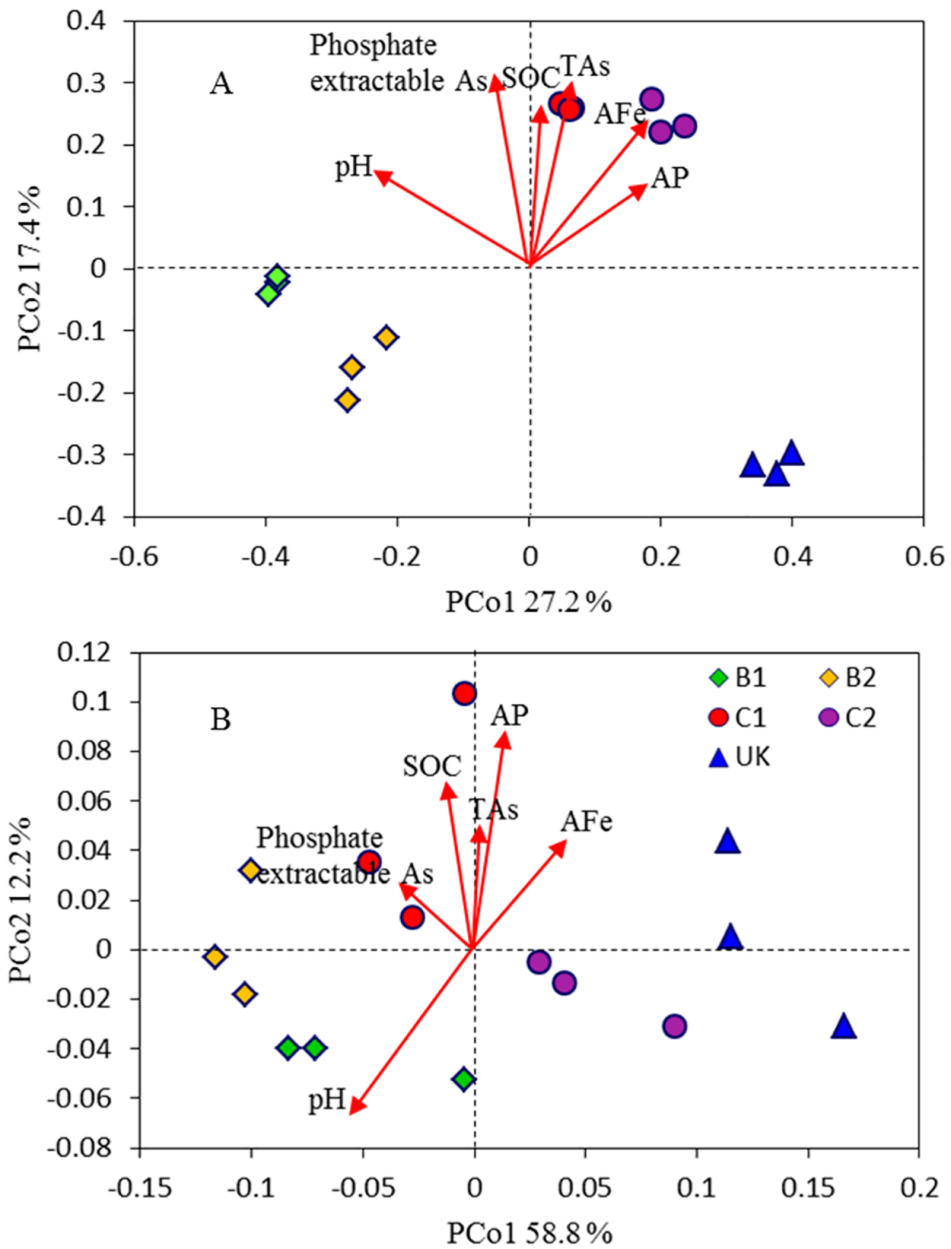
Principle coordinate analysis (PCoA) plot (environmental variables as vectors) showing differences in bacterial community structure (a) and As functional gene structure (b) of the five As contaminated soils from different geographical locations. Soil codes: B1, Faridpur, Bangladesh; B2, Sonargaon, Bangladesh; C1, Chenzhou, China; C2, Qiyang, China; UK, Rothamsted, UK.

After paired-end joining and quality trimming, 9,577 to 50,725 effective 16S rRNA gene sequences (average length of 253 bp) per sample were obtained from each replicate of soil (Table 1), resulting in a total of 396,027 sequences from all five soils. All samples were subjected to random re-sampling and rarefaction at 9,577 sequences. OTU analysis at a 97% similarity level resulted in 31,308 OTUs, of which 11,885 remained after singleton removal and were used in downstream analysis. PCoA of the 16S rRNA gene sequences was performed to determine the overall variation among bacterial communities (Fig 1A). The results showed that bacterial community structures differed based on the geographical location. The PCoA plot explained 44.6% of the observed variation, with the first axis explaining 27.2% of the variations and separating the C1 and C2 soils from the B1, B2 and UK soils. The second axis explained 17.4% of the variation and separated the B1, B2 from UK soils. Results from the dissimilarity tests based on the Adonis, Anosim and MRPP algorithms indicated significant differences (P<0.01) between geographical locations as well (Table B in S1 File).

**Table 1:** Diversity indices based on the 16S rRNA gene sequences from the five geographically distributed soils contaminated with different levels of arsenic. See Fig 1 caption for the soil codes.

Soil ID	No. of sequences^a^	Richness^b^	Evenness^c^	*H*^d^
B1	28450±17842a	2966±69a	0.92±0.00a	7.39±0.04a
B2	22440±7748a	2888±102a	0.92±0.00a	7.36±0.05a
C1	31263±17284a	2717±4a	0.89±0.00b	7.09±0.01b
C2	20162±10758a	2345±92b	0.88±0.00c	6.81±0.05c
UK	29692±16741a	1757±127c	0.86±0.01d	6.44±0.09d

Note: Means ± SD in columns followed the same letter(s) are not statistically significant at 5% significance level.

^a^ Detected sequence number.

^b^ Detected OTU number.

^c^ Evenness index.

^d^ Shannon-Wiener index: higher numbers represent higher levels of diversity.

### Arsenic related functional genes

GeoChip 4.0, which contains 48 probes for *arsA*, 84 for *arsB*, and 570 for *arsC*, was used to assess the abundance and diversity of the *ars* genes in the five soils. There were 369 positive *arsC* probes (203 to 348 detected in each sample), 54 *arsB* (27 to 51 in each sample), and 26 *arsA* (14 to 21 targets in each sample) among the five soils. GeoChip 4.0 also contains 68 probes for *arsM*. Details regarding the method of detection of *arsM* genes in the soils used in this study can be found in Zhao et al. [24]. Briefly, the *arsM* gene copy number varied from 0.4 × 10^7^ to 2.3 × 10^7^ g^−1^ dry soils. Between 27 and 35 *arsM* gene targets were detected in the five soils. There are also 133 *aoxB* (*aioB*) gene probes in GeoChip 4.0, among which 74 (47 to 66 in each sample) were detected among the five soils. The *arsM* and *aoxB* gene targets were the most numerous in the soils from Bangladesh, whereas the UK soil had the lowest number of these two genes. Similar to bacterial communities, the PCoA results based on the GeoChip data showed that the five soils differed from each other (Fig 1B), indicating that the As functional communities varied based on geographical location, which was also verified by the dissimilarity tests using the Adonis, ANOSIM and MRPP algorithms (Table B in S1 File).

Both the numbers and structure of the As functional genes (*ars*, *aoxB* and *arsM*) in the paddy soils with high As concentrations were different from those in the non-paddy soils with low As concentrations. A total of 560 As functional genes were detected in the five soils. Among these, 357–517 were detected in paddy soil samples; whereas 311–342 were detected in the UK soil samples (Table C in S1 File). In total, 329 As functional genes including *arsC* (GI 144944757) derived from *Geobacter bemidjiensis* Bem, *Sphingomonas* sp. SKA58 (GI 94497023) and *Acidovorax* sp. JS42 (GI 120607444), and *aoxB* from *Acidovorax* sp. 75 (GI 162568505) were shared by all five soils. Moreover, 49 As functional genes were only observed in all of the paddy soils (B1, B2, C1 and C2) while 24 were detected in the Bangladeshi soils (B1 and B2) (Table C in S1 File). No unique As functional genes were detected in the UK soil (Table C in S1 File). Only two unique *arsC* genes including GI 168992628 derived from *Lysinibacillus sphaericus* C3-41 and GI 117610002 derived from *Magnetococcus* sp. MC-1 were detected in soil C1. In contrast, several As functional genes, such as *arsC* derived from *Myxococcus xanthus* DK 1622 (GI 108463091) and *Burkholderia multivorans* ATCC 17616 (GI 189348441), *arsB* derived from *Sulfurovum* sp. NBC37-1 (GI 152992971), *arsA* from *Rhizobium leguminosarum* bv. viciae 3841 (GI 116254241), *arsM* from *Desulfotomaculum acetoxidans* DSM 771 (GI 257779458), and *aoxB* from *Chloroflexus* sp. Y-400-fl (GI 222524598) were detected only in the Bangladeshi soils (Table C in S1 File). All types of the As functional genes detected in the UK soil with a low As content were also detected in at least one of the four paddy soils with high available As contents, suggesting that the UK soil contained a less-diverse subset of the As transformation genes. Finally, the Mantel test analyses showed that the structures of functional genes involved in arsenite oxidation (*aoxB*) and arsenite methylation (*arsM*) were positively and significantly correlated with those involved in arsenite efflux (*arsA* and *arsB*) and arsenate reduction (*arsC*), respectively (Table D in S1 File).

### Soil microbial community diversity

To assess the internal (within soils) complexity of individual microbial populations, the mean values of OTU Richness (the observed OTUs number), Shannon-Wiener index (*H*) and Evenness were calculated based on the 16S rRNA gene data (Table 1). The average Richness ranged from 1757 to 2966 across the five soils. The highest Richness was in soil B1 while the lowest was in the UK soil. Similar trends in Evenness and Shannon-Wiener index were also observed (Table 1).

The As functional genes Richness (the observed gene species), Shannon-Wiener index (*H*) and Evenness were also evaluated (Table E in S1 File). These indices followed a trend similar as the 16S rRNA genes, although Evenness remained relatively constant across all samples. Pearson correlation analysis showed that the As functional gene Richness (the observed gene species), Shannon- Wiener index (*H*) and Evenness correlated positively with those of the 16S rRNA gene (P<0.05) (Fig A in S1 File).

### Changes in the bacterial community composition

**Fig 2:**
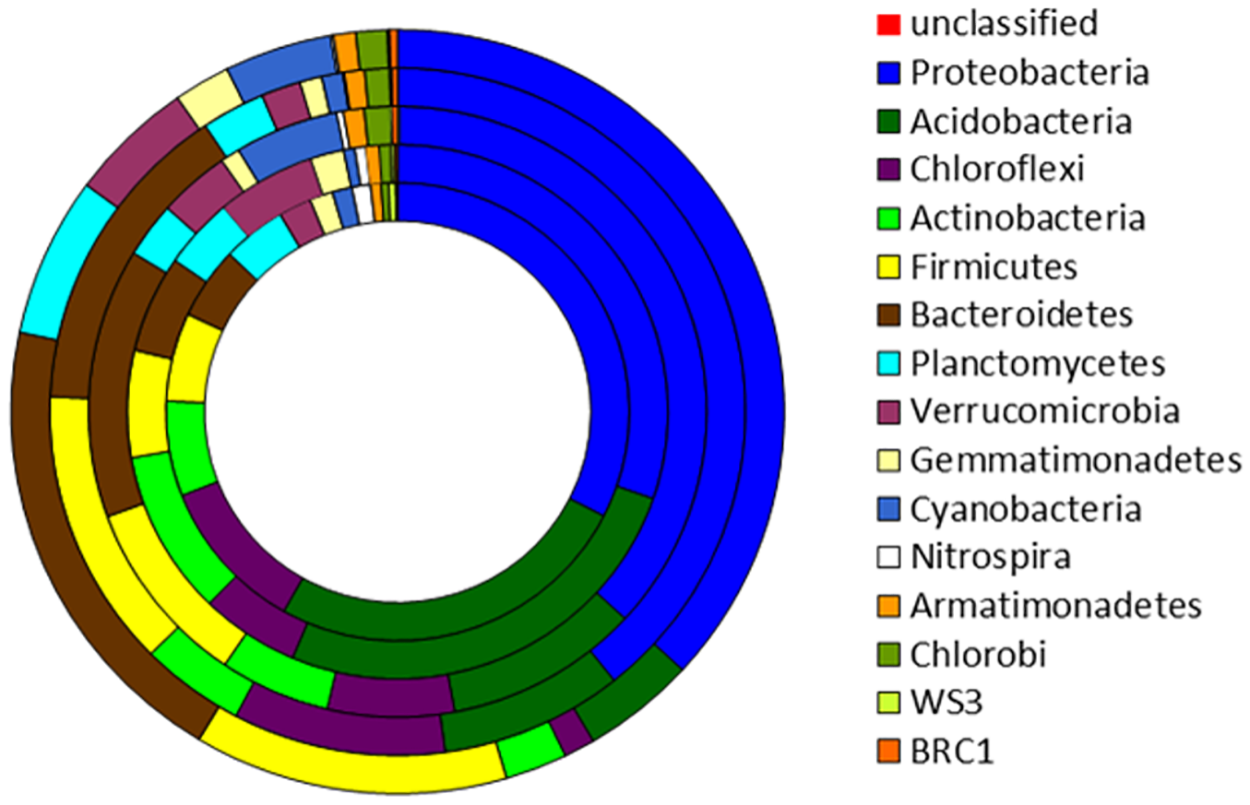
Composition of microbial communities in the five soils at the phylum level. Circles from inside out correspond to the soils B1, B2, C1, C2 and UK, respectively. See Fig 1 caption for the soil codes.

Fig 2 summarizes the relative bacterial community abundance at the phylum level for each soil. *Proteobacteria* was the dominant phylum for all samples, accounting for between 29% and 38% of detected OTUs. However, different soils had distinct subdominant phyla (phyla with greatest abundance other than *Proteobacteria*). For soil B1, *Acidobacteria*, *Chloroflexi* and *Actinobacteria* were the subdominant groups, comprising 24%, 10%, and 6.4%, respectively, of the sequences detected. *Acidobacteria*, *Actinobacteria* and *Firmicutes* were the subdominant groups for soil B2, comprising 25%, 9.7%, and 6.4%, respectively. The subdominant groups from soil C1 were *Bacteroidetes*, *Acidobacteria* and *Firmicutes*, comprising 13%, 9.8%, and 9.6%, respectively. In soil C2, *Bacteroidetes*, *Firmicutes* and *Chloroflexi* were the subdominant groups, comprising 15%, 13%, and 9.6%, respectively. For the UK soil, *Bacteroidetes*, *Firmicutes* and *Planctomycetes* were the subdominant groups, comprising 19%, 13%, and 6.7%, respectively. These phyla represented approximately 69% to 78% of bacteria detected within the five soils.

**Fig 3:**
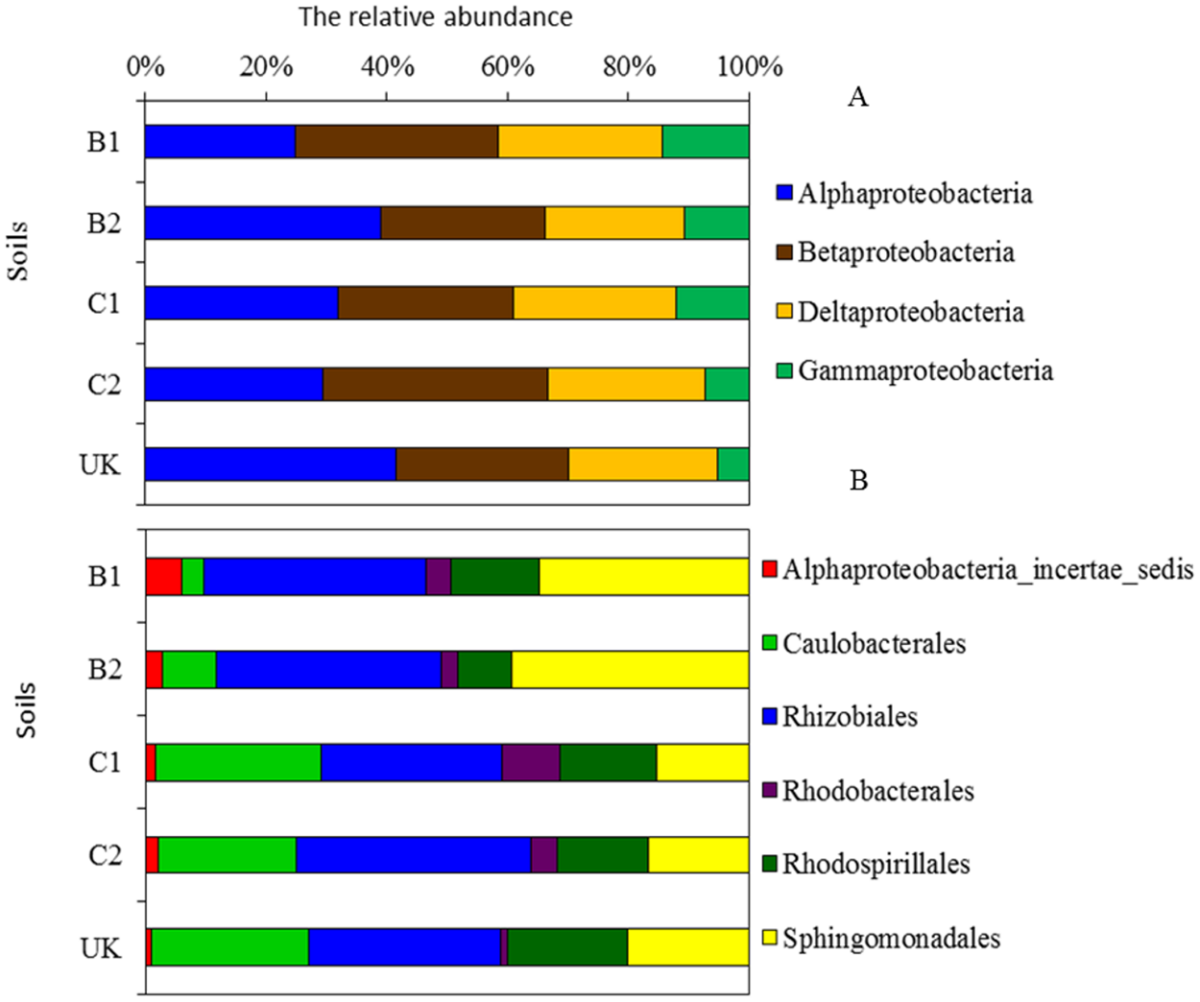
Relative abundance of the *Proteobacteria* community composition in the five soils. A, The relative abundance of the *Proteobacteria*; B, Relative abundance of the *Alphaproteobacteria*. See Fig 1 caption for the soil codes.

The *Alphaproteobacteria* comprised 25 to 42% of the *Proteobacteria*, followed by *Betaproteobacteria* (27 to 37%), *Deltaproteobacteria* (23 to 27%), and *Gammaproteobacteria* (5 to14%) (Fig 3A). Within the *Alphaproteobacteria*, six taxa were identified. *Rhizobiales* was the dominant group (29 to 37%) for all 5 soils, followed by *Sphingomonadales* and *Caulobacterales*, representing 15 to 39% and 9 to 20% of each population, respectively. *Alphaproteobacteria incertae sedis*, *Rhodobacterales* and *Rhodospirillales* comprised 14 to 27% of the *Alphaproteobacteria* (Fig 3B). The relative abundance of *Rhizobiales* was the highest in UK soil with the lowest available As content, but was the lowest was in B1 soil with a high available As content.

Table F in S1 File lists the orders of bacteria detected. A total of 76 orders were identified by the RDP classifier (Table F in S1 File). Of these, 53 (60 to 76% of the classified sequences) were shared by all soils, including *Anaerolineales*, *Rhodocyclales*, *Sphingomonadales*, *Rhizobiales*, *Clostridiales*, *Burkholderiales*, *Myxococcales*, *Planctomycetales*, *Sphingobacteriales*, and *Anaerolineales*. Two orders (*Thermogemmatisporales*, *Herpetosiphonales*) only appeared in soil B2 while others such as *Erysipelotrichales*, *Methanomicrobiales*, *Chlamydiales*, *Rubrobacterales*, and *Desulfarculales*, were only detected in the paddy soils (B1, B2, C1 and C2).

At the family level, a total of 174 families were obtained (Table G in S1 File). One hundred and twelve families (occupying 96 to 98% of the classified sequences), including *Anaerolineaceae*, *Chitinophagaceae*, *Planctomycetaceae*, *Sphingomonadaceae*, *Gemmatimonadaceae*, *Cystobacteraceae*, *Clostridiaceae 1*, *Geobacteraceae*, *Rhodocyclaceae*, *Hyphomicrobiaceae*, *Comamonadaceae*, *Oxalobacteraceae*, *Caulobacteraceae*, were shared by all soils. There were 17 families that appeared in only one soil and accounted for less than 1% of the classified sequences. Among those families, 15 were observed in the paddy soils while two were in the UK soil.

**Fig 4:**
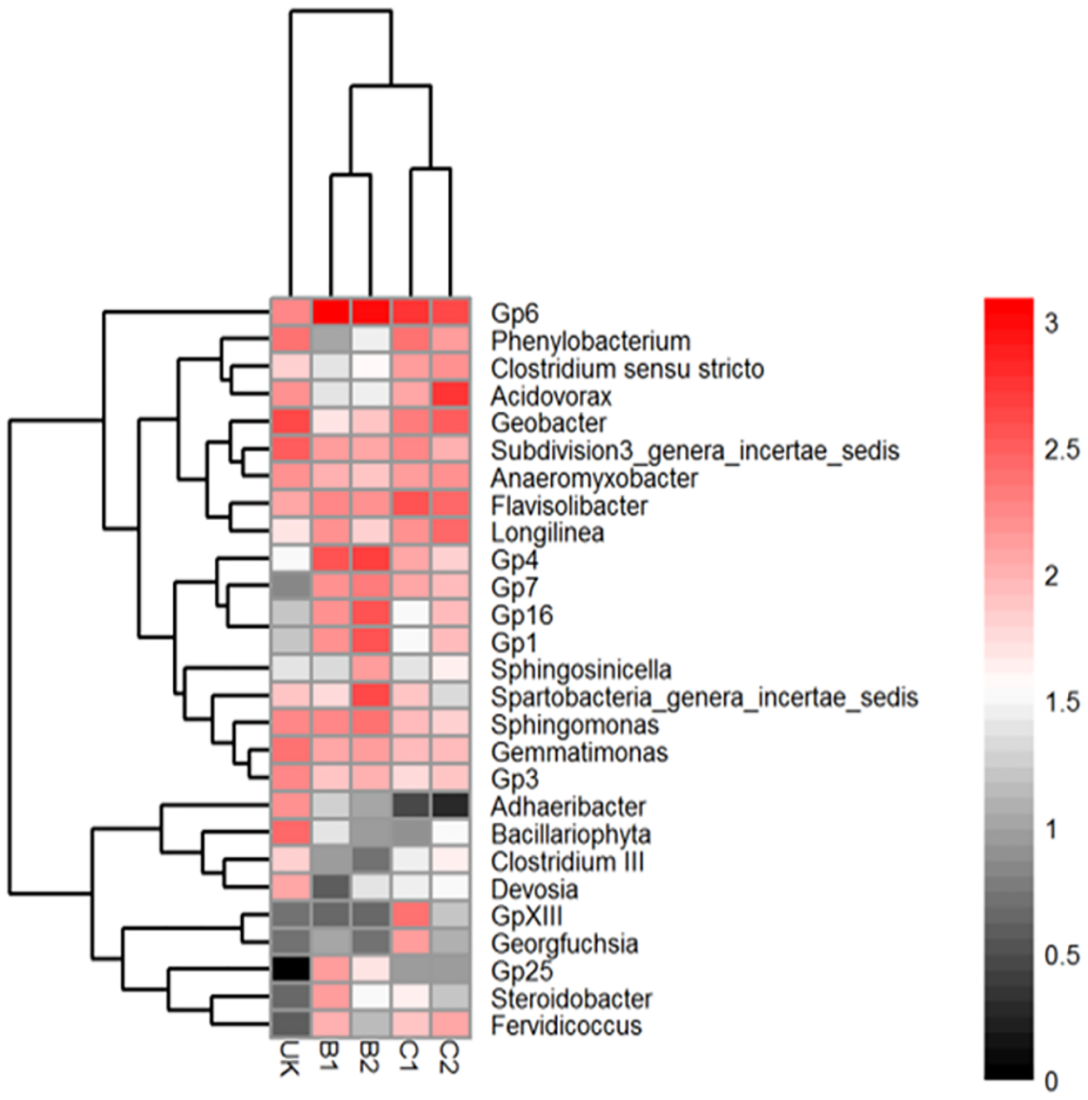
Heatmap of the 10 most abundant genera in each soil. The 10 most abundant genera in each sample were selected (a total of 27 genera for all five soils), and their abundances were compared to those in other soils. The color intensity in each cell shows the percentage of a genus in a soil. See Fig 1 caption for the soil codes.

The abundance of the 544 genera detected is summarized in Table H in S1 File. Among the assigned 544 genera, 224 were shared by all soils and accounted for 52 to 66% of the classified sequences. There were 68 rare genera that were observed in only one sample, which accounted for < 0.5% of the total classified genera sequences in each sample. Among the rare genera, 60 were only observed in the four paddy soils while 8 were only in the non-paddy soil. The 10 most abundant genera in each soil (a total of 27 genera for all 5 soils) were compared across the five soils (Fig 4). Eight genera were abundant (> 0.5%) in all five soils, including *Gp*6, *Phenylobacterium*, *Acidovorax*, *Geobacter*, *Subdivision*3 *genera incertae sedis*, *Anaeromyxobacter*, *Sphingomonas*, and *Flavisolibacter*.

### Relationships between bacterial communities, As potential function and environmental parameters

All soil physical-chemical parameters correlated significantly with the soil bacterial communities at the taxonomic level based on the PCoA data (P < 0.05) (Table I in S1 File). Moreover, the Mantel test analyses showed that the soil bacterial taxonomic structure correlated significantly with soil pH (P = 0.039) and soil phosphate extractable As (P = 0.001) (Table J in S1 File). For As functional genes, PCoA data showed that soil pH was the most significantly correlated variable with soil microbial communities at the functional gene level (Table I in S1 File). In addition, the Mantel test analyses further revealed that the bacterial As functional gene structure correlated significantly with soil pH (P = 0.006), soil phosphate extractable As (P = 0.008) and soil amorphous Fe (P = 0.022) (Table J in S1 File).

**Fig 5:**
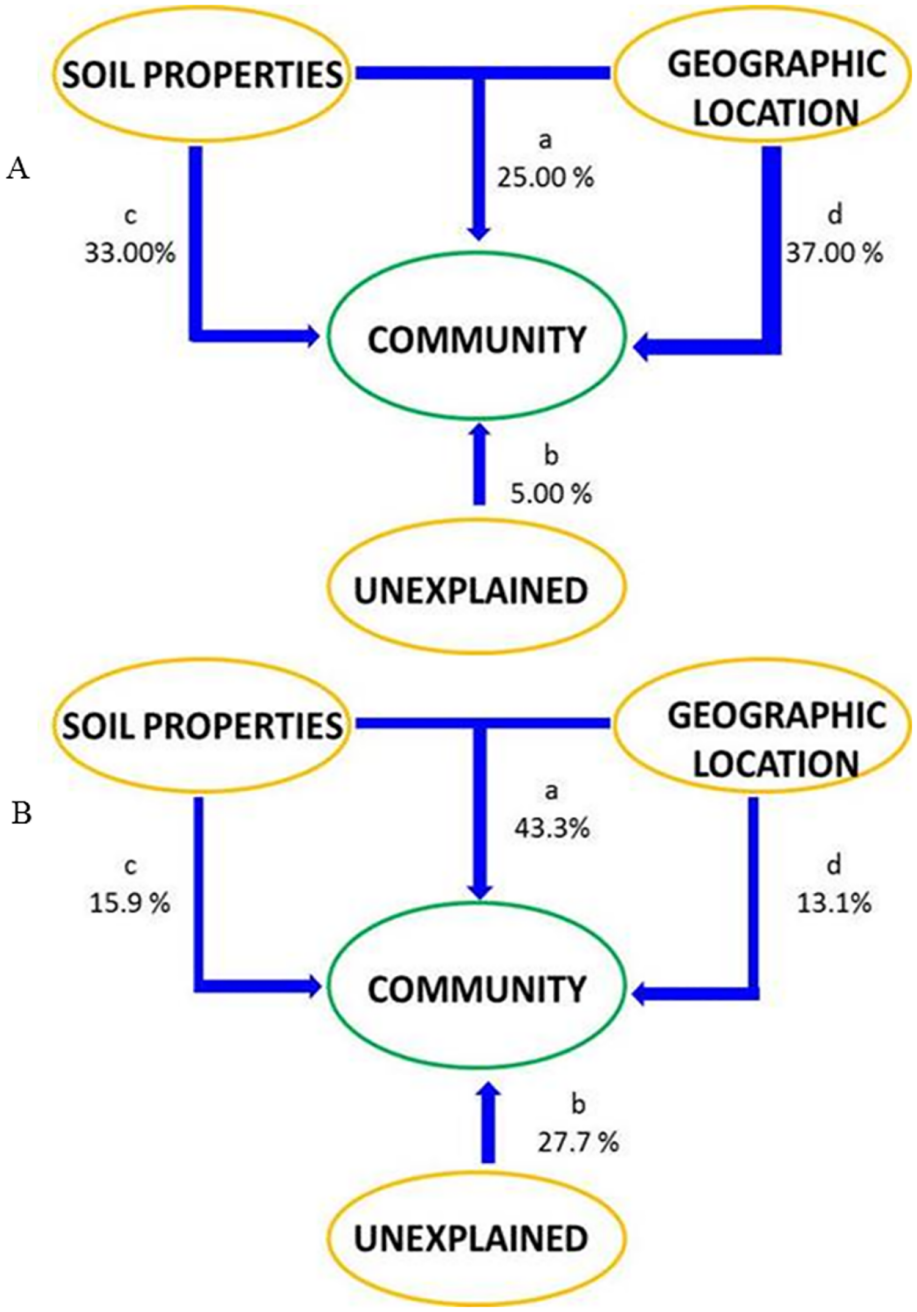
Variation partitioning analyses of As functional genes (A) and microbial community (B) explained by the soil selected properties and geographic locations. The diagram represents the biological variation partitioned into the relative effects of each factor or a combination of factors, in which arrow thickness was proportional to the respective percentages of explained variation. Letter ′a′represents the combined effect of soil properties and geographic location. Letter ′b′represents the effect that could not be explained by any of the variables tested. And letters ′c′ and ′d′ represent the effect of soil properties and geographic location, respectively.

Variance partitioning analysis (VPA) was used to further evaluate the contribution of selected soil parameters and geographic location to the bacterial community composition and As functional gene structure (Fig 5). Based on the 16S rRNA gene MiSeq data, a total of 95% of the variation was explained by the soil parameters and geographic location (Fig 5a), which could independently explained 33.0% and 37.0% of the variation of bacterial communities, respectively. Interactions between these two components explained another 25% of the variation. Only 5% of the community variance could not be explained by these two components and their interactions. For GeoChip analyses, a total of 72% of the variation was explained by the soil parameters and geographic location (Fig 5b), which independently explained 16% and 13%, respectively, of the total variations observed. Interactions between these two factors (43.3%) seemed to have more of an effect than the individual factors. About 28% of the community variation could not be explained by these two environmental variables.

## Discussion

It is well known that the soil type and texture significantly influences As mobility and toxicity. In regions with soils derived from the same parent material, soil texture is often the dominant soil feature affecting background levels of As [34]. Fine-textured clay soils tend to result in a lower As mobility compared to coarse textured sandy soils due to a higher sorptive capacity[35, 36]. The anaerobic conditions of flooded paddy soils favor As mobilization because of the reductive dissolution of iron oxyhydroxides and the reduction of arsenate to arsenite. The paddy soils examined in this study had a higher pH, soil organic matter, soil total As and soil phosphate- extractable As than the upland soil from the UK. The paddy soils from Bangladesh are a silty clay loam while those from China were clay. The concentrations of soil organic carbon, soil total As and phosphate-extractable As were higher in the Bangladeshi soils than those in Chinese soils.

The higher diversity of the As related functional genes (e.g. *arsC*,*arsB*, *arsA*, *arsM* and *aoxB*) in the soils with higher As concentrations in the current study was not unexpected, because high levels of As may exert a strong selective pressure leading to increased diversity of As-related genes. Moreover, studies of microbial communities using similar GeoChip microarray or metagenomics indicated an overabundance of metal resistance genes in metal-rich environments [37, 38]. Poirel et al. found strong positive correlations between changes in *arsB* gene abundance and As levels using real-time PCR [22]. Significant correlations were also observed in studying the response of bacterial communities to the chronic chromium and arsenic contamination in Pakistan using pyrosequencing and quantitative PCR [39]. In addition, in the soils studied, other soil properties such as pH represent additional stressors for microbial communities that may impact their diversity. Metagenomics studies also showed that microbial-mediated As metabolic processes in paddy soils correlated significantly with pH [40]. Zhang et al. [41] revealed a strong correlation between *arsC* and *arsM* gene copies based on quantitative PCR, and suggested that similar compositions of microbial communities were involved in As(V) reduction and As(III) methylation in paddy soils. Previous studies revealed that the As(V) reduction, As(III) oxidation, and As(III) methylation processes co-exist and are closely related in microbes in paddy soils [41–43]. Similarly, significant correlations between the structures of the *ars*, *aioB*, and *arsM* genes were also observed in this study, suggesting that these different As transformation systems co-exist and are closely related in microbes in both paddy and upland soil.

The alpha-diversity of 16S rRNA genes were higher in the soils with a high level of phosphate-extractable As than in those with a low level of phosphate-extractable As. The higher diversity and abundance were observed at each taxonomic level examined. This was probably due to an evolutionary adaptation of some specific bacterial species to the As contamination stress [44]. Cai et al. (2009) reported that more bacterial species isolated from a long term high-level arsenic contamination site than from intermediate and low levels of arsenic contamination sites [18]. Turpeinen et al. (2004) also found that the diversity of arsenic-resistant bacteria in higher arsenic-, chromium- and copper-contaminated soil was higher than that in less contaminated soil [45]. The higher alpha-diversity of the soil microbial community in the more As contaminated soil suggests that many microbial species could cope with the long term As stress. Genus like *Steroidobacter* had higher relative abundance in the heavy metal contaminated soils [46]. Similarly, in our study, the relative abundance of the genus *Steroidobacter* in the soil contaminated with more As was also higher than that in the lower As containing soil (Fig 4). The presence of these bacteria suggests that they may play an important role in As contaminated due to their high adaptability to extreme environments [47], providing a stabilizing effect for the soil functions.

All the soils were dominated by *Proteobacteria*. Previous studies chromium and As contaminated studies showed that *Proteobacteria* are frequently present in metal contaminated sites and are capable of metal transformation. Odum proposed that *r*-selected organisms (rapidly reproducing) [48], such as *Proteobacteria* [49], could be stimulated after a stressor is applied to an ecosystem, which would explain their dominance. Within *Proteobacteria*, the alpha and beta subdivision were the predominant groups, which was in line with the study results by Han [50]. Bacteria belonging to the *Alphaproteobacteria* were identified as the major microbial communities in contaminated soils while the *Betaproteobacteria* would disappear in soils under high As stress. In our study, other highly abundant phyla included *Acidobacteria* and *Bacteroidetes*, members of which are broadly represented in arsenic contaminated soils and sediments and have similar ecological distributions [51–53]. Previous studies revealed that lineages of *Acidobacteria* and *Bacteroidetes* are significantly regulated by soil parameters such as pH and organic matter, respectively [51, 53]. Other soil properties such as C/N, NO_3_^-^ and the availability of trace elements can also influence microbial community composition in arsenic contaminated soils [20, 54]. Similarly, significant correlations between the abundance of these two phyla and soil pH were also observed in present study.

Several bacterial lineages that play critical roles in soil As transformation were observed in the four paddy soils (e.g. B1, B2, C1 and C2) with relatively high level of available As. These included bacteria within the orders *Erysipelotrichales*, *Methanomicrobiales*, *Chlamydiales*, *Rubrobacterales*, and *Desulfarculales*, which are typically associated with the anaerobic conditions found in paddy soils and play important roles in As–methylation and volatilization [54]. In addition, bacteria from the families *Rhodothermaceae*, *Methanobacteriaceae*, *Alicyclobacillaceae* are critical to the natural arsenic transformation [55–57]. Several genera, such as *Thiobacter*, *Cytophaga*, *Nitrosomonas*, *Desulfomonile*, *Defluviicoccus*, *Azotobacter*, *Desulfatirhabdium*, and *Anaeroarcus* are widely distributed in arsenic contaminated soils. *Thiobacter* can gain energy from the oxidation of arsenic and are ubiquitous in arsenic- contaminated environments [58]. *Cytophaga*—*Flavobacterium* related groups capable of methylating As have been isolated from various arsenic-rich soils and sediments [59]. *Desulfomonile tiedjei*, a sulfate-reducing bacteria, is also widely distributed in arsenic-rich acid mine drainage [60]. Taken together, these results indicate that the paddy soils with higher available As content harbored more arsenic transforming bacterial species than the upland soil with a low level of available As.

The 16S rRNA gene sequencing data were found to be generally in line with the functional gene in the present study. For example, the *arsC* gene (GI 144944757) retrieved from the *G*. *bemidjiensis* Bem was detected in all the five soils, whilst 16S rRNA gene sequencing analysis also revealed the presence of *Geobacter*. *Geobacter* species, members of the order *Desulfuromonadales*, are able to mediate high rates of arsenite release in various As-contaminated environments [61]. Moreover, several *arsC* genes derived from the members of genus *Sphingomonas* were detected in all the five soil, whilst the presence of *Sphingomonas* was confirmed or by 16S rRNA gene sequence data. Besides, the genera *Acidovorax* and related members, which are aerobic heterotrophs and often resistant to arsenic [62], were detected in all five soils based on the16S rRNA gene MiSeq sequencing and GeoChip analysis. *Rhodobacter* species are photosynthetic with the capability of As reduction [63]. Hassan et al (2015) detected the *Rhodobacter*-like *aioA* sequences in the arsenic-contaminated anaerobic groundwater in Bangladesh, but no *Rhodobacter* 16S rRNA gene sequences were observed [61]. In contrast, in our study, both *Rhodobacter*-like *arsC* and *Rhodobacter* 16S rRNA gene sequences were detected in all five soils. Interestingly, *Cytophaga hutchinsonii*, member of the genus *Cytophaga*, in which the *arsM* and *arsC* genes were both detected, were found only in the soils with high available As (e.g. B1, B2, C1, and C2). This result also matched the 16S rRNA gene sequencing data.

Understanding the environmental variables that affect the microbial community structure is a key goal in microbial ecology [64]. Soil geochemical variables like soil As, soil phosphorus, soil organic matter, soil nitrate concentration, *etc*. are vital factors in shaping microbial communities [16, 24]. In our study, soil pH, soil available As, and soil amorphous Fe (free iron oxides) were found to be the most important variables. Several studies have found these and similar variables to be important as well. Sheik et al (2012) found that the structure, diversity and abundance of microbial communities were highly influenced by the presence and concentration of As [39]. Similar results have also been obtained for rhizosphere and non-rhizosphere communities of the As-hyperaccumulator *Pteris vittata* [19]. Similar results were also obtained by Hornstrom [65]. Previous studies had revealed that soil pH is a key factor in shaping soil microbial diversity and composition since the intracellular pH of most microorganisms is usually within 1 pH unit of neutrality, and any significant deviation in environmental pH value would impose stress on microbes [66]. Iron (hydr) oxides have been reported to be an important abiotic factor in shaping the microbial community in As contaminated soils because these oxides provide the most important sorption phase for As [67]. Some arsenite tolerant, anaerobic ferrous iron-oxidizing bacteria such as *Geobacter* could produce highly crystalline ferric iron minerals that were only slowly reduced by iron-reducing bacteria and thus stimulate the permanent immobilization of arsenic [62, 68]. Zhang et al (2015) reported that the concentration of total Fe plays a role in shaping the microbial communities involved in respiratory As (V) reduction [44]. Considering that the ferric iron and arsenate are electron acceptors for a range of anaerobic heterotrophic microbes, the positive relations between iron concentration and As release may indicate that iron- and arsenic-cycling microorganisms are widely presented and co-exist in paddy soils in anaerobic conditions [61].

Geographic location was also important in controlling both the bacterial community structure and functional gene structure, although overall, pH appeared to be a stronger driver (Fig 5). Compared to the bacterial community, soil geochemical variables and geographic location explained a lower percentage of the variation in the As functional gene structure. This could be due to the soil parameters such as soil pH and As concentration would apply a selective pressure on the microbial community, which could increase the similarity between spatially isolated communities. Similar to the previous studies, the PCoA results in this study were in accordance with the dissimilarity analysis results, showing the soils from different geographic locations formed separate clusters. This pattern suggests a significant impact of local abiotic environmental conditions on the composition and structures of microbial communities. It should be noted that the sampling strategy and the relatively small number of samples analyzed in this study may have limited our ability to fully separate the effects of environmental variables and geographic locations. More replicate samples should be collected for study of the bacterial community structures in As-contaminated soils across different geographic locations.

## Conclusions

In summary, both functional gene array (GeoChip 4.0) and next generation sequencing of 16S rRNA genes were applied to analyze the soil microbial communities and As functional gene communities from diverse geographic locations and different levels of As contamination. The study revealed that the bacterial taxonomic composition and As functional gene composition were often related in As contaminated soils from diverse geographic locations. Soil properties (i.e. soil pH, soil available As and soil amorphous Fe) as well as graphical locations were found to be significantly factors in shaping the soil microbial community composition and As-related functional gene structure. This study provides valuable insight into the variation of the abundance and diversity of the As functional gene structure and the bacterial community composition in As contaminated soils from diverse geographic locations, and their relations to environmental variables.

## Supporting information

S1 File Fig A. The correlation between alpha-diversity of microbial community and that of As functional genes. The alpha-diversity was calculated by richness. The Pearson correlation coefficient (r) and the significance level (*P*). Table A. Characteristics of the five geographically distributed soils contaminated with different arsenic. Table B. Statistical analysis of differences in the microbial community composition and structure among the five As contaminated soils based on GeoChip data and Illumina MiSeq sequencing data. Table C. As functional genes detected in each soil. Table D. Mantel test of the relationship between the structure of genes involved in As methylation (*arsM*), oxidation (*aoxB*) and arsenite efflux (*arsA* and *arsB*) and arsenate reduction (*arsC*). Table E. Diversity indices of the As functional genes of the five geographically distributed soils. Table F. Sequences of all orders in each soil. Table G. Sequences of all families in each soil. Table H. Sequences of all genera in each soil. Table I. Principle coordinate analysis (PCoA) of GeoChip and 16S rRNA gene sequencing data with environmental properties among five arsenic contaminated soils from different geographic locations. Table J. Mantel tests of GeoChip and 16S sequencing data with environmental properties among five arsenic contaminated soils from different geographic locations.
(DOCX)

## References

[1] Bhattacharya P, Welch AH, Stollenwerk KG, McLaughlin MJ, Bundschuh J, Panaullah G. Arsenic in environment: biology and chemistry. Sci Total Environ. 2007; 379: 109–120. 10.1016/j.scitotenv.2007.02.037 17434206

[2] Duker AA, Carranza EJM, Hale M. Arsenic geochemistry and health. Environ Int. 2005; 31: 631–641. 10.1016/j.envint.2004.10.020 15910959

[3] Brammer H. Ravenscroft P. Arsenic in groundwater: a threat to sustainable agriculture in South and South-east Asia. Environ Inter. 2009; 35: 647–654. 10.1016/j.envint.2008.10.004 19110310

[4] Dittmar J, Voegelin A, Roberts LC, Hug SJ, Saha GC, Ali MA, et al Arsenic accumulation in a paddy field in Bangladesh: seasonal dynamics and trends over a three-year monitoring period. Environ Sci Technol. 2010; 44: 2925–2931. 10.1021/es903117r 20235529

[5] Meharg AA, Williams PN, Adomako E, Lawgali YY, Deacon C, Villada A, et al Geographical variation in total and inorganic arsenic content of polished (white) rice. Environ Sci Technol. 2009; 43: 1612–1617. 19350943 10.1021/es802612a

[6] Li G, Sun GX, Williams PN, Nunes L, Zhu YG. Inorganic arsenic in Chinese food and its cancer risk. Environ Inter. 2011; 37: 1219–1225. 10.1016/j.envint.2011.05.007 21632110

[7] Xu XY, McGrath SP, Meharg A, Zhao FJ. Growing rice aerobically markedly decreases arsenic accumulation. Environ Sci Technol. 2008; 42: 5574–5579. 18754478 10.1021/es800324u

[8] Zhao FJ, McGrath SP, Meharg AA. Arsenic as a food-chain contaminant: mechanisms of plant uptake and metabolism and mitigation strategies. Ann Rev Plant Biol. 2010; 61: 535–559. 20192735 10.1146/annurev-arplant-042809-112152

[9] Corsini A, Cavalca L, Zaccheo P, Crippa L, Andreoni V. Influence of microorganisms on arsenic mobilization and speciation in a submerged contaminated soil: Effects of citrate. Appl Soil Ecol. 2011; 49: 99–106.

[10] Jia Y, Huang H, Chen Z, Zhu YG. Arsenic uptake by rice is influenced by microbe-mediated arsenic redox changes in the rhizosphere. Environ Sci Technol. 2014; 48: 1001–1007. 10.1021/es403877s 24383760

[11] Zhao FJ, Zhu YG, Meharg AA. Methylated arsenic species in rice: Geographical variation, origin, and uptake mechanisms. Environ Sci Technol. 2013; 47: 3957–3966. 10.1021/es304295n 23521218

[12] Lami R, Jones LC, Cottrell MT, Lafferty BJ, Ginder-Vogel M, Sparks DL, et al Arsenite modifies structure of soil microbial communities and arsenite oxidization potential. FEMS Microbiol Ecol. 2013; 84: 270–279. 10.1111/1574-6941.12061 23252611

[13] Páez-Espino D, Tamames J, de Lorenzo V, Cánovas D. Microbial responses to environmental arsenic. BioMetals. 2009; 22:117–130. 10.1007/s10534-008-9195-y 19130261

[14] Rosen BP. Biochemistry of arsenic detoxification. FEBS Lett. 2002; 529: 86–92. 12354618 10.1016/s0014-5793(02)03186-1

[15] Rosen BP. Families of arsenic transporters. Trends Microbiol. 1999; 7: 207–212. 10354596 10.1016/s0966-842x(99)01494-8

[16] Wu J, Rosen BP. The *arsD* gene encodes a second trans-acting regulatory protein of the plasmid-encoded arsenical resistance operon. Mol Microbiol. 1993; 8: 615–623. 8326869 10.1111/j.1365-2958.1993.tb01605.x

[17] Qin J, Rosen BP, Zhang Y, Wang GJ, Franke S, Rensing C. Arsenic detoxification and evolution of trimethylarsine gas by a microbial arsenite S-adenosylmethionine methyltransferase. Proc Natl Acad Sci USA. 2006; 103: 2075–2080. 10.1073/pnas.0506836103 16452170 PMC1413689

[18] Cai L, Liu GH, Rensing C, Wang GJ. Genes involved in arsenic transformation and resistance associated with different levels of arsenic-contaminated soils. BMC Microbiol. 2009; 9: 4 10.1186/1471-2180-9-4 19128515 PMC2631446

[19] Xiong JB, Wu LY, Tu SX, Van Nostrand JD, He ZL, Zhou JZ, et al Microbial communities and functional genes associated with soil arsenic contamination and the rhizosphere of the arsenic-hyper accumulating plant *Pteris vittata* L. Appl Environ Microbiol. 2010; 76: 7277–7284. 10.1128/AEM.00500-10 20833780 PMC2976218

[20] Xiong JB, He ZL, Van Nostrand JD, Luo GS, Tu SX, Zhou JZ, et al Assessing the microbial community and functional genes in a vertical soil Profile with long-term arsenic contamination. PLoS ONE. 2012; 7: e50507 10.1371/journal.pone.0050507 23226297 PMC3511582

[21] Escudero LV, Casamayor EO, Chong G, Pedrós-Alió C, Demergasso C. Distribution of microbial arsenic reduction, oxidation and extrusion genes along a wide range of environmental arsenic concentrations. PLoS ONE. 2013; 8: e78890 10.1371/journal.pone.0078890 24205341 PMC3815024

[22] Poirel J, Joulian C, Leyval C, Billard P. Arsenite-induced changes in abundance and expression of arsenite transporter and arsenite oxidase genes of a soil microbial community. Res Microbiol. 2013; 164: 457–465. 10.1016/j.resmic.2013.01.012 23396038

[23] Tu QC, Yu H, He ZL, Deng Y, Wu LY, Van Nostrand JD, et al GeoChip 4: a functional gene-array-based high-throughput environmental technology for microbial community analysis. Mol Ecol Resource. 2014; 14: 914–928. 10.1111/1755-0998.12239 24520909

[24] Zhao FJ, Harris E, Yan J, Ma JC, Wu LY, Liu WJ, et al Arsenic methylation in soils and its relationship with microbial *arsM* abundance and diversity, and As speciation in rice. Environ Sci Technol. 2013; 47: 7147–7154. 10.1021/es304977m 23750559

[25] Stroud JL, Khan MA, Norton GJ, Islam MR, Dasgupta T, Zhu YG, et al Assessing the labile arsenic pool in contaminated paddy soils by isotopic dilution techniques and simple extractions. Environ Sci Technol. 2011; 45: 4262–4269. 10.1021/es104080s 21504212

[26] Zhou JZ, Bruns MA, Tiedje JM. DNA recovery from soils of diverse composi tion. Appl Environ Microbiol. 1996; 62: 316–322. 8593035 10.1128/aem.62.2.316-322.1996 PMC167800

[27] Zhao MX, Xue K, Wang F, Liu SS, Bai SJ, Sun B, et al Microbial mediation of biogeochemical cycles revealed by simulation of global changes with soil transplant and cropping. ISME J. 2014; 8: 2045–2055. 10.1038/ismej.2014.46 24694714 PMC4184012

[28] Caporaso JG, Lauber CL, Walters WA, Berg-Lyons D, Huntley J, Fierer N, Owens SM, et al Ultra-high-throughput microbial community analysis on the Illumina HiSeq and MiSeq platforms. ISME J. 2012; 6: 1621–1624. 10.1038/ismej.2012.8 22402401 PMC3400413

[29] Magoč T, Salzberg SL. FLASH: fast length adjustment of short reads to improve genome assemblies. Bioinformatics. 2011; 27: 2957–2963. 10.1093/bioinformatics/btr507 21903629 PMC3198573

[30] Edgar RC, Haas BJ, Clemente JC, Quince C, Knight R. UCHIME improves sensitivity and speed of chimera detection. Bioinformatics. 2011; 27: 2194–2200. 10.1093/bioinformatics/btr381 21700674 PMC3150044

[31] Anderson M J. A new method for non-parametric multivariate analysis of variance. Austral Ecol. 2001; 26: 32–46.

[32] Oksanen J, Blanchet FG, Kindt R, Legendre P, Minchin PR, O'Hara RB, et al. Vegan: community ecology package. R package version 2.0–3. http://CRAN.R-project.org/package=vegan. 2012.

[33] Team RDC. R: a language and environment for statistical computing, reference index version 2.9.2. R Foundation for Statistical Computing, Vienna, Austria ISBN 3–900051 -07-0, URL http://www.r-project.org. 2005.

[34] Huang YC. Arsenic distribution in soils In: Nriagu JO, editor. Arsenic in the environment, part I: cycling and characterization. New York: John Wiley and Sons, Inc 1994; P: 17–49.

[35] Lombi E., Sletten RS, Wenzel WW. Sequentially extracted arsenic from different size fractions of contaminated soils. Water Air Soil Pollut. 2000; 124: 319–332.

[36] Walsh LM., Sumner ME, Keeney DR. Occurrence and distribution of arsenic in soils and plants. Environ Health Perspect. 1977; 19: 67–71. 908315 10.1289/ehp.771967 PMC1637429

[37] Hemme CL, Deng Y, Gentry TJ, Fields MW, Wu LY, Barua S, et al Metagenomic insights into evolution of a heavy metal- contaminated groundwater microbial community. ISME J. 2010; 4: 660–672. 10.1038/ismej.2009.154 20182523

[38] Reith F, Brugger J, Zammit CM, Gregg AL, Goldfarb KC, Andersen GL, et al Influence of geogenic factors on microbial communities in metallogenic Australian soils. ISME J. 2012; 6: 2107–2118. 10.1038/ismej.2012.48 22673626 PMC3475370

[39] Sheik CS, Mitchell TW, Rizvi FZ, Rehman Y, Faisal M, Hasnain S, et al Exposure of soil microbial communities to chromium and arsenic alters their diversity and structure. PLoS ONE. 2012; 6: 1–13. 10.1371/journal.pone.0040059 PMC3386950 22768219

[40] Xiao KQ, Li LG, Ma LP, Zhang SY, Bao P, Zhang T, Zhu YG. Metagenomic analysis revealed highly diverse microbial arsenic metabolism genes in paddy soils with low-arsenic contents. Environ Pollut. 2016; 211: 1–8. 10.1016/j.envpol.2015.12.023 26736050

[41] Zhang SY, Zhao FJ, Sun GX, Su JQ, Yang XR, Li H, Zhu YG. Diversity and abundance of arsenic biotransformation genes in paddy soils from southern China. Environ Sci Technol. 2015; 49: 4138–4146. 10.1021/acs.est.5b00028 25738639

[42] Zhu YG, Yoshinaga M, Zhao FJ, Rosen BP. Earth abides arsenic biotransformations. Annu Rev Earth Planet Sci. 2014; 42: 443–467. 10.1146/annurev-earth-060313-054942 26778863 PMC4712701

[43] Xiao KQ, Li LG, Ma LP, Zhang SY, Bao P, Zhang T, Zhu YG. Metagenomic analysis revealed highly diverse microbial arsenic metabolism genes in paddy soils with low-arsenic contents. Environ Pollut. 2016; 211: 1–8. 10.1016/j.envpol.2015.12.023 26736050

[44] Pérez-de-Mora A, Burgos P, Madejón E, Cabrera F, Jaeckel P, Schloter M. Microbial community structure and function in a soil contaminated by heavy metals: effects of plant growth and different amendments. Soil Biol Biochem. 2006; 38: 327–341.

[45] Turpeinen R, Kairesalo T, Häggblom MM. Microbial community structure and activity in arsenic-, chromium- and copper-contaminated soils. FEMS Microbiol Ecol. 2004; 47: 39–50. 10.1016/S0168-6496(03)00232-0 19712345

[46] Yin HQ, Niu JJ, Ren YH, Cong J, Zhang XX, Fan FL, et al An integrated insight into the response of sedimentary microbial communities to heavy metal contamination. Sci Rep. 2015; 5: 14266 10.1038/srep14266 26391875 PMC4585741

[47] Spain AM, Krumholz LR, Elshahed MS. Abundance, composition, diversity and novelty of soil *Proteobacteria*. ISME J. 2009; 3: 992–1000. 10.1038/ismej.2009.43 19404326

[48] Odum EP. Trends expected in stressed ecosystems. BioScience. 1985; 35: 419–422.

[49] Fierer N, Bradford MA, Jackson RB. Toward an ecological classification of soil bacteria. Ecology. 2007; 88: 1354–1364. 17601128 10.1890/05-1839

[50] Han YH, Fu JW, Xiang P, Cao Y, Rathinasabapathi B, Chen YS, Ma LQ. Arsenic and phosphate rock impacted the abundance and diversity of bacterial arsenic oxidase and reductase genes in rhizosphere of As-hyperaccumulator *Pteris vittata*. J Hazard Mater. 2017; 321: 146–153. 10.1016/j.jhazmat.2016.08.079 27619960

[51] Jackson CR, Dugas SL, Harrison KG. Enumeration and characterization of arsenate-resistant bacteria in arsenic free soils. Soil Biol Biochem. 2005; 37: 2319–2322.

[52] Macur RE, Jackson CR, Botero LM, McDermott TR, Inskeep WP. Bacterial populations associated with the oxidation and reduction of arsenic in an unsaturated soil. Environ Sci Technol. 2004; 38: 104–111. 14740724 10.1021/es034455a

[53] Nemergut DR, Martin AP, Schmidt SK. Integron diversity in heavy-metal contaminated mine tailings and inferences about integron evolution. Appl Environ Microbiol. 2004; 70: 1160–1168. 10.1128/AEM.70.2.1160-1168.2004 14766601 PMC348930

[54] Frankenberger W. Short communication effects of trace elements on arsenic volatilization. Soil Biol Biochem. 1998; 30: 269–274.

[55] Engel AS, Johnson LR, Porter ML. Arsenite oxidase gene diversity among *Chloroflexi* and *Proteobacteria* from El Tatio Geyser Field, Chile. FEMS Microbiol Ecol. 2013; 83: 745–756. 10.1111/1574-6941.12030 23066664

[56] McBride BC, Wolfe RS. Biosynthesis of dimethylarsine by *Methanobacteriu- m*. Biochemistry. 1971; 10: 4312–4317. 5126942 10.1021/bi00799a024

[57] van der Merwe JA, Deane SM, Rawlings DE. The chromosomal arsenic resistance genes of *Sulfobacillus thermosulfidooxidans*. Hydrometallurgy. 2010; 104: 477–482.

[58] Bryan CG, Marcha M, Battaglia-Brunet F, Kugler V, Lemaitre-Guillier C, Lièvremont D, et al Carbon and arsenic metabolism in Thiomonas strains: differences revealed diverse adaptation processes. BMC Microbiol. 2009; 9: 127 10.1186/1471-2180-9-127 19549320 PMC2720973

[59] Honschopp S, Brunken N, Nehrhorn A, Breunig HJ. Isolation and characterization of a new arsenic methylating bacterium from soil. Microbiol Res. 1996; 151: 37–41. 8857265 10.1016/s0944-5013(96)80053-x

[60] Bruneel O, Duran R, Casiot C, Elbaz-Poulichet F, Personne JC. Diversity of microorganisms in Fe-As-rich acid mine drainage waters of Carnoulès, France. Appl Environ Microbiol. 2006; 72: 551–556. 10.1128/AEM.72.1.551-556.2006 16391091 PMC1352176

[61] Hassan Z, Sultana M, van Breukelen BM, Khan SI, Wilfred FMR. Diverse arsenic- and iron-cycling microbial communities in arsenic-contaminated aquifers used for drinking water in Bangladesh. FEMS Microbiol Ecol. 2015; 91: fiv026. 10.1093/femsec/fiv026 25778510

[62] Lear G, Song B, Gault AG, Polya DA, Lloyd JR. Molecular analysis of arsenate-reducing bacteria within Cambodian sediments following amendment with acetate. Appl Environ Microb. 2007; 73: 1041–1048. 10.1128/AEM.01654-06 PMC1828664 17114326

[63] Lin HZ, Yue YH, Lü JC, Zhao GC, Yang PS. Variation in composition and relative content of accumulated photopigments in a newly isolated *Rhodobacter capsulatus* strain XJ-1 in response to arsenic. J Environ Sci Health. 2014; 49: 1493–1500 10.1080/10934529.2014.937168 25137537

[64] Liang YT, Van Nostrand JD, Deng Y, He ZL, Wu LY, Zhang X, et al Functional gene diversity of soil microbial communities from five oil- contaminated fields in China. ISME J. 2011; 5: 403–413. 10.1038/ismej.2010.142 20861922 PMC3105718

[65] Hornstrom E. Phytoplankton in 63 limed lakes in comparison with the distribution in 500 untreated lakes with varying pH. Hydrobiologia. 2002; 470: 115–126.

[66] Fierer N, Jackson RB. The diversity and biogeography of soil bacterial communities. Proc Natl Acad Sci USA. 2006; 103: 626–631. 10.1073/pnas.0507535103 16407148 PMC1334650

[67] Yang CL, Li SY, Liu RB, Sun PS, Liu K. Effect of reductive dissolution of iron (hydr) oxides on arsenic behavior in a water–sediment system: First release, then adsorption. Ecol Eng. 2015; 83: 176–183.

[68] Hohmann C, Winkler E, Morin G, Kappler A. Anaerobic Fe (II)-oxidizing bacteria show As resistance and immobilize As during Fe (III) mineral precipitation. Environ Sci Technol. 2009; 44: 94–101. 10.1021/es900708s 20039738

