# Correction: Bacterial community and arsenic functional genes diversity in arsenic contaminated soils from different geographic locations

**DOI:** 10.1371/journal.pone.0189656

**Published:** 2017-12-07

**Authors:** Yunfu Gu, Joy D. Van Nostrand, Liyou Wu, Zhili He, Yujia Qin, Fang-Jie Zhao, Jizhong Zhou

The following information is missing from the Funding section: This study was supported by the Natural Science Foundation of China (grant number 41330853).
