# Supplementary material for: Bacterial community and arsenic functional genes diversity in arsenic contaminated soils from different geographic locations
Source: PLoS One. 2017 May 5;12(5):e0176696. doi: 10.1371/journal.pone.0176696 (PMC5419559; doi:10.1371/journal.pone.0176696)
Supplement: S1 File — Fig A. The correlation between alpha-diversity of microbial community and that of As functional genes. The alpha-diversity was calculated by richness. The Pearson correlation coefficient (r) and the significance level (P). Table A. Characteristics of the five geographically distributed soils contaminated with different arsenic. Table B. Statistical analysis of differences in the microbial community composition and structure among the five As contaminated soils based on GeoChip data and Illumina MiSeq sequencing data. Table C. As functional genes detected in each soil. Table D. Mantel test of the relationship between the structure of genes involved in As methylation (arsM), oxidation (aoxB) and arsenite efflux (arsA and arsB) and arsenate reduction (arsC). Table E. Diversity indices of the As functional genes of the five geographically distributed soils. Table F. Sequences of all orders in each soil. Table G. Sequences of all families in each soil. Table H. Sequences of all genera in each soil. Table I. Principle coordinate analysis (PCoA) of GeoChip and 16S rRNA gene sequencing data with environmental properties among five arsenic contaminated soils from different geographic locations. Table J. Mantel tests of GeoChip and 16S sequencing data with environmental properties among five arsenic contaminated soils from different geographic locations. (DOCX) [file pone.0176696.s001.docx]

**S1**

**Figures and Tables**

Fig A. The correlation between alpha-diversity of microbial community and that of As functional genes. The alpha-diversity was calculated by richness. The Pearson correlation coefficient (r) and the significance level (*P*).

Table A Characteristics of the five geographically distributed soils contaminated with different arsenic.

| Sample name | Location of sample | Land use | Soil texture | pH | SOC (g/kg) | AP (mg/kg) | Amorphous Fe (mg/kg) | Total As (μg/kg) | Phosphate extractable As (μg/kg) | Contamination source |
| --- | --- | --- | --- | --- | --- | --- | --- | --- | --- | --- |
| B11 | Faridpur, Bangladesh | Paddy soil | Silty clay loam | 8.22 | 15.71 | 5.91 | 610 | 21.26 | 5.51 | irrigation |
| B12 | Faridpur, Bangladesh | Paddy soil | Silty clay loam | 8.21 | 15.33 | 5.88 | 599 | 21.09 | 5.26 | irrigation |
| B13 | Faridpur, Bangladesh | Paddy soil | Silty clay loam | 8.23 | 16.07 | 5.92 | 621 | 21.43 | 5.76 | irrigation |
| B21 | Sonargoan, Bangladesh | Paddy soil | Silty clay loam | 7.43 | 13.11 | 12.1 | 914 | 8.72 | 2.68 | irrigation |
| B22 | Sonargoan, Bangladesh | Paddy soil | Silty clay loam | 7.25 | 12.99 | 12.33 | 923 | 8.87 | 3.02 | irrigation |
| B23 | Sonargoan, Bangladesh | Paddy soil | Silty clay loam | 7.61 | 13.21 | 11.67 | 905 | 8.57 | 2.33 | irrigation |
| C11 | Chenzhou, China | Paddy soil | Clay | 7.53 | 43.61 | 15.1 | 2483 | 81.24 | 7.95 | mining |
| C12 | Chenzhou, China | Paddy soil | Clay | 7.44 | 46.56 | 14.77 | 2456 | 81.09 | 8.06 | mining |
| C13 | Chenzhou, China | Paddy soil | Clay | 7.62 | 40.64 | 15.23 | 2510 | 81.39 | 7.83 | mining |
| C21 | Qiyang, China | Paddy soil | Clay | 7.44 | 23.11 | 13.1 | 3835 | 63.7 | 6.81 | geogenic |
| C22 | Qiyang, China | Paddy soil | Clay | 7.55 | 22.32 | 12.89 | 3844 | 64.55 | 6.67 | geogenic |
| C23 | Qiyang, China | Paddy soil | Clay | 7.32 | 23.88 | 13.11 | 3826 | 62.86 | 6.95 | geogenic |
| UK1 | Rothamsted, UK | Arable (upland) | Silty Clay loam | 6.82 | 9.61 | 12.1 | 1703 | 10.23 | 0.52 | geogenic |
| UK2 | Rothamsted, UK | Arable (upland) | Silty Clay loam | 6.79 | 9.56 | 12.03 | 1689 | 10.34 | 0.55 | geogenic |
| UK3 | Rothamsted, UK | Arable (upland) | Silty Clay loam | 6.85 | 9.64 | 11.97 | 1717 | 10.13 | 0.499 | geogenic |
| Note: SOC was soil organic carbon, AP was available phosphorus. | | | | | | | | | | |
|  |  |  |  |  |  |  |  |  |  |  |

Table B Statistical analysis of differences in the microbial community composition and structure among the five As contaminated soils based on GeoChip data and Illumina MiSeq sequencing data.

| Soil communities | GeoChip 4.0 | | | 16S OTUs | | |
| --- | --- | --- | --- | --- | --- | --- |
|  | MRPP.sig | ANOSIM.sig | Adonis.sig | MRPP.sig | ANOSIM.sig | Adonis.sig |
| UK vs C1 | 0.096 | 0.088 | 0.014 | 0.106 | 0.09 | 0.001 |
| UK vs C2 | 0.085 | 0.103 | 0.024 | 0.091 | 0.107 | 0.085 |
| UK vs B1 | 0.102 | 0.099 | 0.048 | 0.104 | 0.1 | 0.001 |
| UK vs B2 | 0.103 | 0.1 | 0.071 | 0.091 | 0.114 | 0.001 |
| C1 vs C2 | 0.114 | 0.092 | 0.042 | 0.079 | 0.094 | 0.018 |
| C1 vs B1 | 0.107 | 0.105 | 0.043 | 0.089 | 0.106 | 0.001 |
| C1 vs B2 | 0.099 | 0.105 | 0.051 | 0.087 | 0.091 | 0.001 |
| C2 vs B1 | 0.091 | 0.086 | 0.059 | 0.1 | 0.116 | 0.001 |
| C2 vs B2 | 0.112 | 0.099 | 0.084 | 0.094 | 0.114 | 0.008 |
| B1 vs B2 | 0.099 | 0.121 | 0.012 | 0.084 | 0.095 | 0.001 |
| total | 0.001 | 0.001 | 0.001 | 0.001 | 0.001 | 0.001 |

Note: B1 and B2 were soil samples isolated from Faridpur and Sonagaon, Bangladesh, respectively. C1 and C2 were soil samples from Chenzhou and Qiyang, China, respectively. UK was soil samples from Rothamsted, United Kingdom.

Table C As functional genes detected in each soil.

| Genbank ID | Gene | Organism | UK | C1 | C2 | B1 | B2 |
| --- | --- | --- | --- | --- | --- | --- | --- |
| 120599747 | ArsC | Shewanella sp. W3-18-1 | 1.05 | 1.02 | 1.01 | 1.10 | 1.05 |
| 89350403 | ArsC | Xanthobacter autotrophicus Py2 | 0.61 | 0.89 | 0.89 | 0.94 | 0.63 |
| 24346017 | ArsC | Shewanella oneidensis MR-1 | 0.00 | 0.96 | 0.00 | 0.97 | 1.01 |
| 57238575 | ArsC | Campylobacter jejuni RM1221 | 0.97 | 0.99 | 0.96 | 0.99 | 0.99 |
| 70729553 | ArsC | Pseudomonas fluorescens Pf-5 | 0.00 | 0.59 | 0.00 | 0.00 | 0.61 |
| 162568595 | aoxB | uncultured bacterium | 1.01 | 1.01 | 1.06 | 1.08 | 1.05 |
| 94554288 | ArsC | Deinococcus geothermalis DSM 11300 | 0.00 | 0.60 | 0.00 | 0.91 | 0.93 |
| 114307475 | ArsC | Nitrosomonas eutropha C91 | 0.00 | 0.57 | 0.00 | 0.00 | 0.61 |
| 119822199 | ArsC | Stenotrophomonas maltophilia R551-3 | 0.99 | 1.02 | 1.00 | 1.04 | 1.12 |
| 86164313 | aoxB | Marinomonas sp. MED121 | 0.85 | 0.90 | 0.89 | 0.91 | 0.94 |
| 197104901 | ArsC | Phenylobacterium zucineum HLK1 | 0.00 | 0.92 | 0.00 | 0.96 | 0.99 |
| 119952628 | ArsC | Arthrobacter aurescens TC1 | 0.00 | 0.61 | 0.00 | 0.00 | 0.63 |
| 83576241 | ArsC | Rhodospirillum rubrum ATCC 11170 | 1.03 | 0.98 | 1.05 | 1.04 | 1.02 |
| 162568511 | aoxB | Pseudomonas sp. 73 | 0.91 | 0.92 | 0.92 | 0.92 | 0.91 |
| 151561416 | ArsC | Ochrobactrum anthropi ATCC 49188 | 0.90 | 0.65 | 0.60 | 0.97 | 0.65 |
| 74422173 | ArsC | Nitrobacter winogradskyi Nb-255 | 0.95 | 0.95 | 0.93 | 0.96 | 0.96 |
| 94497023 | ArsC | Sphingomonas sp. SKA58 | 1.01 | 1.03 | 1.05 | 1.09 | 1.06 |
| 95111342 | ArsC | Pseudomonas entomophila L48 | 1.09 | 1.20 | 1.16 | 1.15 | 1.21 |
| 41395431 | ArsC | Mycobacterium avium subsp. paratuberculosis K-10 | 0.00 | 0.98 | 0.92 | 0.95 | 1.00 |
| 111020357 | arsB | Rhodococcus sp. RHA1 | 0.00 | 0.62 | 0.00 | 0.62 | 0.97 |
| 284015962 | arsM | Haloterrigena turkmenica DSM 5511 | 0.00 | 0.61 | 0.00 | 0.61 | 0.95 |
| 219997419 | ArsC | Thioalkalivibrio sp. HL-EbGR7 | 0.98 | 0.98 | 1.02 | 1.02 | 1.00 |
| 182636182 | ArsC | Beijerinckia indica subsp. indica ATCC 9039 | 1.13 | 1.18 | 1.17 | 1.17 | 1.17 |
| 90903257 | aoxB | uncultured bacterium | 0.90 | 0.61 | 0.60 | 0.96 | 0.94 |
| 107102033 | ArsC | Pseudomonas aeruginosa PACS2 | 0.00 | 0.91 | 0.00 | 0.64 | 1.03 |
| 283843001 | arsM | Rhodopseudomonas palustris DX-1 | 1.00 | 1.01 | 1.01 | 1.02 | 1.01 |
| 118720134 | ArsC | Burkholderia multivorans ATCC 17616 | 0.00 | 0.59 | 0.00 | 0.60 | 0.60 |
| 91703634 | ArsC | Mycobacterium sp. MCS | 1.12 | 1.06 | 1.09 | 1.14 | 1.11 |
| 83815482 | arsM | Salinibacter ruber DSM 13855 | 1.02 | 1.00 | 1.06 | 1.09 | 1.07 |
| 133738533 | ArsA | Herminiimonas arsenicoxydans | 0.95 | 0.93 | 0.97 | 0.98 | 0.64 |
| 162568547 | aoxB | uncultured bacterium | 0.91 | 0.92 | 0.91 | 0.93 | 0.99 |
| 126355143 | ArsC | Pseudomonas putida GB-1 | 1.05 | 1.08 | 1.09 | 1.05 | 1.10 |
| 220710253 | ArsC | Comamonas testosteroni KF-1 | 0.96 | 1.01 | 1.00 | 1.02 | 0.97 |
| 73669221 | arsM | Methanosarcina barkeri str. Fusaro | 0.00 | 0.62 | 0.60 | 0.91 | 0.99 |
| 90903267 | aoxB | uncultured bacterium | 1.01 | 1.05 | 1.05 | 1.07 | 1.07 |
| 91697317 | ArsC | Polaromonas sp. JS666 | 0.00 | 0.62 | 0.00 | 0.60 | 0.95 |
| 23492988 | ArsC | Corynebacterium efficiens YS-314 | 0.00 | 0.63 | 0.00 | 0.62 | 0.97 |
| 91802638 | aoxB | Nitrobacter hamburgensis X14 | 0.00 | 0.89 | 0.00 | 0.95 | 0.95 |
| 194292621 | ArsC | Cupriavidus taiwanensis | 0.62 | 0.95 | 0.90 | 0.99 | 1.00 |
| 152993065 | ArsC | Sulfurovum sp. NBC37-1 | 0.62 | 0.61 | 0.63 | 0.98 | 0.96 |
| 162568567 | aoxB | uncultured bacterium | 0.64 | 0.98 | 0.62 | 0.97 | 0.66 |
| 192360117 | ArsC | Cellvibrio japonicus Ueda107 | 0.57 | 0.92 | 0.57 | 0.90 | 0.95 |
| 212707618 | ArsC | Providencia rustigianii DSM 4541 | 0.00 | 0.91 | 0.63 | 0.93 | 0.98 |
| 217331421 | aoxB | Ochrobactrum tritici | 0.94 | 0.67 | 0.63 | 0.65 | 1.02 |
| 77920195 | ArsC | Pelobacter carbinolicus DSM 2380 | 1.02 | 1.00 | 1.03 | 1.02 | 1.00 |
| 119670771 | ArsC | Azoarcus sp. BH72 | 0.00 | 0.00 | 0.00 | 0.60 | 0.91 |
| 171089977 | ArsC | Geobacillus sp. WCH70 | 0.60 | 0.93 | 0.93 | 0.96 | 0.95 |
| 125973625 | ArsC | Clostridium thermocellum ATCC 27405 | 0.00 | 0.60 | 0.85 | 0.59 | 0.57 |
| 158304027 | ArsC | Acaryochloris marina MBIC11017 | 0.58 | 0.98 | 0.94 | 0.65 | 1.04 |
| 121530665 | ArsC | Ralstonia pickettii 12J | 0.92 | 0.97 | 0.91 | 0.93 | 1.00 |
| 149277880 | arsM | Pedobacter sp. BAL39 | 0.98 | 1.03 | 0.96 | 1.00 | 1.04 |
| 110623048 | arsB | Zimmermannella faecalis | 0.91 | 0.90 | 0.92 | 0.93 | 0.96 |
| 220932303 | ArsC | Halothermothrix orenii H 168 | 0.67 | 1.01 | 0.68 | 0.97 | 0.97 |
| 77465140 | ArsC | Rhodobacter sphaeroides 2.4.1 | 0.95 | 1.04 | 1.02 | 1.02 | 1.09 |
| 110668350 | ArsA | Haloquadratum walsbyi DSM 16790 | 0.86 | 1.02 | 0.94 | 0.98 | 1.05 |
| 150274182 | ArsC | Bacteroides capillosus ATCC 29799 | 0.64 | 0.66 | 0.96 | 0.97 | 0.95 |
| 67088759 | ArsC | Azotobacter vinelandii AvOP | 0.92 | 1.00 | 0.98 | 1.02 | 1.02 |
| 162568505 | aoxB | Acidovorax sp. 75 | 0.90 | 0.95 | 0.91 | 0.66 | 0.97 |
| 161378972 | ArsC | Oceanibulbus indolifex HEL-45 | 1.01 | 0.99 | 1.03 | 1.05 | 1.03 |
| 10175620 | ArsC | Bacillus halodurans C-125 | 0.00 | 0.94 | 0.93 | 0.97 | 0.94 |
| 117613120 | ArsC | Shewanella sp. ANA-3 | 1.11 | 1.16 | 1.17 | 1.15 | 1.14 |
| 39936617 | ArsC | Rhodopseudomonas palustris CGA009 | 1.10 | 1.11 | 1.10 | 1.12 | 1.13 |
| 133740357 | ArsC | Herminiimonas arsenicoxydans | 0.90 | 0.95 | 0.93 | 0.98 | 0.95 |
| 83951805 | ArsC | Roseovarius nubinhibens ISM | 0.91 | 1.00 | 0.93 | 1.00 | 1.02 |
| 33669028 | ArsC | Rhodococcus erythropolis | 0.95 | 0.95 | 0.96 | 1.00 | 0.97 |
| 83836813 | ArsC | Roseovarius nubinhibens ISM | 0.00 | 0.95 | 0.92 | 1.01 | 1.05 |
| 149810275 | ArsC | Roseobacter sp. AzwK-3b | 1.11 | 1.12 | 1.11 | 1.11 | 1.14 |
| 20516009 | ArsC | Thermoanaerobacter tengcongensis MB4 | 0.00 | 0.00 | 0.00 | 0.00 | 0.94 |
| 90020547 | ArsC | Saccharophagus degradans 2-40 | 0.00 | 0.97 | 0.00 | 0.92 | 0.97 |
| 116665020 | ArsC | Methanosaeta thermophila PT | 1.06 | 1.08 | 1.05 | 1.06 | 1.12 |
| 219719739 | ArsC | Stenotrophomonas sp. SKA14 | 1.07 | 1.17 | 1.11 | 1.11 | 1.18 |
| 18892543 | ArsC | Pyrococcus furiosus DSM 3638 | 0.00 | 0.59 | 0.84 | 0.89 | 0.89 |
| 211960597 | ArsC | Pseudovibrio sp. JE062 | 0.00 | 0.59 | 0.00 | 0.00 | 0.90 |
| 113473873 | ArsC | Sphingomonas sp. KA1 | 0.65 | 0.63 | 0.92 | 0.99 | 0.60 |
| 7769067 | arsB | Acidithiobacillus ferrooxidans | 0.00 | 0.60 | 0.00 | 0.00 | 0.91 |
| 148835866 | ArsC | Vibrio shilonii AK1 | 0.00 | 0.59 | 0.00 | 0.00 | 0.92 |
| 90104153 | ArsC | Rhodopseudomonas palustris BisB18 | 1.07 | 1.11 | 1.09 | 1.05 | 1.12 |
| 79075659 | aoxB | Agrobacterium tumefaciens | 0.66 | 0.67 | 0.69 | 0.00 | 1.01 |
| 118001378 | ArsC | Comamonas testosteroni KF-1 | 0.00 | 0.91 | 0.59 | 0.63 | 0.95 |
| 33469591 | ArsC | Alcaligenes faecalis | 0.60 | 0.95 | 0.91 | 1.00 | 0.66 |
| 110621602 | arsB | uncultured methanogenic archaeon | 0.59 | 0.93 | 0.62 | 0.93 | 0.96 |
| 77019723 | ArsC | Rhodococcus erythropolis PR4 | 1.13 | 1.24 | 1.17 | 1.20 | 1.24 |
| 119857783 | ArsC | Pseudomonas putida W619 | 0.00 | 0.61 | 0.60 | 0.97 | 0.97 |
| 113932190 | ArsC | Caulobacter sp. K31 | 0.97 | 1.00 | 1.02 | 0.71 | 1.06 |
| 145631127 | ArsC | Haemophilus influenzae 22.4-21 | 0.93 | 0.98 | 0.97 | 1.02 | 0.66 |
| 116252631 | ArsC | Rhizobium leguminosarum bv. viciae 3841 | 0.87 | 0.91 | 0.89 | 0.95 | 0.91 |
| 163745788 | ArsC | Oceanibulbus indolifex HEL-45 | 1.01 | 0.98 | 1.03 | 1.05 | 1.03 |
| 3128310 | ArsC | Rhodobacter capsulatus | 1.07 | 1.08 | 1.07 | 1.05 | 1.06 |
| 85703886 | aoxB | Roseovarius sp. 217 | 0.95 | 0.96 | 0.95 | 0.99 | 0.97 |
| 110623052 | arsB | Tsukamurella strandjordii | 0.97 | 0.92 | 0.95 | 1.01 | 0.96 |
| 68511363 | ArsC | Rubrobacter xylanophilus DSM 9941 | 1.09 | 1.15 | 1.13 | 1.11 | 1.16 |
| 151575350 | ArsC | Ralstonia pickettii 12D | 0.00 | 0.95 | 0.63 | 0.97 | 1.04 |
| 213923415 | ArsC | Pseudomonas syringae pv. tomato T1 | 0.96 | 0.96 | 0.96 | 0.67 | 0.65 |
| 162568573 | aoxB | uncultured bacterium | 1.07 | 1.11 | 1.13 | 1.13 | 1.13 |
| 219994748 | arsM | Thioalkalivibrio sp. HL-EbGR7 | 0.93 | 0.62 | 0.95 | 0.98 | 0.94 |
| 188533207 | ArsC | Erwinia tasmaniensis Et1/99 | 0.56 | 0.88 | 0.00 | 0.59 | 0.88 |
| 162568495 | aoxB | Variovorax sp. 4-2 | 0.62 | 0.96 | 0.61 | 0.63 | 0.94 |
| 197715087 | ArsC | Streptomyces sviceus ATCC 29083 | 0.64 | 0.66 | 0.96 | 0.99 | 0.94 |
| 223690011 | ArsC | Desulfobacterium autotrophicum HRM2 | 0.89 | 0.91 | 0.63 | 0.90 | 0.93 |
| 209883189 | ArsC | Oligotropha carboxidovorans OM5 | 0.97 | 1.01 | 1.00 | 1.05 | 1.02 |
| 196243766 | ArsC | Cyanothece sp. PCC 8802 | 1.02 | 0.97 | 1.02 | 1.04 | 1.01 |
| 108771904 | arsB | Mycobacterium sp. MCS | 1.02 | 1.10 | 1.02 | 1.08 | 1.14 |
| 83840222 | ArsC | Sulfitobacter sp. NAS-14.1 | 1.02 | 1.02 | 1.02 | 1.06 | 1.03 |
| 73748096 | ArsC | Dehalococcoides sp. CBDB1 | 1.06 | 1.04 | 1.06 | 1.04 | 1.02 |
| 158520779 | arsM | Desulfococcus oleovorans Hxd3 | 0.58 | 0.93 | 0.61 | 0.93 | 0.96 |
| 118730373 | ArsC | Delftia acidovorans SPH-1 | 1.09 | 1.04 | 1.11 | 1.12 | 1.08 |
| 157409956 | ArsC | Methylobacterium populi BJ001 | 1.00 | 1.07 | 1.05 | 0.73 | 1.15 |
| 163259055 | ArsC | Bordetella petrii | 0.00 | 0.92 | 0.00 | 0.00 | 0.97 |
| 190571993 | ArsC | Comamonas sp. CNB-1 | 1.00 | 1.00 | 1.01 | 1.02 | 1.03 |
| 162568499 | aoxB | Alcaligenes sp. T12RB | 1.12 | 1.10 | 1.16 | 1.15 | 1.14 |
| 134299330 | arsB | Desulfotomaculum reducens MI-1 | 0.93 | 0.94 | 0.95 | 0.95 | 0.93 |
| 157168017 | ArsC | uncultured bacterium | 0.60 | 0.92 | 0.00 | 0.64 | 0.96 |
| 53757033 | ArsC | Methylococcus capsulatus str. Bath | 0.00 | 0.93 | 0.94 | 0.99 | 0.99 |
| 196478323 | ArsC | Phenylobacterium zucineum HLK1 | 1.02 | 1.12 | 1.05 | 1.07 | 1.13 |
| 88864142 | ArsC | Jannaschia sp. CCS1 | 0.95 | 1.00 | 0.97 | 0.99 | 1.04 |
| 117618201 | arsB | Aeromonas hydrophila subsp. hydrophila ATCC 7966 | 0.97 | 1.02 | 0.98 | 1.00 | 1.03 |
| 110623058 | arsB | Micrococcus luteus | 0.94 | 1.00 | 0.94 | 1.01 | 1.04 |
| 162568541 | aoxB | uncultured bacterium | 0.93 | 1.00 | 0.96 | 0.99 | 1.02 |
| 119376651 | ArsC | Paracoccus denitrificans PD1222 | 0.97 | 1.00 | 1.00 | 1.04 | 1.03 |
| 184195307 | ArsC | Burkholderia phymatum STM815 | 1.05 | 1.06 | 1.08 | 1.07 | 1.10 |
| 192810313 | ArsC | Geobacillus sp. Y412MC10 | 0.00 | 0.62 | 0.60 | 0.94 | 0.91 |
| 120404199 | ArsC | Mycobacterium vanbaalenii PYR-1 | 0.99 | 1.01 | 0.66 | 1.04 | 1.05 |
| 221157919 | ArsC | Thermomicrobium roseum DSM 5159 | 0.61 | 0.92 | 0.93 | 0.63 | 0.99 |
| 114705878 | ArsC | Fulvimarina pelagi HTCC2506 | 0.62 | 0.99 | 0.97 | 1.00 | 0.96 |
| 152987507 | ArsC | Pseudomonas aeruginosa PA7 | 1.01 | 0.99 | 1.03 | 1.06 | 1.01 |
| 168699430 | arsM | Gemmata obscuriglobus UQM 2246 | 0.94 | 0.67 | 0.66 | 1.05 | 1.09 |
| 258515753 | arsM | Desulfotomaculum acetoxidans DSM 771 | 0.94 | 0.97 | 0.99 | 0.96 | 0.95 |
| 109626263 | ArsA | Haloquadratum walsbyi DSM 16790 | 1.10 | 1.09 | 1.14 | 1.13 | 1.10 |
| 147920037 | arsB | uncultured methanogenic archaeon RC-I | 0.00 | 0.92 | 0.93 | 0.99 | 0.95 |
| 223553646 | ArsC | Tolumonas auensis DSM 9187 | 0.66 | 0.98 | 1.01 | 0.96 | 1.06 |
| 258405077 | arsM | Desulfohalobium retbaense DSM 5692 | 0.61 | 0.96 | 0.89 | 0.96 | 0.97 |
| 189338584 | ArsA | Burkholderia multivorans ATCC 17616 | 0.97 | 1.00 | 0.96 | 0.98 | 1.02 |
| 23014994 | ArsC | Magnetospirillum magnetotacticum MS-1 | 1.08 | 1.10 | 1.12 | 1.14 | 1.10 |
| 219719342 | ArsC | Stenotrophomonas sp. SKA14 | 1.02 | 1.08 | 1.14 | 1.02 | 1.10 |
| 77454729 | ArsC | Rhodococcus erythropolis PR4 | 1.01 | 1.03 | 1.03 | 1.04 | 1.08 |
| 145214735 | arsB | Mycobacterium gilvum PYR-GCK | 1.01 | 1.04 | 1.02 | 1.04 | 1.05 |
| 118036687 | ArsC | Burkholderia phytofirmans PsJN | 0.92 | 1.06 | 1.00 | 1.04 | 1.13 |
| 81295777 | arsB | Streptomyces sp. FR-008 | 0.98 | 0.98 | 0.99 | 1.02 | 0.96 |
| 56314320 | ArsC | Azoarcus sp. EbN1 | 0.99 | 1.01 | 1.00 | 1.02 | 1.06 |
| 209907326 | ArsC | Enterobacter cancerogenus ATCC 35316 | 0.60 | 0.60 | 0.89 | 0.94 | 0.89 |
| 51893507 | arsM | Symbiobacterium thermophilum IAM 14863 | 1.02 | 1.08 | 1.06 | 1.06 | 1.08 |
| 151857 | ArsA |  | 0.00 | 0.89 | 0.87 | 0.63 | 0.96 |
| 77974412 | ArsC | Yersinia frederiksenii ATCC 33641 | 1.03 | 0.71 | 1.01 | 1.05 | 1.02 |
| 94423396 | ArsC | Sphingomonas sp. SKA58 | 1.02 | 1.02 | 1.02 | 1.06 | 1.03 |
| 126627073 | ArsC | Marinobacter sp. ELB17 | 0.91 | 0.93 | 0.89 | 0.95 | 0.97 |
| 11691635 | ArsC | Pseudomonas putida | 1.05 | 1.09 | 1.05 | 1.07 | 1.11 |
| 23015073 | ArsC | Magnetospirillum magnetotacticum MS-1 | 1.07 | 1.11 | 1.08 | 1.08 | 1.12 |
| 253575807 | arsM | Paenibacillus sp. oral taxon 786 str. D14 | 0.67 | 0.97 | 0.94 | 1.01 | 1.01 |
| 119960751 | ArsC | Arthrobacter aurescens TC1 | 0.00 | 0.94 | 0.60 | 0.93 | 0.98 |
| 90420481 | ArsC | Aurantimonas sp. SI85-9A1 | 0.66 | 1.10 | 1.02 | 1.05 | 1.11 |
| 90903265 | aoxB | uncultured bacterium | 0.60 | 0.91 | 0.58 | 0.94 | 0.92 |
| 162568507 | aoxB | Limnobacter sp. 83 | 0.00 | 0.57 | 0.00 | 0.90 | 0.89 |
| 213964454 | ArsC | Corynebacterium amycolatum SK46 | 0.00 | 0.00 | 0.00 | 0.63 | 0.94 |
| 220935414 | ArsC | Thioalkalivibrio sp. HL-EbGR7 | 0.96 | 1.00 | 0.95 | 0.99 | 1.02 |
| 56314660 | ArsC | Azoarcus sp. EbN1 | 0.95 | 0.95 | 0.98 | 1.00 | 0.97 |
| 170026536 | aoxB | Pseudomonas sp. TS44 | 0.00 | 1.01 | 0.62 | 0.97 | 0.00 |
| 93450477 | ArsC | delta proteobacterium MLMS-1 | 1.03 | 1.04 | 1.07 | 1.05 | 1.03 |
| 151583233 | ArsC | Opitutaceae bacterium TAV2 | 0.90 | 0.92 | 0.61 | 0.94 | 0.91 |
| 114228253 | ArsC | Alkalilimnicola ehrlichei MLHE-1 | 0.66 | 0.99 | 0.68 | 0.98 | 1.03 |
| 133740622 | aoxB | Thiomonas sp. 3As | 0.93 | 0.95 | 0.94 | 0.99 | 0.96 |
| 69937812 | ArsC | Paracoccus denitrificans PD1222 | 1.05 | 1.08 | 1.13 | 1.03 | 1.15 |
| 94483091 | arsB | Ochrobactrum tritici | 0.00 | 0.96 | 0.93 | 0.97 | 0.97 |
| 162568551 | aoxB | uncultured bacterium | 0.74 | 0.69 | 0.00 | 0.00 | 0.00 |
| 110632685 | ArsC | Mesorhizobium sp. BNC1 | 0.59 | 0.92 | 0.89 | 0.64 | 0.97 |
| 150956672 | arsB | Klebsiella pneumoniae subsp. pneumoniae MGH 78578 | 0.89 | 0.90 | 0.92 | 0.95 | 0.97 |
| 152971348 | ArsC | Klebsiella pneumoniae subsp. pneumoniae MGH 78578 | 0.63 | 0.94 | 0.00 | 0.96 | 1.00 |
| 162568565 | aoxB | uncultured bacterium | 1.00 | 1.00 | 1.00 | 1.07 | 1.02 |
| 27467253 | arsB | Staphylococcus epidermidis ATCC 12228 | 0.00 | 0.90 | 0.55 | 0.63 | 0.65 |
| 150031719 | ArsC | Sinorhizobium medicae WSM419 | 0.58 | 0.90 | 0.89 | 0.92 | 0.96 |
| 87120210 | aoxB | Marinomonas sp. MED121 | 0.96 | 1.12 | 0.98 | 1.05 | 1.12 |
| 110679646 | ArsC | Roseobacter denitrificans OCh 114 | 0.97 | 0.63 | 0.96 | 1.00 | 0.97 |
| 269306796 | arsM | Xylanimonas cellulosilytica DSM 15894 | 1.06 | 1.07 | 1.08 | 1.15 | 1.11 |
| 38505862 | ArsC | Synechocystis sp. PCC 6803 | 0.95 | 0.61 | 0.91 | 0.96 | 0.92 |
| 114538726 | ArsC | Fulvimarina pelagi HTCC2506 | 0.63 | 0.96 | 0.95 | 0.96 | 0.98 |
| 39935327 | arsB | Rhodopseudomonas palustris CGA009 | 0.88 | 0.60 | 0.59 | 0.93 | 0.62 |
| 194565820 | ArsC | Burkholderia dolosa AUO158 | 0.00 | 0.58 | 0.00 | 0.85 | 0.88 |
| 13473384 | ArsC | Mesorhizobium loti MAFF303099 | 0.63 | 0.93 | 0.94 | 0.95 | 1.00 |
| 111020358 | ArsC | Rhodococcus sp. RHA1 | 0.94 | 1.03 | 0.96 | 1.01 | 1.05 |
| 118467822 | arsB | Mycobacterium smegmatis str. MC2 155 | 0.00 | 1.03 | 0.98 | 0.70 | 1.09 |
| 67542697 | ArsA | Burkholderia vietnamiensis G4 | 0.95 | 0.60 | 0.94 | 0.98 | 0.93 |
| 85835511 | aoxB | Vibrio sp. MED222 | 1.26 | 1.25 | 1.25 | 1.18 | 1.22 |
| 53758066 | ArsC | Methylococcus capsulatus str. Bath | 0.98 | 1.03 | 1.01 | 1.04 | 1.08 |
| 162568509 | aoxB | Pseudomonas sp. 1 | 0.96 | 0.98 | 0.98 | 1.02 | 0.99 |
| 148829422 | ArsC | Erythrobacter sp. SD-21 | 0.99 | 1.09 | 1.05 | 1.05 | 1.09 |
| 57640778 | ArsC | Thermococcus kodakarensis KOD1 | 0.00 | 0.62 | 0.00 | 0.95 | 0.64 |
| 103487310 | ArsC | Sphingopyxis alaskensis RB2256 | 0.97 | 0.91 | 0.98 | 1.00 | 0.97 |
| 15597475 | ArsC | Pseudomonas aeruginosa PAO1 | 0.95 | 0.97 | 0.94 | 0.98 | 0.99 |
| 156719476 | arsB | Hydrogenobaculum sp. Y04AAS1 | 0.00 | 0.93 | 0.00 | 0.98 | 1.04 |
| 27351342 | ArsC | Bradyrhizobium japonicum USDA 110 | 0.65 | 0.99 | 0.63 | 0.67 | 1.06 |
| 145214734 | ArsC | Mycobacterium gilvum PYR-GCK | 1.09 | 1.06 | 1.09 | 1.10 | 1.08 |
| 206567312 | ArsC | Klebsiella pneumoniae 342 | 0.00 | 0.63 | 0.97 | 0.99 | 0.96 |
| 187478323 | ArsC | Bordetella avium 197N | 0.57 | 0.00 | 0.00 | 0.57 | 0.58 |
| 169828489 | ArsC | Lysinibacillus sphaericus C3-41 | 0.62 | 0.62 | 0.59 | 0.94 | 0.00 |
| 30909240 | ArsC | Agrobacterium sp. 5A | 0.97 | 0.98 | 1.01 | 1.03 | 1.00 |
| 110623038 | arsB | Arthrobacter sp. A03 | 0.93 | 0.96 | 0.96 | 1.02 | 1.00 |
| 118465109 | arsB | Mycobacterium avium 104 | 0.95 | 1.04 | 0.99 | 1.01 | 1.06 |
| 283559495 | arsM | Rhodomicrobium vannielii ATCC 17100 | 0.94 | 0.98 | 0.95 | 0.66 | 1.00 |
| 221080481 | ArsC | Variovorax paradoxus S110 | 0.00 | 0.93 | 0.61 | 0.95 | 1.01 |
| 153871382 | aoxB | Beggiatoa sp. PS | 0.00 | 0.58 | 0.00 | 0.00 | 0.57 |
| 89901854 | aoxB | Rhodoferax ferrireducens T118 | 0.61 | 0.95 | 0.63 | 0.98 | 0.95 |
| 162568523 | aoxB | Pseudomonas sp. 46 | 0.58 | 0.58 | 0.00 | 0.91 | 0.95 |
| 269838062 | arsM | Sphaerobacter thermophilus DSM 20745 | 1.00 | 1.00 | 0.98 | 1.01 | 1.02 |
| 220711393 | ArsC | Comamonas testosteroni KF-1 | 0.62 | 0.90 | 0.92 | 0.96 | 0.63 |
| 167461244 | ArsC | Paenibacillus larvae subsp. larvae BRL-230010 | 0.00 | 0.00 | 0.00 | 0.00 | 0.55 |
| 169004518 | ArsC | Paenibacillus sp. JDR-2 | 0.00 | 0.97 | 0.94 | 0.99 | 1.02 |
| 144900614 | ArsC | Magnetospirillum gryphiswaldense MSR-1 | 1.08 | 1.04 | 1.08 | 1.10 | 1.05 |
| 110283534 | ArsC | Mesorhizobium sp. BNC1 | 0.95 | 0.98 | 0.96 | 1.05 | 1.04 |
| 162568575 | aoxB | uncultured bacterium | 0.00 | 0.62 | 0.57 | 0.91 | 0.61 |
| 196201087 | ArsC | Cyanothece sp. PCC 7822 | 0.00 | 0.58 | 0.56 | 0.00 | 0.61 |
| 160346285 | aoxB | Burkholderia multivorans ATCC 17616 | 0.97 | 0.94 | 0.62 | 0.99 | 1.02 |
| 162568559 | aoxB | uncultured bacterium | 0.63 | 1.01 | 1.01 | 1.04 | 1.02 |
| 162568517 | aoxB | Pseudomonas sp. 72 | 0.98 | 1.02 | 1.01 | 1.02 | 1.09 |
| 162568577 | aoxB | uncultured bacterium | 1.03 | 1.01 | 1.05 | 1.07 | 1.04 |
| 78223987 | arsM | Geobacter metallireducens GS-15 | 0.00 | 0.60 | 0.00 | 0.62 | 0.90 |
| 88790999 | ArsA | Nitrococcus mobilis Nb-231 | 0.00 | 0.59 | 0.00 | 0.59 | 0.87 |
| 187720077 | ArsC | Burkholderia phytofirmans PsJN | 0.00 | 0.99 | 0.60 | 0.94 | 1.03 |
| 24983439 | ArsC | Pseudomonas putida KT2440 | 0.59 | 0.92 | 0.88 | 0.94 | 0.90 |
| 112821555 | ArsC | Sphingomonas sp. KA1 | 0.99 | 0.93 | 0.99 | 0.98 | 0.97 |
| 114050077 | arsB | Streptomyces ambofaciens | 1.05 | 1.06 | 1.00 | 1.01 | 1.07 |
| 56421757 | ArsC | Geobacillus kaustophilus HTA426 | 0.89 | 0.91 | 0.94 | 0.97 | 0.94 |
| 15074007 | ArsC | Sinorhizobium meliloti | 0.00 | 0.96 | 0.96 | 1.02 | 0.99 |
| 71741082 | ArsC | Pelobacter propionicus DSM 2379 | 0.00 | 0.95 | 0.62 | 1.02 | 0.98 |
| 148256011 | ArsC | Bradyrhizobium sp. BTAi1 | 0.67 | 1.00 | 0.67 | 0.99 | 1.07 |
| 90903283 | aoxB | uncultured bacterium | 0.93 | 0.93 | 0.97 | 1.00 | 0.96 |
| 189348441 | ArsC | Burkholderia multivorans ATCC 17616 | 0.00 | 0.00 | 0.00 | 0.61 | 0.94 |
| 124549809 | ArsC | Shewanella putrefaciens 200 | 0.00 | 0.90 | 0.00 | 0.60 | 0.93 |
| 114778865 | ArsC | Mariprofundus ferrooxydans PV-1 | 1.05 | 1.03 | 1.09 | 1.11 | 1.08 |
| 183177766 | ArsC | Mycobacterium marinum M | 1.05 | 1.01 | 1.07 | 1.07 | 1.03 |
| 189354105 | ArsC | Burkholderia multivorans ATCC 17616 | 0.00 | 0.62 | 0.00 | 0.94 | 0.93 |
| 118571830 | ArsC | Mycobacterium ulcerans Agy99 | 1.05 | 1.04 | 1.09 | 1.09 | 1.07 |
| 158333063 | ArsC | Azorhizobium caulinodans ORS 571 | 1.19 | 1.21 | 1.22 | 1.18 | 1.26 |
| 217501675 | ArsC | Methylocella silvestris BL2 | 0.56 | 0.59 | 0.59 | 0.92 | 0.92 |
| 77814270 | ArsC | Shewanella putrefaciens CN-32 | 1.08 | 1.20 | 1.16 | 1.16 | 1.23 |
| 114569204 | ArsC | Maricaulis maris MCS10 | 1.20 | 1.29 | 1.23 | 1.24 | 1.31 |
| 166085368 | ArsC | Microcystis aeruginosa NIES-843 | 0.92 | 0.93 | 0.94 | 0.96 | 0.94 |
| 123443563 | ArsC | Yersinia enterocolitica subsp. enterocolitica 8081 | 0.93 | 0.94 | 0.98 | 0.99 | 0.92 |
| 84498490 | ArsC | Janibacter sp. HTCC2649 | 0.00 | 0.95 | 0.00 | 0.96 | 0.98 |
| 162568549 | aoxB | uncultured bacterium | 1.07 | 1.14 | 1.12 | 1.16 | 1.13 |
| 84362891 | ArsC | Burkholderia dolosa AUO158 | 0.92 | 0.88 | 0.91 | 0.98 | 0.96 |
| 122090525 | arsB | Yersinia enterocolitica subsp. enterocolitica 8081 | 0.90 | 1.02 | 0.96 | 1.01 | 0.99 |
| 194456300 | ArsC | Salmonella enterica subsp. enterica serovar Kentucky str. CVM29188 | 0.00 | 0.97 | 0.64 | 0.96 | 1.00 |
| 198252074 | ArsC | Octadecabacter antarcticus 307 | 1.05 | 1.00 | 0.99 | 1.04 | 1.01 |
| 123441473 | ArsC | Yersinia enterocolitica subsp. enterocolitica 8081 | 0.00 | 0.00 | 0.00 | 0.00 | 0.93 |
| 1653494 | ArsC | Synechocystis sp. PCC 6803 | 0.63 | 0.95 | 0.96 | 1.03 | 0.99 |
| 110637991 | ArsC | Cytophaga hutchinsonii ATCC 33406 | 0.00 | 0.59 | 0.58 | 0.59 | 0.89 |
| 68190298 | ArsC | Mesorhizobium sp. BNC1 | 0.59 | 0.61 | 0.61 | 0.96 | 0.93 |
| 119898644 | ArsC | Azoarcus sp. BH72 | 1.16 | 1.13 | 1.19 | 1.17 | 1.15 |
| 42738187 | ArsC | Bacillus cereus ATCC 10987 | 0.00 | 0.60 | 0.00 | 0.63 | 0.62 |
| 60686970 | ArsA | Acidithiobacillus caldus | 1.01 | 1.03 | 1.07 | 1.07 | 1.03 |
| 126434044 | arsB | Mycobacterium sp. JLS | 0.00 | 0.00 | 0.00 | 0.58 | 0.00 |
| 110623060 | arsB | Microbacterium sp. A33 | 0.98 | 1.05 | 1.02 | 1.04 | 1.10 |
| 126319653 | ArsC | Pseudomonas putida GB-1 | 1.02 | 1.00 | 1.03 | 1.06 | 1.05 |
| 124894014 | ArsC | Burkholderia dolosa AUO158 | 0.98 | 0.99 | 0.98 | 1.00 | 1.00 |
| 118029486 | ArsC | Burkholderia phymatum STM815 | 1.02 | 1.11 | 1.06 | 1.07 | 1.12 |
| 116668780 | arsB | Arthrobacter sp. FB24 | 0.57 | 0.88 | 0.92 | 0.93 | 0.88 |
| 119943731 | ArsC | Comamonas sp. CNB-1 | 0.96 | 0.96 | 0.97 | 1.02 | 1.02 |
| 114326852 | ArsC | Granulibacter bethesdensis CGDNIH1 | 0.00 | 0.98 | 0.62 | 0.64 | 0.98 |
| 162568501 | aoxB | Alcaligenes sp. YI13H | 0.92 | 0.91 | 0.90 | 0.93 | 0.93 |
| 52348376 | ArsC | Bacillus licheniformis DSM 13 | 0.00 | 0.59 | 0.00 | 0.88 | 0.87 |
| 198264086 | ArsC | Octadecabacter antarcticus 238 | 0.96 | 1.00 | 0.93 | 0.99 | 1.04 |
| 94972235 | ArsC | Deinococcus geothermalis DSM 11300 | 0.68 | 1.01 | 1.00 | 1.00 | 1.04 |
| 118720167 | ArsC | Burkholderia multivorans ATCC 17616 | 1.11 | 1.11 | 1.14 | 1.12 | 1.13 |
| 54019342 | arsB | Nocardia farcinica IFM 10152 | 0.86 | 0.61 | 0.58 | 0.92 | 0.97 |
| 189338545 | ArsC | Burkholderia multivorans ATCC 17616 | 1.03 | 1.07 | 1.07 | 1.07 | 1.09 |
| 163668072 | aoxB | Chloroflexus aurantiacus J-10-fl | 0.99 | 1.03 | 1.01 | 1.02 | 1.01 |
| 84691528 | ArsC | Parvularcula bermudensis HTCC2503 | 0.94 | 0.97 | 0.96 | 1.01 | 1.05 |
| 206566527 | ArsC | Klebsiella pneumoniae 342 | 0.00 | 0.98 | 0.99 | 0.67 | 1.00 |
| 254430241 | arsM | Cyanobium sp. PCC 7001 | 0.88 | 0.67 | 0.96 | 0.65 | 0.66 |
| 224589200 | aoxB | Hydrogenobacter sp. GV8-4AC-C1 | 0.98 | 0.99 | 0.67 | 0.00 | 0.65 |
| 190574362 | ArsC | Stenotrophomonas maltophilia K279a | 1.17 | 1.19 | 1.22 | 1.15 | 1.23 |
| 144944757 | ArsC | Geobacter bemidjiensis Bem | 0.98 | 0.98 | 0.68 | 0.70 | 0.68 |
| 221068205 | ArsC | Comamonas testosteroni KF-1 | 0.00 | 0.93 | 0.00 | 0.62 | 0.94 |
| 168805058 | aoxB | Thiomonas sp. WJ68 | 1.00 | 1.01 | 1.01 | 1.03 | 1.02 |
| 86572122 | ArsC | Rhodopseudomonas palustris HaA2 | 0.00 | 0.58 | 0.00 | 0.90 | 0.89 |
| 163858870 | ArsC | Bordetella petrii DSM 12804 | 0.61 | 0.93 | 0.94 | 0.96 | 0.95 |
| 57233796 | arsM | Dehalococcoides ethenogenes 195 | 0.63 | 0.94 | 0.91 | 0.93 | 0.62 |
| 162568489 | aoxB | Thiomonas arsenivorans | 1.06 | 1.04 | 1.05 | 1.11 | 1.08 |
| 214039575 | ArsC | marine gamma proteobacterium HTCC2148 | 0.65 | 0.93 | 0.00 | 0.63 | 0.62 |
| 103488017 | ArsC | Sphingopyxis alaskensis RB2256 | 0.98 | 0.95 | 0.98 | 1.04 | 1.02 |
| 94984863 | ArsC | Deinococcus geothermalis DSM 11300 | 1.03 | 0.98 | 1.01 | 1.04 | 1.02 |
| 117993070 | ArsC | Burkholderia phytofirmans PsJN | 1.03 | 1.10 | 1.08 | 1.06 | 1.12 |
| 148256857 | ArsC | Bradyrhizobium sp. BTAi1 | 0.61 | 0.93 | 0.92 | 0.97 | 0.96 |
| 198262961 | ArsC | gamma proteobacterium HTCC5015 | 0.92 | 0.98 | 0.91 | 0.96 | 1.00 |
| 126667582 | ArsC | Marinobacter sp. ELB17 | 0.57 | 0.60 | 0.91 | 0.62 | 0.90 |
| 90903271 | aoxB | uncultured bacterium | 0.90 | 0.89 | 0.92 | 0.94 | 0.92 |
| 220933438 | arsM | Thioalkalivibrio sp. HL-EbGR7 | 0.96 | 1.00 | 0.96 | 1.00 | 1.04 |
| 223509045 | ArsC | Ricinus communis | 1.05 | 1.01 | 1.06 | 1.08 | 1.06 |
| 51451372 | arsB | Pseudomonas stutzeri | 1.07 | 1.04 | 1.07 | 1.09 | 1.04 |
| 142850418 | ArsC | Aeromonas salmonicida subsp. salmonicida A449 | 0.00 | 0.65 | 0.63 | 1.02 | 0.97 |
| 158320129 | arsM | Alkaliphilus oremlandii OhILAs | 0.62 | 0.96 | 0.59 | 0.90 | 0.98 |
| 32446496 | ArsA | Rhodopirellula baltica SH 1 | 0.58 | 0.93 | 0.93 | 0.96 | 0.65 |
| 70996440 | ArsC | Aspergillus fumigatus Af293 | 0.00 | 0.89 | 0.00 | 0.00 | 0.92 |
| 194467376 | ArsC | Lactobacillus reuteri 100-23 | 0.00 | 0.99 | 0.60 | 0.95 | 1.02 |
| 46203957 | ArsC | Magnetospirillum magnetotacticum MS-1 | 0.98 | 1.06 | 1.03 | 1.01 | 1.08 |
| 162568571 | aoxB | uncultured bacterium | 0.62 | 0.62 | 0.62 | 0.97 | 0.96 |
| 157679856 | arsB | Bacillus pumilus SAFR-032 | 0.00 | 0.87 | 0.63 | 0.89 | 0.90 |
| 163795599 | ArsC | alpha proteobacterium BAL199 | 1.01 | 0.99 | 1.00 | 1.04 | 1.02 |
| 98977961 | ArsC | Sphingopyxis alaskensis RB2256 | 1.09 | 1.06 | 1.09 | 1.10 | 1.09 |
| 88062822 | ArsC | Rhizobium leguminosarum | 1.06 | 1.05 | 1.07 | 1.04 | 1.03 |
| 94972016 | ArsC | Deinococcus geothermalis DSM 11300 | 0.00 | 0.97 | 0.00 | 0.65 | 0.68 |
| 47572214 | ArsC | Rubrivivax gelatinosus PM1 | 0.00 | 1.03 | 0.98 | 1.02 | 1.06 |
| 11691634 | arsB | Pseudomonas putida | 0.00 | 0.59 | 0.00 | 0.59 | 0.62 |
| 39998044 | ArsC | Geobacter sulfurreducens PCA | 0.00 | 0.92 | 0.59 | 0.00 | 0.90 |
| 142852379 | ArsC | Aeromonas salmonicida subsp. salmonicida A449 | 1.12 | 1.08 | 1.10 | 1.10 | 1.10 |
| 219847326 | aoxB | Chloroflexus aggregans DSM 9485 | 1.02 | 1.05 | 1.01 | 1.03 | 1.08 |
| 145216540 | arsB | Mycobacterium gilvum PYR-GCK | 0.60 | 0.91 | 0.00 | 0.93 | 0.95 |
| 99080367 | ArsC | Silicibacter sp. TM1040 | 1.00 | 1.05 | 1.03 | 1.03 | 1.12 |
| 255008414 | arsM | Bacteroides fragilis 3_1_12 | 0.00 | 0.62 | 0.65 | 0.00 | 0.93 |
| 6460527 | ArsC | Deinococcus radiodurans R1 | 0.95 | 0.95 | 0.94 | 1.01 | 0.98 |
| 150006666 | ArsC | Bacteroides vulgatus ATCC 8482 | 0.68 | 1.02 | 0.96 | 1.01 | 1.07 |
| 157371755 | ArsC | Serratia proteamaculans 568 | 0.59 | 0.61 | 0.61 | 0.93 | 0.60 |
| 77820294 | arsB | Pseudomonas putida | 0.00 | 1.01 | 0.94 | 1.00 | 1.02 |
| 84498489 | arsB | Janibacter sp. HTCC2649 | 0.98 | 1.00 | 0.98 | 0.99 | 1.01 |
| 197787179 | ArsC | Oligotropha carboxidovorans OM5 | 0.00 | 0.00 | 0.00 | 0.60 | 0.87 |
| 77655372 | ArsC | Rhodopseudomonas palustris BisB5 | 1.04 | 1.09 | 1.07 | 1.07 | 1.13 |
| 124266776 | ArsC | Methylibium petroleiphilum PM1 | 0.99 | 0.99 | 0.99 | 1.02 | 1.01 |
| 89473606 | aoxB | arsenite-oxidising alpha proteobacterium NT-4 | 0.61 | 0.64 | 0.58 | 0.64 | 0.62 |
| 95111341 | arsB | Pseudomonas | 0.00 | 0.60 | 0.00 | 0.63 | 0.92 |
| 162283897 | ArsC | Hoeflea phototrophica DFL-43 | 0.00 | 0.00 | 0.00 | 0.00 | 0.91 |
| 151421640 | arsB | Nitratiruptor sp. SB155-2 | 0.00 | 0.95 | 0.97 | 1.05 | 0.99 |
| 161389958 | ArsC | Phaeobacter gallaeciensis BS107 | 1.07 | 1.06 | 1.06 | 1.06 | 1.13 |
| 78059683 | ArsC | Burkholderia sp. 383 | 0.00 | 0.00 | 0.00 | 0.00 | 0.60 |
| 221636204 | ArsC | Thermomicrobium roseum DSM 5159 | 0.93 | 0.59 | 0.89 | 0.96 | 0.93 |
| 103487884 | ArsC | Sphingopyxis alaskensis RB2256 | 0.96 | 0.94 | 0.98 | 1.01 | 0.96 |
| 110623050 | arsB | Rhodococcus sp. B03 | 0.63 | 1.06 | 1.03 | 1.01 | 1.05 |
| 99038918 | ArsC | Silicibacter sp. TM1040 | 0.00 | 0.61 | 0.00 | 0.61 | 0.98 |
| 209500157 | ArsC | Burkholderia sp. H160 | 0.00 | 0.95 | 0.90 | 0.96 | 0.98 |
| 21226763 | arsM | Methanosarcina mazei Go1 | 0.00 | 0.00 | 0.00 | 0.58 | 0.93 |
| 90201441 | ArsC | Mycobacterium vanbaalenii PYR-1 | 1.01 | 1.01 | 1.02 | 1.06 | 1.04 |
| 104782355 | ArsC | Pseudomonas entomophila L48 | 1.07 | 1.09 | 1.12 | 1.13 | 1.12 |
| 219869487 | arsM | Desulfovibrio desulfuricans subsp. desulfuricans str. ATCC 27774 | 1.04 | 1.07 | 1.07 | 0.71 | 1.04 |
| 51855508 | ArsA | Symbiobacterium thermophilum IAM 14863 | 0.98 | 0.93 | 0.97 | 0.99 | 0.95 |
| 119863435 | ArsC | Psychromonas ingrahamii 37 | 0.00 | 0.00 | 0.00 | 0.00 | 0.86 |
| 27353547 | arsB | Bradyrhizobium japonicum USDA 110 | 0.96 | 1.00 | 0.97 | 0.96 | 0.99 |
| 162568525 | aoxB | Aminobacter sp. 86 | 0.95 | 0.95 | 0.96 | 0.67 | 1.01 |
| 116619429 | ArsC | Solibacter usitatus Ellin6076 | 0.89 | 0.59 | 0.89 | 0.91 | 0.92 |
| 118003223 | ArsC | Comamonas testosteroni KF-1 | 0.63 | 0.95 | 0.97 | 1.00 | 0.96 |
| 188028249 | ArsC | Erwinia tasmaniensis Et1/99 | 0.60 | 0.95 | 0.90 | 0.95 | 0.99 |
| 77454726 | arsB | Rhodococcus erythropolis PR4 | 0.00 | 0.59 | 0.00 | 0.60 | 0.59 |
| 269792113 | arsM | Thermanaerovibrio acidaminovorans DSM 6589 | 0.99 | 1.02 | 1.01 | 1.06 | 1.02 |
| 77390477 | ArsC | Rhodobacter sphaeroides 2.4.1 | 0.00 | 0.98 | 0.97 | 0.67 | 1.06 |
| 117610002 | ArsC | Magnetococcus sp. MC-1 | 0.00 | 0.59 | 0.00 | 0.00 | 0.00 |
| 156527925 | ArsC | Vibrio harveyi ATCC BAA-1116 | 0.00 | 0.59 | 0.00 | 0.00 | 0.59 |
| 162568581 | aoxB | uncultured bacterium | 1.02 | 1.03 | 1.07 | 1.07 | 1.05 |
| 91793002 | ArsC | Shewanella denitrificans OS217 | 0.87 | 1.02 | 0.98 | 0.96 | 1.06 |
| 116623500 | arsM | Solibacter usitatus Ellin6076 | 0.98 | 0.67 | 1.01 | 1.00 | 0.98 |
| 167583695 | ArsC | Burkholderia ubonensis Bu | 0.00 | 0.93 | 0.00 | 0.63 | 0.93 |
| 257456586 | arsM | Treponema vincentii ATCC 35580 | 0.92 | 1.05 | 1.00 | 1.02 | 1.07 |
| 221179348 | ArsC | Burkholderia multivorans CGD2M | 0.00 | 0.57 | 0.87 | 0.88 | 0.86 |
| 66850793 | ArsC | Aspergillus fumigatus Af293 | 1.10 | 1.19 | 1.15 | 1.15 | 1.20 |
| 30909238 | ArsC | Agrobacterium sp. 5B | 0.00 | 0.00 | 0.86 | 0.60 | 0.58 |
| 90903253 | aoxB | uncultured bacterium | 0.00 | 0.89 | 0.00 | 0.90 | 0.91 |
| 84393286 | aoxB | Vibrio splendidus 12B01 | 0.00 | 0.90 | 0.00 | 0.61 | 0.96 |
| 15156573 | ArsC | Agrobacterium tumefaciens str. C58 | 0.00 | 0.00 | 0.00 | 0.56 | 0.84 |
| 133740356 | arsB | Herminiimonas arsenicoxydans | 0.00 | 0.61 | 0.91 | 0.94 | 0.89 |
| 54027836 | ArsC | Nocardia farcinica IFM 10152 | 1.05 | 1.09 | 1.08 | 1.09 | 1.09 |
| 157406148 | ArsC | Methylobacterium populi BJ001 | 1.05 | 1.11 | 1.07 | 1.06 | 1.14 |
| 146280632 | ArsC | Pseudomonas stutzeri A1501 | 0.59 | 0.59 | 0.87 | 0.88 | 0.59 |
| 121605481 | ArsC | Polaromonas naphthalenivorans CJ2 | 1.19 | 1.21 | 1.23 | 1.23 | 1.21 |
| 160362624 | ArsC | Delftia acidovorans SPH-1 | 1.03 | 1.08 | 1.07 | 1.04 | 1.13 |
| 124899480 | ArsC | Burkholderia dolosa AUO158 | 0.00 | 0.60 | 0.00 | 0.89 | 0.92 |
| 211959444 | arsM | Pseudovibrio sp. JE062 | 0.64 | 0.98 | 0.93 | 0.95 | 1.02 |
| 194562390 | ArsC | Burkholderia dolosa AUO158 | 0.92 | 0.96 | 0.95 | 0.98 | 1.03 |
| 197787378 | ArsC | Oligotropha carboxidovorans OM5 | 0.88 | 0.90 | 0.91 | 0.93 | 0.97 |
| 218707121 | ArsA | Escherichia coli UMN026 | 0.62 | 0.97 | 0.95 | 0.65 | 1.04 |
| 74317446 | arsM | Thiobacillus denitrificans ATCC 25259 | 0.97 | 1.03 | 1.02 | 1.00 | 1.04 |
| 119951727 | ArsC | Arthrobacter aurescens TC1 | 0.98 | 0.63 | 0.95 | 1.01 | 0.98 |
| 115423206 | arsB | Bordetella avium 197N | 0.97 | 0.95 | 0.95 | 0.99 | 0.96 |
| 90903269 | aoxB | uncultured bacterium | 0.60 | 0.93 | 0.00 | 0.93 | 0.95 |
| 209966260 | ArsC | Rhodospirillum centenum SW | 1.03 | 1.00 | 1.05 | 1.06 | 1.04 |
| 162568503 | aoxB | Burkholderia sp. YI019A | 0.66 | 1.01 | 1.00 | 1.04 | 0.00 |
| 168992628 | ArsC | Lysinibacillus sphaericus C3-41 | 0.00 | 0.59 | 0.00 | 0.00 | 0.00 |
| 119958552 | ArsC | Mycobacterium vanbaalenii PYR-1 | 0.00 | 0.60 | 0.00 | 0.00 | 0.63 |
| 118172732 | ArsC | Mycobacterium smegmatis str. MC2 155 | 0.61 | 0.95 | 0.60 | 0.98 | 0.98 |
| 39935326 | ArsC | Rhodopseudomonas palustris CGA009 | 0.72 | 1.05 | 0.69 | 1.01 | 1.04 |
| 146743238 | ArsC | Leptospirillum ferriphilum | 0.00 | 0.60 | 0.00 | 0.00 | 0.92 |
| 7769068 | ArsC | Acidithiobacillus ferrooxidans | 0.00 | 0.60 | 0.61 | 0.98 | 0.65 |
| 117929001 | ArsC | Acidothermus cellulolyticus 11B | 1.02 | 0.67 | 1.04 | 1.06 | 1.04 |
| 154493428 | ArsC | Parabacteroides merdae ATCC 43184 | 0.00 | 0.60 | 0.61 | 0.95 | 0.62 |
| 218291273 | ArsC | Alicyclobacillus acidocaldarius LAA1 | 1.04 | 1.05 | 1.06 | 1.09 | 1.08 |
| 121530504 | ArsC | Ralstonia pickettii 12J | 0.59 | 0.58 | 0.58 | 0.95 | 0.94 |
| 62289913 | ArsC | Brucella abortus biovar 1 str. 9-941 | 0.63 | 0.99 | 0.96 | 1.03 | 1.01 |
| 118436437 | ArsC | Stappia aggregata IAM 12614 | 0.00 | 0.59 | 0.00 | 0.63 | 0.86 |
| 189338852 | ArsC | Burkholderia multivorans ATCC 17616 | 1.15 | 1.10 | 1.12 | 1.13 | 1.13 |
| 110283124 | ArsC | Mesorhizobium sp. BNC1 | 0.00 | 0.62 | 0.61 | 0.95 | 0.96 |
| 146308011 | ArsC | Pseudomonas mendocina ymp | 0.62 | 0.95 | 0.61 | 0.98 | 1.01 |
| 55978310 | aoxB | Thermus thermophilus HB8 | 0.00 | 0.63 | 0.00 | 0.60 | 0.95 |
| 257797304 | arsM | Desulfohalobium retbaense DSM 5692 | 1.15 | 1.16 | 1.18 | 1.17 | 1.16 |
| 98978094 | ArsC | Sphingopyxis alaskensis RB2256 | 0.99 | 0.99 | 1.00 | 1.04 | 1.04 |
| 162568593 | aoxB | uncultured bacterium | 0.00 | 0.62 | 0.59 | 0.95 | 0.97 |
| 89896894 | arsM | Desulfitobacterium hafniense Y51 | 0.59 | 0.90 | 0.92 | 0.92 | 0.86 |
| 110623046 | arsB | Paracoccus sp. A10 | 1.10 | 1.08 | 1.11 | 1.10 | 1.07 |
| 116063246 | aoxB | Aeropyrum pernix K1 | 1.08 | 1.12 | 1.11 | 1.11 | 1.10 |
| 120607444 | ArsC | Acidovorax sp. JS42 | 0.98 | 0.99 | 0.99 | 1.02 | 1.00 |
| 119717967 | ArsC | Nocardioides sp. JS614 | 1.06 | 1.09 | 1.08 | 1.12 | 1.16 |
| 162568497 | aoxB | Leptothrix sp. S1-1 | 0.00 | 1.00 | 0.64 | 0.98 | 1.02 |
| 134095158 | ArsC | Herminiimonas arsenicoxydans | 0.94 | 0.63 | 0.94 | 0.96 | 0.65 |
| 119458982 | ArsC | marine gamma proteobacterium HTCC2080 | 0.00 | 0.58 | 0.58 | 0.92 | 0.62 |
| 162568561 | aoxB | uncultured bacterium | 0.93 | 0.95 | 0.92 | 0.97 | 0.97 |
| 19888411 | ArsA | Methanopyrus kandleri AV19 | 0.96 | 0.94 | 1.00 | 1.02 | 0.97 |
| 119957016 | ArsC | Mycobacterium vanbaalenii PYR-1 | 1.06 | 0.71 | 0.70 | 0.70 | 0.00 |
| 159883182 | ArsC | Hydrogenivirga sp. 128-5-R1-1 | 0.00 | 0.57 | 0.00 | 0.94 | 0.88 |
| 39650472 | ArsC | Rhodopseudomonas palustris CGA009 | 1.08 | 1.05 | 1.09 | 1.10 | 1.08 |
| 145222248 | ArsC | Mycobacterium gilvum PYR-GCK | 1.07 | 1.03 | 0.71 | 1.07 | 1.05 |
| 189338579 | ArsC | Burkholderia multivorans ATCC 17616 | 0.97 | 0.63 | 0.94 | 1.00 | 0.97 |
| 125624073 | ArsA | Lactococcus lactis subsp. cremoris MG1363 | 0.56 | 0.59 | 0.89 | 0.92 | 0.89 |
| 149175462 | ArsC | Planctomyces maris DSM 8797 | 0.00 | 0.85 | 0.58 | 0.88 | 0.00 |
| 220906104 | ArsC | Cyanothece sp. PCC 7425 | 0.00 | 0.00 | 0.00 | 0.00 | 0.60 |
| 223693967 | ArsC | Desulfobacterium autotrophicum HRM2 | 1.17 | 1.14 | 1.19 | 0.75 | 0.00 |
| 147676775 | arsM | Pelotomaculum thermopropionicum SI | 1.05 | 1.02 | 1.06 | 1.06 | 1.04 |
| 219547917 | ArsC | Vibrio parahaemolyticus 16 | 0.00 | 0.62 | 0.00 | 0.94 | 0.90 |
| 116050225 | arsB | Pseudomonas aeruginosa UCBPP-PA14 | 1.10 | 1.11 | 1.15 | 1.14 | 1.14 |
| 119374770 | ArsC | Paracoccus denitrificans PD1222 | 1.04 | 1.07 | 1.06 | 1.08 | 1.09 |
| 51245731 | ArsC | Desulfotalea psychrophila LSv54 | 0.93 | 1.02 | 0.97 | 1.04 | 1.03 |
| 27464269 | ArsC | Enterobacter cloacae | 0.00 | 0.64 | 0.00 | 0.00 | 0.96 |
| 188583265 | ArsC | Methylobacterium populi BJ001 | 1.10 | 1.13 | 1.14 | 1.14 | 1.13 |
| 187477094 | ArsC | Bordetella avium 197N | 1.02 | 0.67 | 1.01 | 0.67 | 0.97 |
| 118655574 | ArsC | Burkholderia multivorans ATCC 17616 | 1.06 | 1.01 | 1.04 | 1.06 | 1.05 |
| 56314322 | ArsA | Azoarcus sp. EbN1 | 0.89 | 0.90 | 0.88 | 0.95 | 0.94 |
| 77740782 | ArsC | Rhodopseudomonas palustris BisA53 | 0.00 | 0.90 | 0.00 | 0.93 | 0.95 |
| 75676104 | ArsC | Nitrobacter winogradskyi Nb-255 | 1.26 | 1.28 | 1.31 | 1.23 | 1.25 |
| 77019732 | ArsA | Rhodococcus erythropolis PR4 | 1.00 | 1.06 | 1.01 | 1.05 | 1.12 |
| 33469597 | aoxB | Alcaligenes faecalis | 0.99 | 1.04 | 0.68 | 0.00 | 1.01 |
| 170144172 | ArsC | Burkholderia graminis C4D1M | 1.14 | 1.17 | 1.16 | 1.13 | 1.17 |
| 90903261 | aoxB | uncultured bacterium | 1.07 | 1.06 | 1.08 | 1.07 | 1.07 |
| 119949109 | ArsC | Arthrobacter aurescens TC1 | 1.01 | 0.99 | 1.02 | 1.01 | 0.97 |
| 158312423 | ArsC | Frankia sp. EAN1pec | 1.07 | 1.11 | 1.09 | 1.06 | 1.09 |
| 110347011 | ArsC | Mesorhizobium sp. BNC1 | 0.97 | 0.97 | 0.97 | 0.99 | 1.06 |
| 87247613 | aoxB | Mesorhizobium sp. DM1 | 1.09 | 1.04 | 1.09 | 1.07 | 1.13 |
| 170690923 | ArsC | Burkholderia graminis C4D1M | 0.00 | 0.91 | 0.59 | 0.96 | 0.94 |
| 197790853 | ArsC | Oligotropha carboxidovorans OM5 | 0.00 | 0.92 | 0.00 | 0.63 | 0.96 |
| 119353652 | ArsC | Chlorobium phaeobacteroides DSM 266 | 0.91 | 0.95 | 0.62 | 0.98 | 1.01 |
| 73541215 | ArsC | Ralstonia eutropha JMP134 | 0.94 | 1.07 | 0.98 | 1.01 | 1.11 |
| 196185118 | ArsC | Brevundimonas sp. BAL3 | 0.97 | 0.64 | 0.99 | 1.02 | 0.98 |
| 196477735 | ArsC | Phenylobacterium zucineum HLK1 | 0.00 | 0.94 | 0.95 | 0.95 | 0.94 |
| 85058228 | ArsC | Sodalis glossinidius str. 'morsitans' | 0.94 | 1.12 | 1.02 | 1.03 | 1.12 |
| 254510744 | arsM | Rhodobacteraceae bacterium KLH11 | 0.59 | 0.60 | 0.88 | 0.64 | 0.93 |
| 85696951 | ArsC | Nitrobacter sp. Nb-311A | 0.63 | 0.95 | 0.00 | 0.98 | 1.00 |
| 148272257 | arsB | Clavibacter michiganensis subsp. michiganensis NCPPB 382 | 0.96 | 0.95 | 0.96 | 0.99 | 0.96 |
| 150958270 | ArsA | Klebsiella pneumoniae subsp. pneumoniae MGH 78578 | 0.59 | 0.93 | 0.92 | 0.95 | 0.97 |
| 69934124 | ArsC | Paracoccus denitrificans PD1222 | 0.92 | 0.99 | 0.96 | 0.97 | 0.99 |
| 126233845 | ArsC | Mycobacterium sp. JLS | 0.99 | 1.04 | 1.00 | 1.07 | 1.09 |
| 119775049 | ArsC | Shewanella amazonensis SB2B | 0.00 | 0.65 | 0.62 | 0.95 | 0.91 |
| 167359267 | ArsC | Rhizobium leguminosarum bv. trifolii WSM1325 | 0.62 | 0.98 | 0.98 | 1.03 | 1.00 |
| 146743239 | arsB | Leptospirillum ferriphilum | 0.65 | 0.93 | 0.64 | 0.93 | 1.01 |
| 158329784 | ArsC | Azorhizobium caulinodans ORS 571 | 0.61 | 0.98 | 0.96 | 0.65 | 0.95 |
| 161787988 | ArsC | Gluconacetobacter diazotrophicus PAl 5 | 1.05 | 1.04 | 0.72 | 1.07 | 1.06 |
| 119671055 | ArsC | Azoarcus sp. BH72 | 1.08 | 1.13 | 1.14 | 1.09 | 1.11 |
| 162568543 | aoxB | uncultured bacterium | 0.99 | 1.04 | 1.02 | 1.01 | 1.05 |
| 169828140 | ArsC | Lysinibacillus sphaericus C3-41 | 0.00 | 0.00 | 0.00 | 0.00 | 0.87 |
| 162568583 | aoxB | uncultured bacterium | 0.99 | 1.02 | 1.01 | 0.68 | 1.03 |
| 146283160 | ArsC | Pseudomonas stutzeri A1501 | 0.63 | 0.96 | 0.94 | 1.00 | 1.00 |
| 157168013 | ArsC | uncultured bacterium | 0.97 | 1.00 | 1.00 | 1.02 | 1.01 |
| 57225463 | ArsC | Dehalococcoides ethenogenes 195 | 0.94 | 0.98 | 0.99 | 0.98 | 0.96 |
| 13422880 | ArsC | Caulobacter crescentus CB15 | 0.67 | 1.02 | 1.03 | 1.08 | 1.07 |
| 28854202 | arsB | Pseudomonas syringae pv. tomato str. DC3000 | 1.02 | 1.06 | 1.07 | 1.09 | 1.07 |
| 110623040 | arsB | Arthrobacter sp. B02 | 0.00 | 0.59 | 0.00 | 0.60 | 0.94 |
| 73660393 | arsB | Dehalococcoides sp. CBDB1 | 1.01 | 0.99 | 1.01 | 1.00 | 0.98 |
| 163262598 | ArsC | Bordetella petrii | 0.93 | 1.01 | 0.95 | 1.03 | 1.02 |
| 116668784 | ArsC | Arthrobacter sp. FB24 | 0.96 | 1.01 | 0.97 | 1.00 | 1.04 |
| 167728823 | ArsA | Halobacterium salinarum R1 | 1.05 | 1.00 | 1.07 | 1.09 | 1.07 |
| 124260305 | ArsC | Methylibium petroleiphilum PM1 | 1.01 | 0.96 | 1.00 | 1.04 | 1.01 |
| 54019345 | ArsC | Nocardia farcinica IFM 10152 | 1.04 | 1.06 | 1.04 | 0.71 | 1.09 |
| 118667595 | ArsC | Delftia acidovorans SPH-1 | 0.95 | 0.64 | 0.98 | 0.99 | 0.95 |
| 20521522 | arsB | Salmonella typhimurium | 1.11 | 1.09 | 1.15 | 1.15 | 1.12 |
| 56180234 | ArsC | Idiomarina loihiensis L2TR | 0.93 | 1.03 | 0.98 | 1.03 | 1.05 |
| 86282246 | ArsC | Rhizobium etli CFN 42 | 0.00 | 0.96 | 0.65 | 0.99 | 1.02 |
| 91692446 | ArsC | Burkholderia xenovorans LB400 | 0.97 | 0.67 | 1.03 | 1.06 | 1.02 |
| 196113910 | ArsC | Alteromonas macleodii 'Deep ecotype' | 0.00 | 0.57 | 0.00 | 0.57 | 0.87 |
| 167574595 | aoxB | Burkholderia oklahomensis C6786 | 0.74 | 0.79 | 0.76 | 0.00 | 0.00 |
| 110667012 | ArsA | Haloquadratum walsbyi DSM 16790 | 0.00 | 0.00 | 0.00 | 0.93 | 0.00 |
| 110822150 | ArsC | Rhodococcus sp. RHA1 | 0.00 | 0.60 | 0.00 | 0.62 | 0.99 |
| 115422909 | ArsC | Bordetella avium 197N | 0.58 | 0.00 | 0.00 | 0.90 | 0.59 |
| 51855507 | ArsA | Symbiobacterium thermophilum IAM 14863 | 0.00 | 0.00 | 0.00 | 0.00 | 0.64 |
| 154496549 | ArsC | Bacteroides capillosus ATCC 29799 | 0.95 | 0.65 | 0.99 | 1.02 | 0.97 |
| 114799478 | ArsC | Hyphomonas neptunium ATCC 15444 | 0.93 | 0.63 | 0.96 | 0.98 | 0.95 |
| 213155619 | ArsC | Acinetobacter baumannii AB0057 | 0.00 | 0.00 | 0.00 | 0.56 | 0.00 |
| 162568557 | aoxB | uncultured bacterium | 0.00 | 0.64 | 0.00 | 1.00 | 1.04 |
| 110637918 | arsM | Cytophaga hutchinsonii ATCC 33406 | 0.00 | 0.00 | 0.00 | 0.55 | 0.00 |
| 27378197 | ArsC | Bradyrhizobium japonicum USDA 110 | 0.91 | 0.00 | 0.89 | 0.96 | 0.91 |
| 69279982 | ArsC | Mesorhizobium sp. BNC1 | 0.58 | 0.00 | 0.89 | 0.92 | 0.91 |
| 162568563 | aoxB | uncultured bacterium | 0.57 | 0.00 | 0.00 | 0.89 | 0.58 |
| 197627061 | ArsC | Cyanobium sp. PCC 7001 | 0.00 | 0.00 | 0.00 | 0.63 | 0.00 |
| 55773483 | aoxB | Thermus thermophilus HB8 | 0.63 | 0.00 | 0.94 | 0.98 | 0.90 |
| 127512768 | ArsC | Shewanella loihica PV-4 | 0.00 | 0.00 | 0.00 | 0.00 | 0.98 |
| 108463091 | ArsC | Myxococcus xanthus DK 1622 | 0.00 | 0.00 | 0.00 | 0.65 | 0.65 |
| 23821270 | aoxB | Thiomonas sp. VB-2002 | 0.00 | 0.00 | 0.60 | 0.65 | 0.63 |
| 150956671 | ArsC | Klebsiella pneumoniae subsp. pneumoniae MGH 78578 | 0.61 | 0.00 | 0.89 | 0.93 | 0.92 |
| 209980176 | aoxB | Hydrogenobaculum sp. 3684 | 0.00 | 0.00 | 0.00 | 0.62 | 0.00 |
| 114319987 | ArsC | Alkalilimnicola ehrlichei MLHE-1 | 0.00 | 0.00 | 0.89 | 0.93 | 0.58 |
| 2822401 | ArsA | Halobacterium sp. NRC-1 | 0.58 | 0.00 | 0.86 | 0.62 | 0.89 |
| 12724358 | ArsC | Lactococcus lactis subsp. lactis Il1403 | 0.00 | 0.61 | 0.00 | 0.94 | 0.61 |
| 162568513 | aoxB | Pseudomonas sp. 89 | 0.00 | 0.00 | 0.92 | 0.95 | 0.91 |
| 162568555 | aoxB | uncultured bacterium | 0.00 | 0.00 | 0.00 | 0.64 | 0.96 |
| 152072156 | aoxB | Beggiatoa sp. PS | 0.00 | 0.00 | 0.00 | 0.00 | 0.57 |
| 193077109 | ArsC | Acinetobacter baumannii ATCC 17978 | 0.00 | 0.00 | 0.00 | 0.98 | 0.62 |
| 151281782 | ArsC | Janthinobacterium sp. Marseille | 0.00 | 0.00 | 0.00 | 0.00 | 0.95 |
| 212635535 | ArsC | Shewanella piezotolerans WP3 | 0.00 | 0.58 | 0.56 | 0.00 | 0.87 |
| 156531217 | ArsC | Enterobacter sakazakii ATCC BAA-894 | 0.78 | 0.82 | 0.82 | 0.00 | 0.00 |
| 12002188 | arsB | IncN plasmid R46 | 0.00 | 0.00 | 0.00 | 0.63 | 0.00 |
| 190012284 | ArsC | Stenotrophomonas maltophilia K279a | 0.00 | 0.61 | 0.00 | 0.00 | 1.00 |
| 222524598 | aoxB | Chloroflexus sp. Y-400-fl | 0.00 | 0.00 | 0.00 | 0.54 | 0.00 |
| 152992971 | arsB | Sulfurovum sp. NBC37-1 | 0.00 | 0.00 | 0.00 | 0.85 | 0.94 |
| 126641498 | ArsC | Acinetobacter baumannii ATCC 17978 | 0.00 | 0.00 | 0.00 | 0.60 | 0.00 |
| 35210564 | ArsC | Gloeobacter violaceus PCC 7421 | 0.00 | 0.00 | 0.00 | 0.58 | 0.00 |
| 159029820 | ArsC | Microcystis aeruginosa PCC 7806 | 0.93 | 0.62 | 0.96 | 0.97 | 0.89 |
| 134093762 | ArsC | Herminiimonas arsenicoxydans | 0.00 | 0.00 | 0.00 | 0.00 | 0.84 |
| 161327704 | ArsC | Shewanella benthica KT99 | 0.00 | 0.00 | 0.00 | 0.00 | 0.54 |
| 37962697 | aoxB | arsenite-oxidising bacterium NT-26 | 0.00 | 0.00 | 0.58 | 0.94 | 0.59 |
| 156976331 | ArsC | Vibrio harveyi ATCC BAA-1116 | 0.00 | 0.00 | 0.00 | 0.00 | 0.90 |
| 76788546 | ArsC | Streptococcus agalactiae A909 | 0.00 | 0.00 | 0.00 | 0.56 | 0.56 |
| 146284583 | ArsC | Enterobacter sp. 638 | 0.00 | 0.00 | 0.00 | 0.61 | 0.88 |
| 213971802 | ArsC | Pseudomonas syringae pv. tomato T1 | 0.00 | 0.00 | 0.00 | 0.00 | 0.87 |
| 126097353 | ArsC | Actinobacillus pleuropneumoniae L20 | 0.00 | 0.00 | 0.00 | 0.00 | 0.56 |
| 66769849 | ArsC | Xanthomonas campestris pv. campestris str. 8004 | 0.62 | 0.00 | 0.00 | 0.63 | 0.96 |
| 134292508 | ArsC | Burkholderia vietnamiensis G4 | 0.00 | 0.00 | 0.00 | 0.00 | 0.91 |
| 145297644 | ArsA | Aeromonas salmonicida subsp. salmonicida A449 | 0.00 | 0.00 | 0.00 | 0.00 | 0.58 |
| 218548072 | ArsC | Escherichia fergusonii ATCC 35469 | 0.00 | 0.00 | 0.00 | 0.00 | 0.56 |
| 94309274 | ArsC | Ralstonia metallidurans CH34 | 0.00 | 0.00 | 0.00 | 0.00 | 0.89 |
| 220721284 | ArsC | Variovorax paradoxus S110 | 0.00 | 0.00 | 0.00 | 0.00 | 0.85 |
| 110623044 | arsB | Pseudoclavibacter helvolus | 0.00 | 0.00 | 0.00 | 0.00 | 0.87 |
| 251844850 | arsM | Paenibacillus sp. oral taxon 786 str. D14 | 0.00 | 0.00 | 0.59 | 0.00 | 0.98 |
| 116254241 | ArsA | Rhizobium leguminosarum bv. viciae 3841 | 0.00 | 0.00 | 0.00 | 0.59 | 0.62 |
| 198038142 | ArsC | Burkholderia cenocepacia J2315 | 0.61 | 0.00 | 0.60 | 0.63 | 0.94 |
| 144899345 | ArsC | Magnetospirillum gryphiswaldense MSR-1 | 0.00 | 0.00 | 0.00 | 0.00 | 0.91 |
| 17741950 | arsB | Agrobacterium tumefaciens str. C58 | 0.00 | 0.00 | 0.00 | 0.60 | 0.00 |
| 212546787 | ArsC | Penicillium marneffei ATCC 18224 | 0.00 | 0.00 | 0.00 | 0.57 | 0.55 |
| 125716460 | aoxB | Arthrobacter sp. 15b | 0.00 | 0.00 | 0.00 | 0.88 | 0.57 |
| 118434236 | ArsC | Stappia aggregata IAM 12614 | 0.00 | 0.00 | 0.00 | 0.00 | 0.93 |
| 34482869 | arsB | Wolinella succinogenes | 0.00 | 0.00 | 0.00 | 0.00 | 0.57 |
| 167571826 | ArsC | Burkholderia oklahomensis C6786 | 0.00 | 0.00 | 0.00 | 0.57 | 0.82 |
| 110645039 | ArsC | Acidithiobacillus caldus | 0.00 | 0.00 | 0.57 | 0.59 | 0.57 |
| 68169267 | ArsC | Desulfitobacterium hafniense DCB-2 | 0.00 | 0.00 | 0.00 | 0.00 | 0.59 |
| 145568841 | ArsC | Pseudomonas stutzeri A1501 | 0.00 | 0.00 | 0.00 | 0.62 | 0.61 |
| 114228251 | ArsA | Alkalilimnicola ehrlichei MLHE-1 | 0.00 | 0.00 | 0.00 | 0.64 | 0.61 |
| 71065515 | arsB | Psychrobacter arcticus 273-4 | 0.00 | 0.00 | 0.00 | 0.00 | 0.88 |
| 70725200 | arsB | Staphylococcus haemolyticus JCSC1435 | 0.00 | 0.00 | 0.00 | 0.54 | 0.89 |
| 24984301 | ArsC | Pseudomonas putida KT2440 | 0.00 | 0.00 | 0.97 | 1.00 | 0.99 |
| 91800661 | ArsC | Nitrobacter hamburgensis X14 | 0.00 | 0.00 | 0.00 | 0.60 | 0.89 |
| 171699365 | ArsC | Cyanothece sp. ATCC 51142 | 0.00 | 0.00 | 0.00 | 0.00 | 0.89 |
| 154247889 | ArsC | Xanthobacter autotrophicus Py2 | 0.00 | 0.00 | 0.00 | 0.00 | 0.91 |
| 254448130 | arsM | gamma proteobacterium HTCC5015 | 0.00 | 0.00 | 0.00 | 0.57 | 0.90 |
| 190572256 | ArsC | Stenotrophomonas maltophilia K279a | 0.00 | 0.00 | 0.00 | 0.61 | 0.88 |
| 25027433 | ArsC | Corynebacterium efficiens YS-314 | 0.00 | 0.00 | 0.00 | 0.00 | 0.87 |
| 151562410 | ArsC | Ochrobactrum anthropi ATCC 49188 | 0.00 | 0.00 | 0.00 | 0.61 | 0.92 |
| 117923602 | ArsC | Magnetococcus sp. MC-1 | 0.00 | 0.00 | 0.00 | 0.00 | 0.58 |
| 187720241 | ArsC | Burkholderia phytofirmans PsJN | 0.97 | 0.00 | 1.01 | 0.00 | 0.69 |
| 82945771 | ArsC | Magnetospirillum magneticum AMB-1 | 0.00 | 0.00 | 0.00 | 0.86 | 0.00 |
| 71038664 | ArsA | Psychrobacter arcticus 273-4 | 0.00 | 0.00 | 0.00 | 0.00 | 0.55 |
| 169237596 | ArsA | Halobacterium salinarum R1 | 0.00 | 0.00 | 0.00 | 0.58 | 0.00 |
| 88774234 | ArsC | Alteromonas macleodii 'Deep ecotype' | 0.00 | 0.00 | 0.00 | 0.57 | 0.57 |
| 110647516 | ArsC | Alcanivorax borkumensis SK2 | 0.00 | 0.00 | 0.00 | 0.57 | 0.00 |
| 149805612 | aoxB | Moritella sp. PE36 | 0.00 | 0.00 | 0.00 | 0.57 | 0.00 |
| 188576466 | ArsC | Xanthomonas oryzae pv. oryzae PXO99A | 0.00 | 0.00 | 0.00 | 0.85 | 0.00 |
| 257779458 | arsM | Desulfotomaculum acetoxidans DSM 771 | 0.00 | 0.00 | 0.00 | 0.64 | 0.00 |
| 227901843 | arsM | Finegoldia magna ATCC 53516 | 0.00 | 0.00 | 0.00 | 0.00 | 0.57 |
| 158443876 | ArsC | Faecalibacterium prausnitzii M21/2 | 0.00 | 0.00 | 0.00 | 0.55 | 0.00 |
| 68053499 | ArsA | Leptospirillum ferriphilum | 0.00 | 0.00 | 0.00 | 0.55 | 0.00 |
| 56419124 | ArsC | Geobacillus kaustophilus HTA426 | 0.00 | 0.00 | 0.00 | 0.55 | 0.00 |
| 162568589 | aoxB | uncultured bacterium | 0.68 | 0.00 | 0.70 | 0.00 | 0.00 |

Table D Mantel test of the relationship between the structure of genes involved in As methylation (*arsM*), oxidation (*aoxB*) and arsenite efflux (*arsA* and *arsB*) and arsenate reduction (*arsC*).

| Arsenic resistance genes | *arsM* | | *aoxB* | |
| --- | --- | --- | --- | --- |
|  | r | p | r | p |
| *arsA* | 0.513 | 0.001 | 0.407 | 0.002 |
| *arsB* | 0.575 | 0.001 | 0.625 | 0.001 |
| *arsC* | 0.526 | 0.001 | 0.642 | 0.001 |

Table E Diversity indices of the As functional genes of the five geographically distributed soils.

| Soil ID | Richness^a^ | H^b^ | Evenness^c^ |
| --- | --- | --- | --- |
| B1 | 462±27ab | 6.13±0.10ab | 0.994±0.000a |
| B2 | 504±11a | 6.22±0.04a | 0.992±0.000b |
| C1 | 425±22b | 6.05±0.09bc | 0.993±0.000ab |
| C2 | 381±12bc | 5.94±0.06c | 0.993±0.000b |
| UK | 328±9c | 5.79±0.05d | 0.993±0.000ab |
| Note: Means ± SD in columns followed the same letter(s) are not statistically significant at 5 % significance level. ^a^ Detected As related functional gene species. ^b^ Shannon-Wiener index: higher numbers represent higher levels of diversity. ^c^ Evenness index. | | | |

Table F Sequences of all orders in each soil.

|  | B1 | B2 | C1 | C2 | UK |
| --- | --- | --- | --- | --- | --- |
| Acidimicrobiales | 57.64 | 59.32 | 37.55 | 36.94 | 18.48 |
| Actinomycetales | 95.18 | 396.22 | 120.18 | 83.42 | 97.24 |
| Alphaproteobacteria_incertae_sedis | 39.50 | 28.51 | 16.18 | 20.00 | 13.48 |
| Alteromonadales | 5.04 | 1.92 | 14.24 | 3.67 | 3.62 |
| Anaerolineales | 559.80 | 249.06 | 502.42 | 733.88 | 65.36 |
| Armatimonadales | 0.39 | 0.77 | 2.95 | 0.36 | 1.09 |
| Bacillales | 120.74 | 177.99 | 80.77 | 78.53 | 123.09 |
| Bacteroidales | 16.62 | 19.26 | 233.10 | 179.70 | 36.67 |
| Bdellovibrionales | 27.89 | 19.55 | 39.54 | 27.79 | 80.21 |
| Burkholderiales | 398.52 | 361.06 | 614.31 | 1174.12 | 755.03 |
| Caldilineales | 30.59 | 23.89 | 17.99 | 40.21 | 4.37 |
| Caulobacterales | 25.97 | 98.05 | 296.41 | 245.10 | 380.68 |
| Chlamydiales | 0.39 | 7.70 | 0.00 | 0.00 | 0.00 |
| Chloroflexales | 0.39 | 1.93 | 2.96 | 6.25 | 0.00 |
| Chromatiales | 29.43 | 22.51 | 9.71 | 12.16 | 6.14 |
| Chthonomonadales | 6.94 | 9.80 | 8.28 | 2.93 | 2.17 |
| Clostridiales | 109.86 | 145.26 | 658.24 | 896.75 | 942.42 |
| Coriobacteriales | 8.49 | 8.90 | 42.80 | 64.86 | 25.02 |
| Deinococcales | 0.00 | 0.00 | 0.00 | 0.00 | 5.82 |
| Desulfarculales | 5.03 | 0.00 | 0.00 | 0.39 | 0.00 |
| Desulfobacterales | 18.97 | 16.68 | 27.67 | 27.04 | 15.63 |
| Desulfovibrionales | 6.20 | 3.49 | 21.01 | 7.42 | 13.43 |
| Desulfurococcales | 0.77 | 3.67 | 1.49 | 0.00 | 2.18 |
| Desulfuromonadales | 121.22 | 118.16 | 225.59 | 316.42 | 455.38 |
| Enterobacteriales | 5.03 | 4.25 | 4.88 | 25.61 | 2.90 |
| Erysipelotrichales | 0.00 | 0.00 | 0.37 | 0.36 | 0.00 |
| Fervidicoccales | 299.23 | 94.50 | 118.61 | 122.30 | 23.94 |
| Flavobacteriales | 26.68 | 65.10 | 217.67 | 254.78 | 196.58 |
| Gammaproteobacteria_incertae_sedis | 27.13 | 12.94 | 65.36 | 10.82 | 6.18 |
| Gemmatimonadales | 113.75 | 141.25 | 91.81 | 93.68 | 229.60 |
| Halanaerobiales | 0.00 | 4.64 | 5.24 | 5.16 | 0.73 |
| Herpetosiphonales | 0.00 | 1.59 | 0.00 | 0.00 | 0.00 |
| Holophagales | 0.00 | 0.38 | 1.49 | 5.81 | 0.00 |
| Hydrogenophilales | 1.55 | 4.65 | 17.30 | 6.24 | 10.15 |
| Ignavibacteriales | 23.21 | 49.43 | 92.76 | 75.98 | 121.21 |
| Kiloniellales | 0.00 | 0.38 | 0.38 | 0.75 | 0.00 |
| Ktedonobacterales | 0.00 | 1.56 | 0.00 | 0.00 | 0.00 |
| Lactobacillales | 0.00 | 0.00 | 1.87 | 2.22 | 0.00 |
| Legionellales | 10.49 | 18.16 | 6.38 | 4.49 | 7.24 |
| Methanobacteriales | 12.37 | 5.45 | 6.36 | 13.14 | 0.00 |
| Methanocellales | 0.39 | 0.38 | 4.14 | 4.12 | 0.72 |
| Methanomicrobiales | 0.00 | 0.00 | 0.37 | 0.78 | 0.00 |
| Methanosarcinales | 0.77 | 0.00 | 1.12 | 1.47 | 0.73 |
| Methylococcales | 11.22 | 7.42 | 91.08 | 61.04 | 18.12 |
| Methylophilales | 0.00 | 2.41 | 0.00 | 3.39 | 0.00 |
| Myxococcales | 407.53 | 358.44 | 488.77 | 502.95 | 259.90 |
| Natranaerobiales | 23.59 | 12.22 | 17.26 | 10.84 | 33.68 |
| Neisseriales | 53.90 | 41.34 | 15.73 | 16.06 | 49.42 |
| Nitrosomonadales | 43.01 | 91.31 | 37.57 | 19.40 | 33.41 |
| Nitrospirales | 110.78 | 57.13 | 28.53 | 6.87 | 11.96 |
| Oceanospirillales | 8.54 | 10.49 | 9.37 | 29.29 | 0.73 |
| Opitutales | 12.77 | 23.02 | 70.23 | 25.34 | 65.78 |
| Parvularculales | 0.00 | 1.59 | 0.00 | 0.00 | 0.37 |
| Phycisphaerales | 6.59 | 3.10 | 1.89 | 1.08 | 0.73 |
| Planctomycetales | 292.17 | 255.06 | 116.26 | 165.21 | 288.03 |
| Pseudomonadales | 36.01 | 20.05 | 38.72 | 25.09 | 8.70 |
| Rhizobiales | 249.98 | 406.23 | 323.06 | 413.07 | 467.34 |
| Rhodobacterales | 27.90 | 29.18 | 102.22 | 44.79 | 17.45 |
| Rhodocyclales | 488.74 | 260.46 | 319.67 | 164.23 | 163.73 |
| Rhodospirillales | 100.26 | 97.45 | 172.19 | 160.88 | 290.59 |
| Rickettsiales | 1.16 | 0.00 | 3.00 | 5.51 | 2.53 |
| Rubrobacterales | 1.54 | 0.82 | 0.00 | 0.00 | 0.00 |
| Solirubrobacterales | 145.50 | 171.54 | 112.32 | 97.77 | 38.82 |
| Sphaerobacterales | 1.55 | 3.55 | 0.37 | 0.00 | 0.37 |
| Sphingobacteriales | 381.72 | 391.91 | 644.67 | 612.36 | 692.71 |
| Sphingomonadales | 235.73 | 427.45 | 165.30 | 178.00 | 296.17 |
| Spirochaetales | 1.55 | 0.00 | 33.09 | 9.40 | 23.83 |
| Syntrophobacterales | 83.95 | 59.65 | 60.34 | 39.58 | 22.92 |
| Thermoanaerobacterales | 8.51 | 4.67 | 8.25 | 12.25 | 7.64 |
| Thermogemmatisporales | 0.00 | 2.78 | 0.00 | 0.00 | 0.00 |
| Thermoleophilales | 57.27 | 48.62 | 80.48 | 33.45 | 5.80 |
| Thermoplasmatales | 0.78 | 0.00 | 9.02 | 9.19 | 1.09 |
| Thermoproteales | 5.01 | 4.28 | 8.62 | 6.25 | 1.09 |
| Thiotrichales | 17.87 | 5.47 | 0.76 | 1.14 | 0.73 |
| Verrucomicrobiales | 10.87 | 21.97 | 19.85 | 19.46 | 19.19 |
| Xanthomonadales | 212.13 | 154.69 | 102.07 | 52.38 | 99.06 |

Table G Sequences of all families in each soil.

|  | B1 | B2 | C1 | C2 | UK |
| --- | --- | --- | --- | --- | --- |
| Anaerolineaceae | 559.80 | 249.06 | 502.42 | 733.88 | 65.36 |
| Chitinophagaceae | 300.34 | 328.41 | 561.89 | 518.12 | 461.89 |
| Planctomycetaceae | 292.17 | 255.06 | 116.26 | 165.21 | 288.03 |
| Sphingomonadaceae | 211.32 | 411.26 | 152.27 | 129.11 | 256.99 |
| Sinobacteraceae | 137.41 | 36.44 | 44.66 | 16.69 | 4.73 |
| Rhodocyclaceae | 126.26 | 57.23 | 251.85 | 125.82 | 125.66 |
| Hyphomicrobiaceae | 121.45 | 192.66 | 123.46 | 141.96 | 171.58 |
| Comamonadaceae | 118.43 | 126.63 | 318.04 | 949.54 | 392.96 |
| Gemmatimonadaceae | 113.75 | 141.25 | 91.81 | 93.68 | 229.60 |
| Nitrospiraceae | 110.78 | 57.13 | 28.53 | 6.87 | 11.96 |
| Cystobacteraceae | 109.85 | 90.59 | 144.31 | 173.21 | 169.81 |
| Oxalobacteraceae | 102.32 | 74.68 | 156.58 | 93.94 | 252.29 |
| Fervidicoccaceae | 99.00 | 13.76 | 80.76 | 115.52 | 3.64 |
| Rhodospirillaceae | 73.95 | 52.50 | 116.73 | 77.12 | 165.60 |
| Polyangiaceae | 72.75 | 62.53 | 121.66 | 95.72 | 21.44 |
| Solirubrobacteraceae | 64.60 | 61.80 | 35.63 | 24.23 | 15.23 |
| Xanthomonadaceae | 63.49 | 105.36 | 51.78 | 29.80 | 90.71 |
| Pasteuriaceae | 59.21 | 36.10 | 24.76 | 19.45 | 9.06 |
| Conexibacteraceae | 58.80 | 73.10 | 64.25 | 40.37 | 11.97 |
| Geobacteraceae | 55.74 | 79.62 | 196.66 | 301.76 | 451.73 |
| Kofleriaceae | 45.30 | 29.73 | 33.39 | 21.80 | 9.07 |
| Cytophagaceae | 40.31 | 20.21 | 21.66 | 24.82 | 201.76 |
| Bacillaceae 1 | 37.14 | 88.55 | 18.81 | 9.69 | 52.66 |
| Acidimicrobineae_incertae_sedis | 35.99 | 33.03 | 21.05 | 20.77 | 5.43 |
| Myxococcaceae | 35.98 | 32.56 | 38.28 | 15.97 | 10.15 |
| Clostridiaceae 1 | 32.86 | 63.33 | 274.11 | 274.42 | 251.27 |
| Rhodobiaceae | 31.76 | 17.68 | 17.60 | 9.02 | 17.09 |
| Caldilineaceae | 30.59 | 23.89 | 17.99 | 40.21 | 4.37 |
| Burkholderiaceae | 30.22 | 68.23 | 21.36 | 14.80 | 21.42 |
| Phaselicystidaceae | 28.66 | 15.33 | 17.70 | 15.52 | 6.52 |
| Desulfuromonadaceae | 27.11 | 2.34 | 14.67 | 5.29 | 1.46 |
| Bradyrhizobiaceae | 26.30 | 50.90 | 25.57 | 37.02 | 24.31 |
| Cryomorphaceae | 26.30 | 45.29 | 212.76 | 234.71 | 75.45 |
| Thermoleophilaceae | 23.97 | 12.13 | 7.92 | 5.55 | 3.27 |
| Ignavibacteriaceae | 23.21 | 49.43 | 92.76 | 75.98 | 121.21 |
| Bdellovibrionaceae | 22.87 | 9.87 | 27.20 | 13.24 | 64.95 |
| Haliangiaceae | 22.82 | 54.19 | 50.63 | 37.38 | 12.74 |
| Erythrobacteraceae | 22.09 | 14.15 | 1.90 | 40.80 | 29.01 |
| Acetobacteraceae | 21.67 | 32.39 | 13.49 | 29.27 | 50.09 |
| Alcaligenaceae | 20.49 | 12.94 | 9.01 | 5.60 | 8.30 |
| Saprospiraceae | 19.39 | 19.27 | 32.86 | 1.87 | 2.18 |
| Ruminococcaceae | 19.37 | 9.06 | 47.72 | 73.34 | 105.43 |
| Micromonosporaceae | 17.80 | 40.08 | 34.19 | 7.30 | 5.06 |
| Caulobacteraceae | 17.08 | 37.34 | 244.79 | 219.56 | 311.27 |
| Syntrophaceae | 17.03 | 6.59 | 21.75 | 21.33 | 0.00 |
| Marinilabiaceae | 16.62 | 19.26 | 231.97 | 179.70 | 36.67 |
| Acidimicrobiaceae | 15.84 | 15.28 | 9.36 | 8.82 | 11.94 |
| Rhodobacteraceae | 15.50 | 19.41 | 65.20 | 23.00 | 11.64 |
| Xanthobacteraceae | 15.12 | 49.90 | 13.51 | 32.69 | 49.30 |
| Syntrophobacteraceae | 13.91 | 5.85 | 16.88 | 4.83 | 22.56 |
| Nannocystaceae | 13.19 | 13.74 | 17.63 | 17.46 | 2.17 |
| Desulfobacteraceae | 13.18 | 0.80 | 6.34 | 13.03 | 0.36 |
| Family I | 12.79 | 9.82 | 4.83 | 18.79 | 71.43 |
| Neisseriaceae | 12.79 | 5.91 | 2.25 | 2.92 | 10.11 |
| Opitutaceae | 12.77 | 23.02 | 70.23 | 25.34 | 65.78 |
| Methanobacteriaceae | 12.37 | 5.45 | 6.36 | 13.14 | 0.00 |
| Gracilibacteraceae | 12.36 | 26.19 | 132.90 | 207.34 | 195.75 |
| Rhizobiales_incertae_sedis | 11.97 | 20.78 | 46.49 | 33.41 | 34.89 |
| Verrucomicrobiaceae | 10.87 | 21.97 | 19.85 | 19.46 | 19.19 |
| Nocardioidaceae | 10.86 | 53.25 | 13.89 | 3.99 | 6.89 |
| Sphingobacteriaceae | 10.06 | 20.14 | 21.48 | 4.42 | 24.72 |
| Methylobacteriaceae | 9.68 | 11.78 | 6.40 | 3.74 | 6.53 |
| Mycobacteriaceae | 9.29 | 38.18 | 10.09 | 19.25 | 18.12 |
| Beijerinckiaceae | 8.91 | 15.74 | 19.57 | 18.24 | 47.56 |
| Methylococcaceae | 8.91 | 4.67 | 83.89 | 59.57 | 15.58 |
| Rhodothermaceae | 8.90 | 0.77 | 0.75 | 61.68 | 0.00 |
| Intrasporangiaceae | 8.54 | 36.16 | 18.75 | 14.33 | 22.13 |
| Coriobacteriaceae | 8.49 | 8.90 | 42.80 | 64.86 | 25.02 |
| Coxiellaceae | 8.17 | 9.84 | 2.61 | 0.36 | 0.36 |
| Streptomycetaceae | 8.11 | 21.13 | 3.40 | 1.14 | 3.27 |
| Paenibacillaceae 1 | 7.73 | 9.93 | 3.39 | 4.43 | 9.07 |
| Veillonellaceae | 7.35 | 8.95 | 33.44 | 83.55 | 66.74 |
| Pseudonocardiaceae | 7.34 | 30.87 | 1.51 | 0.36 | 1.81 |
| Syntrophorhabdaceae | 6.97 | 5.84 | 27.02 | 13.34 | 0.36 |
| Chthonomonadaceae | 6.94 | 9.80 | 8.28 | 2.93 | 2.17 |
| Phycisphaeraceae | 6.59 | 3.10 | 1.89 | 1.08 | 0.73 |
| Aurantimonadaceae | 6.58 | 3.96 | 1.12 | 3.65 | 1.09 |
| Phyllobacteriaceae | 6.19 | 12.25 | 10.09 | 19.37 | 11.26 |
| Iamiaceae | 5.81 | 11.00 | 7.14 | 7.35 | 1.10 |
| Planococcaceae | 5.79 | 29.39 | 26.30 | 39.76 | 41.01 |
| Peptococcaceae 2 | 5.43 | 5.04 | 11.27 | 11.99 | 13.40 |
| Alicyclobacillaceae | 5.43 | 7.81 | 1.50 | 1.83 | 0.00 |
| Family XIII | 5.03 | 5.82 | 450.29 | 20.81 | 6.91 |
| Desulfobulbaceae | 5.02 | 11.18 | 18.69 | 13.23 | 7.25 |
| Methylocystaceae | 4.65 | 13.59 | 10.13 | 21.51 | 4.00 |
| Chromatiaceae | 4.64 | 0.00 | 0.38 | 0.00 | 0.00 |
| Jiangellaceae | 4.63 | 0.00 | 0.37 | 0.00 | 0.00 |
| Clostridiales_Incertae Sedis XVIII | 4.26 | 0.00 | 0.76 | 2.57 | 0.36 |
| Nitrosomonadaceae | 3.88 | 10.66 | 27.51 | 13.05 | 31.59 |
| Family VIII | 3.87 | 1.17 | 11.82 | 0.36 | 0.00 |
| Acidothermaceae | 3.87 | 6.37 | 12.04 | 1.08 | 0.36 |
| Alteromonadaceae | 3.50 | 1.92 | 14.24 | 3.67 | 3.62 |
| Bacillaceae 2 | 3.50 | 1.96 | 2.27 | 0.36 | 2.18 |
| Desulfovibrionaceae | 3.48 | 2.70 | 18.77 | 4.82 | 12.34 |
| Family V | 3.48 | 3.08 | 4.51 | 7.32 | 0.36 |
| Lachnospiraceae | 3.47 | 0.76 | 7.88 | 17.25 | 14.11 |
| Heliobacteriaceae | 3.09 | 0.78 | 5.97 | 2.26 | 3.27 |
| Peptostreptococcaceae | 3.09 | 4.69 | 21.04 | 28.45 | 7.24 |
| Flammeovirgaceae | 2.72 | 3.11 | 6.03 | 1.44 | 2.16 |
| Family IX | 2.70 | 19.15 | 31.61 | 6.19 | 0.36 |
| Pseudomonadaceae | 2.70 | 6.77 | 3.76 | 1.50 | 0.36 |
| Brucellaceae | 2.33 | 7.14 | 0.00 | 6.54 | 0.36 |
| Bacteriovoracaceae | 2.32 | 6.53 | 6.76 | 5.56 | 0.36 |
| Microbacteriaceae | 2.32 | 11.62 | 5.65 | 3.32 | 4.74 |
| Enterobacteriaceae | 1.93 | 3.45 | 3.76 | 25.61 | 2.54 |
| Legionellaceae | 1.93 | 2.00 | 0.38 | 0.36 | 0.00 |
| Streptosporangiaceae | 1.93 | 3.18 | 0.00 | 0.78 | 1.45 |
| Clostridiales_Incertae Sedis XIII | 1.55 | 5.04 | 28.50 | 50.72 | 92.19 |
| Leptospiraceae | 1.55 | 0.00 | 31.22 | 9.40 | 23.83 |
| Rhizobiaceae | 1.55 | 7.92 | 39.03 | 74.53 | 92.13 |
| Sphaerobacteraceae | 1.55 | 3.55 | 0.37 | 0.00 | 0.37 |
| Hyphomonadaceae | 1.55 | 1.55 | 16.01 | 7.37 | 18.85 |
| Rubrobacteraceae | 1.54 | 0.82 | 0.00 | 0.00 | 0.00 |
| Clostridiaceae 4 | 1.16 | 0.00 | 10.12 | 3.74 | 0.00 |
| Clostridiales_Incertae Sedis XI | 1.16 | 1.55 | 11.68 | 22.13 | 20.31 |
| Geodermatophilaceae | 1.16 | 34.79 | 3.39 | 1.84 | 3.28 |
| Rickettsiaceae | 1.16 | 0.00 | 2.25 | 5.51 | 2.53 |
| Sutterellaceae | 1.16 | 0.00 | 0.37 | 0.00 | 0.00 |
| Nocardiaceae | 1.16 | 7.14 | 1.50 | 0.00 | 2.55 |
| Thermoactinomycetaceae 1 | 1.16 | 1.16 | 3.36 | 1.89 | 8.37 |
| Syntrophomonadaceae | 1.16 | 1.17 | 10.18 | 12.09 | 7.97 |
| Ectothiorhodospiraceae | 1.16 | 0.00 | 3.70 | 1.11 | 0.36 |
| Bacillales_incertae_sedis | 0.78 | 0.00 | 0.00 | 0.00 | 0.00 |
| Clostridiaceae 3 | 0.78 | 1.58 | 3.01 | 2.56 | 2.90 |
| Micrococcaceae | 0.78 | 60.01 | 3.01 | 3.67 | 21.08 |
| Thermoplasmatales_incertae_sedis | 0.78 | 0.00 | 9.02 | 9.19 | 1.09 |
| Kineosporiaceae | 0.77 | 4.76 | 0.00 | 0.00 | 0.36 |
| Methanosarcinaceae | 0.77 | 0.00 | 1.12 | 1.47 | 0.73 |
| Desulfarculaceae | 0.77 | 0.00 | 0.00 | 0.00 | 0.00 |
| Family XI | 0.77 | 0.00 | 0.38 | 2.21 | 0.00 |
| Desulfohalobiaceae | 0.39 | 0.00 | 1.51 | 1.11 | 0.00 |
| Incertae Sedis III | 0.39 | 1.98 | 0.00 | 0.72 | 0.00 |
| Chloroflexaceae | 0.39 | 1.93 | 2.96 | 6.25 | 0.00 |
| Flavobacteriaceae | 0.39 | 19.81 | 4.91 | 20.07 | 121.13 |
| Moraxellaceae | 0.39 | 1.58 | 14.74 | 9.94 | 5.81 |
| Nakamurellaceae | 0.39 | 0.82 | 0.38 | 0.00 | 0.72 |
| Peptococcaceae I | 0.39 | 0.77 | 0.00 | 1.11 | 1.44 |
| Thermomonosporaceae | 0.39 | 5.09 | 1.90 | 4.47 | 0.73 |
| Armatimonadaceae | 0.39 | 0.77 | 2.95 | 0.36 | 1.09 |
| Eubacteriaceae | 0.39 | 0.77 | 6.76 | 9.72 | 13.44 |
| Methanocellaceae | 0.39 | 0.38 | 4.14 | 4.12 | 0.72 |
| Parachlamydiaceae | 0.39 | 1.17 | 0.00 | 0.00 | 0.00 |
| Peptococcaceae 1 | 0.39 | 2.36 | 16.17 | 10.40 | 29.03 |
| Thiotrichales_incertae_sedis | 0.39 | 0.00 | 0.00 | 0.39 | 0.00 |
| Carnobacteriaceae | 0.00 | 0.00 | 1.11 | 1.11 | 0.00 |
| Catenulisporaceae | 0.00 | 0.80 | 0.00 | 0.00 | 0.00 |
| Cellulomonadaceae | 0.00 | 1.56 | 3.00 | 13.36 | 4.34 |
| Clostridiaceae 2 | 0.00 | 0.77 | 5.26 | 2.97 | 13.39 |
| Clostridiales_Incertae Sedis IV | 0.00 | 0.00 | 0.00 | 0.36 | 7.25 |
| Clostridiales_Incertae Sedis XII | 0.00 | 0.00 | 1.10 | 6.77 | 10.53 |
| Clostridiales_Incertae Sedis XVI | 0.00 | 0.00 | 1.51 | 0.00 | 0.37 |
| Cohaesibacteraceae | 0.00 | 0.00 | 1.84 | 0.00 | 0.00 |
| Corynebacterineae_incertae_sedis | 0.00 | 0.00 | 0.00 | 0.39 | 0.00 |
| Deinococcaceae | 0.00 | 0.00 | 0.00 | 0.00 | 5.09 |
| Erysipelotrichaceae | 0.00 | 0.00 | 0.37 | 0.36 | 0.00 |
| Family IV | 0.00 | 0.00 | 1.52 | 0.00 | 0.00 |
| Family X | 0.00 | 0.00 | 1.48 | 0.00 | 0.00 |
| Herpetosiphonaceae | 0.00 | 1.59 | 0.00 | 0.00 | 0.00 |
| Holophagaceae | 0.00 | 0.38 | 1.49 | 5.81 | 0.00 |
| Hydrogenophilaceae | 0.00 | 0.00 | 10.89 | 0.00 | 2.18 |
| Incertae Sedis XI | 0.00 | 0.41 | 4.50 | 8.49 | 27.26 |
| Ktedonobacteraceae | 0.00 | 0.79 | 0.00 | 0.00 | 0.00 |
| Methanomicrobiales_incertae_sedis | 0.00 | 0.00 | 0.37 | 0.78 | 0.00 |
| Methylophilaceae | 0.00 | 2.41 | 0.00 | 3.39 | 0.00 |
| Natranaerobiaceae | 0.00 | 0.00 | 0.00 | 0.00 | 0.73 |
| Paenibacillaceae 2 | 0.00 | 0.80 | 0.38 | 1.12 | 0.00 |
| Parvularculaceae | 0.00 | 1.59 | 0.00 | 0.00 | 0.37 |
| Porphyromonadaceae | 0.00 | 0.00 | 1.13 | 0.00 | 0.00 |
| Promicromonosporaceae | 0.00 | 1.61 | 0.00 | 0.00 | 0.00 |
| Simkaniaceae | 0.00 | 6.53 | 0.00 | 0.00 | 0.00 |
| Spirochaetaceae | 0.00 | 0.00 | 1.86 | 0.00 | 0.00 |
| Sporichthyaceae | 0.00 | 0.38 | 0.38 | 0.72 | 0.00 |
| Thermoactinomycetaceae 2 | 0.00 | 2.30 | 0.00 | 0.00 | 0.00 |
| Thiotrichaceae | 0.00 | 0.76 | 0.00 | 0.00 | 0.00 |

Table H Sequences of all genera in each soil.

|  | B1 | B2 | C1 | C2 | UK |
| --- | --- | --- | --- | --- | --- |
| Gp6 | 1231.85 | 1041.25 | 552.49 | 444.53 | 192.02 |
| Gp4 | 358.36 | 462.74 | 111.29 | 63.82 | 33.84 |
| Bellilinea | 343.40 | 153.33 | 303.75 | 363.62 | 9.82 |
| Flavisolibacter | 190.40 | 156.08 | 374.73 | 276.75 | 126.35 |
| Sphingomonas | 172.61 | 256.85 | 94.53 | 67.63 | 186.14 |
| Gp7 | 160.37 | 214.50 | 122.43 | 94.02 | 6.53 |
| Gp16 | 150.20 | 345.85 | 32.98 | 90.45 | 15.63 |
| Longilinea | 145.92 | 65.64 | 162.79 | 262.47 | 50.82 |
| Steroidobacter | 137.02 | 34.06 | 44.66 | 16.69 | 4.73 |
| Gp25 | 135.91 | 51.34 | 9.36 | 8.62 | 0.00 |
| Subdivision3_genera_incertae_sedis | 131.20 | 121.83 | 183.74 | 105.72 | 327.42 |
| Gp17 | 116.89 | 99.22 | 23.61 | 5.83 | 1.81 |
| Gemmatimonas | 113.75 | 141.25 | 91.81 | 93.68 | 229.60 |
| Nitrospira | 110.78 | 57.13 | 28.53 | 6.87 | 11.96 |
| Gemmata | 104.81 | 74.79 | 21.45 | 23.99 | 58.19 |
| Anaeromyxobacter | 100.18 | 79.21 | 127.01 | 165.85 | 163.27 |
| Fervidicoccus | 99.00 | 13.76 | 80.76 | 115.52 | 3.64 |
| Hyphomicrobium | 85.88 | 57.28 | 66.43 | 51.71 | 21.77 |
| Gp3 | 72.78 | 96.10 | 57.68 | 76.87 | 179.59 |
| Streptophyta | 69.50 | 12.89 | 8.91 | 3.05 | 10.51 |
| Zavarzinella | 67.76 | 50.23 | 21.03 | 12.84 | 76.64 |
| Pasteuria | 59.21 | 36.10 | 24.76 | 19.45 | 9.06 |
| Solirubrobacter | 58.41 | 52.68 | 32.64 | 23.48 | 9.43 |
| Spartobacteria_genera_incertae_sedis | 56.30 | 403.94 | 78.68 | 22.02 | 80.98 |
| Skermanella | 54.18 | 30.50 | 32.67 | 16.27 | 2.16 |
| Geobacter | 52.65 | 72.58 | 196.66 | 301.76 | 451.73 |
| Blastopirellula | 50.32 | 32.62 | 12.39 | 23.48 | 21.13 |
| Gp1 | 49.97 | 108.49 | 9.75 | 8.55 | 7.62 |
| Chondromyces | 47.62 | 36.81 | 50.63 | 48.96 | 11.27 |
| Leptolinea | 41.78 | 19.90 | 15.77 | 65.51 | 4.36 |
| Terrimonas | 37.20 | 51.93 | 20.65 | 11.74 | 32.50 |
| Gp10 | 34.86 | 48.00 | 20.39 | 8.42 | 14.53 |
| Oxalicibacterium | 31.75 | 24.43 | 29.33 | 14.37 | 65.48 |
| Armatimonadetes_gp5 | 31.36 | 28.69 | 28.44 | 17.78 | 16.79 |
| Caldilinea | 30.59 | 23.89 | 17.99 | 40.21 | 4.37 |
| Methyloversatilis | 29.80 | 10.16 | 10.86 | 7.70 | 5.07 |
| Isosphaera | 29.00 | 19.54 | 6.75 | 8.18 | 5.44 |
| Levilinea | 28.70 | 10.19 | 20.11 | 42.28 | 0.36 |
| Azospira | 27.44 | 5.80 | 4.10 | 1.48 | 13.02 |
| Massilia | 26.75 | 13.03 | 17.71 | 16.44 | 29.11 |
| Brachymonas | 26.28 | 4.43 | 0.00 | 1.14 | 0.00 |
| Acidovorax | 25.58 | 29.69 | 123.11 | 570.67 | 146.41 |
| Clostridium sensu stricto | 25.13 | 40.42 | 135.10 | 167.31 | 64.61 |
| Thermoleophilum | 23.97 | 12.13 | 7.92 | 5.55 | 3.27 |
| Bacillariophyta | 23.69 | 9.01 | 8.31 | 33.09 | 278.15 |
| Byssovorax | 23.59 | 18.36 | 57.47 | 39.41 | 4.36 |
| Ignavibacterium | 23.21 | 49.43 | 92.76 | 75.98 | 121.21 |
| Sphingosinicella | 22.85 | 129.62 | 25.91 | 40.56 | 23.94 |
| Gp21 | 22.81 | 22.19 | 7.86 | 2.96 | 0.00 |
| Conexibacter | 21.68 | 56.79 | 45.07 | 30.03 | 9.79 |
| Gp18 | 21.31 | 8.56 | 14.99 | 5.19 | 0.73 |
| Desulfuromonas | 20.54 | 0.78 | 8.28 | 4.51 | 1.10 |
| Anderseniella | 20.15 | 7.47 | 1.13 | 0.36 | 0.36 |
| Lysobacter | 19.71 | 17.00 | 22.48 | 1.83 | 1.82 |
| Singulisphaera | 18.97 | 44.57 | 8.98 | 14.83 | 34.45 |
| Ferruginibacter | 18.59 | 19.11 | 3.74 | 20.07 | 24.92 |
| Pontibacter | 18.20 | 0.00 | 1.50 | 0.00 | 1.82 |
| Adhaeribacter | 17.81 | 10.16 | 3.02 | 1.81 | 156.25 |
| Bacillus | 17.42 | 43.54 | 10.50 | 6.26 | 9.43 |
| Ramlibacter | 16.63 | 37.07 | 89.34 | 73.15 | 31.22 |
| Xenophilus | 16.25 | 17.29 | 23.66 | 88.48 | 14.94 |
| Armatimonadetes_gp4 | 15.88 | 15.72 | 2.64 | 2.59 | 6.17 |
| Geminicoccus | 15.49 | 2.76 | 0.38 | 0.00 | 0.00 |
| Gp22 | 15.45 | 5.46 | 1.12 | 0.73 | 0.00 |
| Luteimonas | 15.10 | 11.05 | 4.88 | 0.72 | 2.17 |
| Tepidimonas | 14.74 | 9.80 | 13.85 | 9.33 | 4.73 |
| Limnobacter | 14.74 | 4.65 | 8.22 | 4.05 | 3.26 |
| Planctomyces | 14.72 | 24.64 | 31.11 | 71.94 | 76.20 |
| Armatimonadetes_gp2 | 14.69 | 19.52 | 63.35 | 67.08 | 66.03 |
| Vampirovibrio | 14.36 | 3.14 | 10.05 | 3.31 | 57.34 |
| Phaselicystis | 14.34 | 3.55 | 3.75 | 6.58 | 1.09 |
| Kofleria | 14.33 | 9.68 | 6.01 | 7.81 | 1.45 |
| Gp5 | 13.59 | 16.32 | 10.10 | 3.34 | 1.82 |
| Arenimonas | 13.54 | 20.37 | 16.59 | 2.16 | 80.16 |
| Dechloromonas | 12.80 | 7.38 | 19.72 | 12.92 | 5.82 |
| GpI | 12.79 | 9.82 | 2.62 | 18.79 | 71.43 |
| Aciditerrimonas | 12.39 | 12.52 | 4.50 | 9.55 | 0.73 |
| Methanobacterium | 12.37 | 5.45 | 6.36 | 13.14 | 0.00 |
| Rhodoplanes | 12.35 | 50.18 | 7.13 | 33.59 | 7.28 |
| Altererythrobacter | 12.02 | 4.34 | 0.38 | 34.86 | 1.46 |
| WS3_genera_incertae_sedis | 12.01 | 16.96 | 4.83 | 9.06 | 7.64 |
| Pyxidicoccus | 11.58 | 16.03 | 6.02 | 7.45 | 2.17 |
| Desulfobacterium | 11.24 | 0.80 | 5.59 | 11.16 | 0.00 |
| Opitutus | 11.22 | 21.86 | 69.85 | 23.49 | 65.78 |
| Haliangium | 11.21 | 21.17 | 14.96 | 8.17 | 1.09 |
| BRC1_genera_incertae_sedis | 10.83 | 14.05 | 28.59 | 24.88 | 30.48 |
| Sulfuritalea | 10.51 | 6.98 | 19.24 | 7.79 | 8.70 |
| Phenylobacterium | 10.49 | 30.36 | 226.07 | 138.16 | 245.24 |
| Zoogloea | 10.48 | 7.04 | 12.36 | 15.49 | 14.41 |
| Marmoricola | 10.47 | 26.59 | 12.39 | 2.54 | 2.90 |
| Georgfuchsia | 10.42 | 5.03 | 140.93 | 11.51 | 5.45 |
| Bradyrhizobium | 10.05 | 26.78 | 4.11 | 7.02 | 5.44 |
| Gracilibacter | 9.67 | 17.58 | 80.30 | 112.32 | 80.46 |
| Mycobacterium | 9.29 | 38.18 | 10.09 | 19.25 | 18.12 |
| Aquabacterium | 9.28 | 2.36 | 1.88 | 1.51 | 0.00 |
| Sediminibacterium | 9.25 | 10.86 | 10.10 | 5.70 | 13.42 |
| Azoarcus | 8.93 | 5.13 | 7.51 | 8.18 | 7.61 |
| Ohtaekwangia | 8.91 | 5.48 | 7.09 | 3.32 | 48.25 |
| Clostridium III | 8.52 | 5.52 | 28.51 | 44.74 | 65.57 |
| Bdellovibrio | 8.51 | 6.73 | 17.15 | 9.93 | 7.61 |
| Olsenella | 8.49 | 8.90 | 42.80 | 64.86 | 25.02 |
| Aquicella | 8.17 | 9.84 | 2.61 | 0.36 | 0.36 |
| Rhizomicrobium | 8.15 | 13.04 | 6.36 | 9.32 | 11.65 |
| Azonexus | 8.14 | 4.27 | 16.11 | 10.36 | 33.35 |
| Niastella | 8.13 | 20.18 | 1.12 | 0.00 | 0.36 |
| Syntrophobacter | 8.12 | 1.17 | 3.01 | 3.72 | 11.66 |
| Pseudolabrys | 7.74 | 37.71 | 6.02 | 13.29 | 32.60 |
| Phycicoccus | 7.37 | 15.92 | 13.50 | 7.42 | 0.00 |
| Pseudofulvimonas | 7.35 | 3.55 | 0.00 | 0.36 | 0.36 |
| Desulfomonile | 6.95 | 1.16 | 1.49 | 12.06 | 0.00 |
| Chthonomonas/Armatimonadetes_gp3 | 6.94 | 9.80 | 8.28 | 2.93 | 2.17 |
| Rhodopirellula | 6.60 | 7.86 | 14.55 | 9.95 | 15.97 |
| Phycisphaera | 6.59 | 3.10 | 1.89 | 1.08 | 0.73 |
| Filimonas | 6.59 | 15.28 | 9.08 | 5.86 | 1.82 |
| Haliscomenobacter | 6.59 | 10.01 | 32.48 | 0.00 | 2.18 |
| Microvirga | 6.58 | 5.88 | 5.26 | 3.74 | 5.08 |
| Erythrobacter | 6.57 | 0.41 | 0.76 | 0.36 | 0.00 |
| Micromonospora | 6.56 | 10.63 | 9.42 | 2.20 | 1.45 |
| Formivibrio | 6.20 | 0.00 | 1.51 | 0.36 | 0.00 |
| Novosphingobium | 6.18 | 11.86 | 14.97 | 12.95 | 4.36 |
| Brevundimonas | 5.81 | 4.21 | 13.47 | 69.14 | 17.82 |
| Ilumatobacter | 5.80 | 5.05 | 2.61 | 3.27 | 1.09 |
| Oceanibaculum | 5.43 | 1.94 | 0.00 | 0.73 | 0.00 |
| Tumebacillus | 5.43 | 7.81 | 1.50 | 1.83 | 0.00 |
| Comamonas | 5.43 | 0.78 | 38.77 | 22.64 | 1.44 |
| Cryptomonadaceae | 5.41 | 0.00 | 0.00 | 0.00 | 0.00 |
| Paenibacillus | 5.41 | 7.15 | 2.64 | 2.20 | 7.62 |
| Aquimonas | 5.06 | 1.16 | 3.68 | 5.75 | 0.00 |
| Virgisporangium | 5.06 | 0.41 | 2.27 | 0.00 | 0.36 |
| Pedomicrobium | 5.03 | 11.78 | 4.11 | 1.86 | 0.73 |
| Sterolibacterium | 5.03 | 0.38 | 5.62 | 1.84 | 2.19 |
| Gp9 | 5.03 | 0.00 | 0.00 | 0.00 | 0.00 |
| Streptomyces | 5.02 | 14.23 | 2.65 | 1.14 | 3.27 |
| Paucimonas | 4.65 | 3.52 | 1.14 | 0.00 | 0.00 |
| Methylocystis | 4.65 | 13.21 | 9.37 | 16.35 | 4.00 |
| Pelobacter | 4.65 | 0.80 | 6.39 | 0.78 | 0.37 |
| Magnetospirillum | 4.64 | 4.28 | 13.53 | 19.50 | 91.94 |
| Jiangella | 4.63 | 0.00 | 0.37 | 0.00 | 0.00 |
| Chlorophyta | 4.28 | 2.68 | 1.11 | 1.08 | 73.10 |
| Naxibacter | 4.28 | 4.80 | 9.36 | 1.12 | 7.61 |
| Duganella | 4.27 | 6.62 | 5.62 | 2.19 | 1.09 |
| Verrucomicrobium | 4.26 | 4.77 | 2.25 | 8.30 | 0.36 |
| Herbaspirillum | 4.26 | 3.09 | 10.84 | 15.25 | 35.92 |
| Balneimonas | 4.26 | 2.36 | 1.50 | 4.83 | 0.73 |
| Methylocaldum | 4.26 | 2.72 | 2.64 | 5.13 | 0.00 |
| GpXIII | 4.25 | 4.64 | 237.05 | 14.95 | 5.11 |
| Chitiniphilus | 4.25 | 0.00 | 0.00 | 0.00 | 0.00 |
| Fervidicella | 4.25 | 10.09 | 41.72 | 10.28 | 2.18 |
| Gp15 | 4.25 | 3.10 | 0.00 | 0.00 | 0.00 |
| Prosthecobacter | 3.90 | 10.19 | 12.36 | 4.06 | 17.00 |
| Cystobacter | 3.88 | 6.65 | 8.28 | 2.17 | 4.36 |
| Rhodobacter | 3.88 | 6.57 | 53.51 | 15.35 | 5.46 |
| Agaricicola | 3.87 | 2.73 | 3.76 | 0.72 | 0.00 |
| Acidothermus | 3.87 | 5.57 | 10.90 | 1.08 | 0.36 |
| Devosia | 3.87 | 23.27 | 29.98 | 32.18 | 109.85 |
| Acetivibrio | 3.86 | 2.37 | 2.27 | 8.68 | 22.11 |
| Herminiimonas | 3.86 | 7.92 | 9.05 | 2.98 | 49.59 |
| Ralstonia | 3.86 | 2.04 | 3.41 | 0.36 | 1.45 |
| Gp2 | 3.51 | 1.55 | 0.00 | 0.36 | 0.00 |
| Nannocystis | 3.50 | 4.30 | 3.77 | 8.54 | 0.00 |
| Haliea | 3.50 | 1.92 | 14.24 | 3.67 | 3.62 |
| Craurococcus | 3.49 | 3.54 | 0.00 | 1.11 | 0.00 |
| Thiolamprovum | 3.48 | 0.00 | 0.38 | 0.00 | 0.00 |
| Saccharopolyspora | 3.48 | 0.41 | 0.00 | 0.00 | 0.00 |
| Oxalobacter | 3.13 | 7.78 | 17.30 | 6.93 | 5.81 |
| Sandarakinorhabdus | 3.12 | 0.80 | 0.00 | 0.00 | 0.00 |
| Piscinibacter | 3.11 | 5.06 | 6.75 | 4.69 | 0.72 |
| Enhygromyxa | 3.11 | 2.72 | 4.86 | 4.46 | 0.00 |
| Nitrosospira | 3.11 | 10.66 | 27.51 | 12.69 | 31.59 |
| Corallococcus | 3.10 | 2.45 | 4.52 | 1.11 | 0.36 |
| Roseibaca | 3.10 | 10.11 | 6.42 | 6.20 | 0.73 |
| Bauldia | 3.10 | 3.08 | 5.23 | 3.42 | 6.92 |
| Desulfobulbus | 3.09 | 0.76 | 0.00 | 0.00 | 0.36 |
| Sphingobium | 3.09 | 6.25 | 6.70 | 2.92 | 2.91 |
| Streptacidiphilus | 3.09 | 6.90 | 0.75 | 0.00 | 0.00 |
| Flavihumibacter | 3.08 | 2.34 | 7.89 | 14.77 | 1.44 |
| Limnohabitans | 3.08 | 1.59 | 6.02 | 14.79 | 21.75 |
| Syntrophus | 2.72 | 0.00 | 0.75 | 0.75 | 0.00 |
| Variovorax | 2.72 | 6.68 | 10.48 | 33.25 | 17.38 |
| Iamia | 2.71 | 2.73 | 3.41 | 2.22 | 0.37 |
| Syntrophorhabdus | 2.71 | 0.41 | 14.66 | 5.98 | 0.36 |
| Lacibacter | 2.71 | 1.94 | 30.69 | 3.65 | 50.78 |
| Defluviicoccus | 2.70 | 0.80 | 19.15 | 4.87 | 0.00 |
| Dongia | 2.70 | 5.58 | 6.36 | 2.91 | 2.17 |
| GpVIII | 2.70 | 0.41 | 0.76 | 0.36 | 0.00 |
| Caldimonas | 2.70 | 1.20 | 1.88 | 0.72 | 17.05 |
| Desulfovibrio | 2.70 | 2.70 | 13.89 | 3.72 | 0.00 |
| Emticicia | 2.35 | 6.89 | 8.13 | 4.39 | 25.95 |
| Gp11 | 2.33 | 0.77 | 0.00 | 0.00 | 0.00 |
| Methylobacterium | 2.33 | 5.11 | 0.00 | 0.00 | 1.45 |
| Rubellimicrobium | 2.33 | 0.00 | 0.75 | 0.00 | 5.45 |
| Myxococcus | 2.32 | 1.99 | 10.08 | 2.23 | 0.36 |
| Alkaliflexus | 2.32 | 9.22 | 62.65 | 71.03 | 2.90 |
| Desulfovirga | 2.32 | 0.76 | 10.87 | 1.11 | 10.90 |
| Agromyces | 2.32 | 0.39 | 3.78 | 1.12 | 0.00 |
| Sporacetigenium | 2.32 | 3.51 | 12.01 | 21.37 | 0.36 |
| Planococcus | 2.31 | 7.43 | 18.78 | 19.76 | 4.34 |
| Azotobacter | 2.31 | 0.39 | 0.38 | 0.36 | 0.00 |
| Aquaspirillum | 1.95 | 3.15 | 0.75 | 2.55 | 10.11 |
| Cytophaga | 1.95 | 0.41 | 1.85 | 0.36 | 0.00 |
| Desulfatirhabdium | 1.94 | 0.00 | 0.75 | 1.14 | 0.00 |
| Lysinibacillus | 1.94 | 6.75 | 1.89 | 2.93 | 7.26 |
| Plesiocystis | 1.94 | 3.55 | 4.10 | 3.72 | 1.09 |
| Methylococcus | 1.94 | 0.00 | 0.76 | 0.00 | 0.72 |
| OP11_genera_incertae_sedis | 1.93 | 0.00 | 1.11 | 0.00 | 0.00 |
| Enterobacter | 1.93 | 3.45 | 3.76 | 24.83 | 2.54 |
| Afipia | 1.93 | 8.68 | 3.03 | 5.95 | 2.53 |
| Hyalangium | 1.93 | 3.15 | 4.51 | 4.46 | 1.09 |
| Legionella | 1.93 | 2.00 | 0.38 | 0.36 | 0.00 |
| Pedobacter | 1.93 | 2.36 | 7.20 | 1.83 | 18.54 |
| Nitrobacter | 1.93 | 1.63 | 0.38 | 1.17 | 1.82 |
| Solitalea | 1.93 | 6.31 | 6.78 | 0.00 | 2.18 |
| Afifella | 1.93 | 0.00 | 0.00 | 0.00 | 0.00 |
| Alicycliphilus | 1.93 | 9.79 | 0.00 | 0.00 | 0.00 |
| Lewinella | 1.93 | 0.76 | 0.00 | 0.00 | 0.00 |
| GpV | 1.93 | 2.31 | 4.14 | 2.55 | 0.36 |
| Chitinophaga | 1.93 | 9.20 | 35.28 | 8.41 | 2.54 |
| Desulfocapsa | 1.93 | 10.03 | 18.69 | 12.87 | 6.88 |
| Leptothrix | 1.56 | 1.15 | 2.98 | 30.14 | 18.36 |
| Gp12 | 1.56 | 1.95 | 0.00 | 0.00 | 0.00 |
| Smithella | 1.56 | 3.13 | 10.48 | 2.20 | 0.00 |
| Anaerovorax | 1.55 | 5.04 | 26.61 | 49.94 | 87.83 |
| Fluviicola | 1.55 | 0.78 | 3.75 | 10.04 | 2.90 |
| Peredibacter | 1.55 | 1.53 | 6.00 | 2.17 | 0.36 |
| Janthinobacterium | 1.55 | 1.14 | 13.15 | 29.87 | 9.45 |
| Methylohalomonas | 1.55 | 0.39 | 0.38 | 0.00 | 0.00 |
| Thiobacter | 1.55 | 0.39 | 0.37 | 0.36 | 0.00 |
| Stella | 1.55 | 8.96 | 1.52 | 2.93 | 14.18 |
| Turneriella | 1.55 | 0.00 | 31.22 | 9.40 | 23.83 |
| Alterococcus | 1.55 | 1.16 | 0.38 | 1.86 | 0.00 |
| Azohydromonas | 1.55 | 2.02 | 1.11 | 0.36 | 1.45 |
| Clostridium XlVa | 1.55 | 0.38 | 1.88 | 8.96 | 1.09 |
| Haloferula | 1.55 | 4.73 | 0.00 | 0.00 | 0.00 |
| Lentzea | 1.55 | 2.41 | 0.00 | 0.36 | 0.00 |
| Sandaracinobacter | 1.55 | 1.17 | 4.13 | 0.00 | 31.66 |
| Sphaerobacter | 1.55 | 3.55 | 0.37 | 0.00 | 0.37 |
| Vasilyevaea | 1.55 | 0.77 | 0.37 | 0.36 | 1.45 |
| Brevibacillus | 1.55 | 0.82 | 0.38 | 1.87 | 0.00 |
| Hydrotalea | 1.55 | 1.96 | 5.60 | 4.50 | 5.07 |
| Methylobacter | 1.55 | 0.79 | 66.28 | 30.89 | 9.05 |
| Pseudonocardia | 1.55 | 3.59 | 0.75 | 0.00 | 1.81 |
| Sporomusa | 1.55 | 1.17 | 2.23 | 4.05 | 0.00 |
| Undibacterium | 1.55 | 1.18 | 0.76 | 1.86 | 42.80 |
| Vulcanibacillus | 1.55 | 0.00 | 0.00 | 0.00 | 0.00 |
| Ponticaulis | 1.55 | 0.79 | 12.66 | 4.02 | 17.76 |
| Hydrogenophaga | 1.54 | 1.58 | 7.87 | 31.44 | 91.35 |
| Denitratisoma | 1.54 | 1.15 | 8.29 | 39.70 | 6.85 |
| Rubrobacter | 1.54 | 0.82 | 0.00 | 0.00 | 0.00 |
| Rhodomicrobium | 1.54 | 0.82 | 2.27 | 6.37 | 0.36 |
| Thermodesulforhabdus | 1.54 | 0.00 | 0.00 | 0.00 | 0.00 |
| Sporobacter | 1.17 | 0.77 | 8.29 | 8.16 | 5.08 |
| Blastochloris | 1.17 | 7.47 | 1.12 | 1.50 | 0.00 |
| Methylosarcina | 1.17 | 0.38 | 4.51 | 10.11 | 0.00 |
| Saccharofermentans | 1.17 | 0.41 | 2.65 | 2.65 | 1.82 |
| Desertibacter | 1.17 | 3.54 | 1.89 | 1.48 | 0.37 |
| Janibacter | 1.17 | 12.70 | 4.49 | 6.20 | 20.31 |
| Luteibacter | 1.17 | 1.96 | 0.00 | 3.62 | 0.00 |
| Beijerinckia | 1.16 | 7.46 | 12.07 | 8.95 | 31.58 |
| Mesorhizobium | 1.16 | 4.33 | 1.12 | 10.94 | 5.08 |
| Sedimentibacter | 1.16 | 0.79 | 4.89 | 11.67 | 1.82 |
| Rhodococcus | 1.16 | 5.12 | 1.50 | 0.00 | 1.81 |
| Solimonas | 1.16 | 0.38 | 0.00 | 0.00 | 0.00 |
| Verrucosispora | 1.16 | 4.84 | 2.99 | 0.39 | 0.00 |
| Amorphus | 1.16 | 1.95 | 1.88 | 0.73 | 0.00 |
| Actinoplanes | 1.16 | 11.29 | 3.40 | 0.00 | 0.73 |
| Anaerobacter | 1.16 | 1.17 | 4.88 | 10.55 | 46.07 |
| Cellulosilyticum | 1.16 | 0.00 | 0.38 | 1.09 | 0.36 |
| Dehalogenimonas | 1.16 | 2.36 | 3.38 | 2.62 | 0.00 |
| Cupriavidus | 1.16 | 4.71 | 0.00 | 0.36 | 0.00 |
| Salisaeta | 1.16 | 0.77 | 0.75 | 61.68 | 0.00 |
| Sporosarcina | 1.16 | 10.83 | 4.13 | 15.21 | 26.87 |
| Stigmatella | 1.16 | 0.41 | 1.51 | 0.00 | 1.09 |
| Blastomonas | 1.16 | 1.15 | 0.76 | 0.00 | 0.00 |
| Desulfacinum | 1.16 | 2.38 | 1.13 | 0.00 | 0.00 |
| Thioalkalispira | 1.16 | 0.00 | 3.70 | 1.11 | 0.36 |
| Caulobacter | 0.78 | 1.20 | 5.25 | 11.54 | 48.21 |
| Erythromicrobium | 0.78 | 0.76 | 0.00 | 0.00 | 0.00 |
| Ferrimicrobium | 0.78 | 0.38 | 2.62 | 1.81 | 10.50 |
| Niabella | 0.78 | 2.70 | 10.14 | 4.33 | 2.18 |
| Rhodocista | 0.78 | 1.56 | 2.27 | 3.40 | 1.45 |
| Roseateles | 0.78 | 1.98 | 0.74 | 2.62 | 2.53 |
| TM7_genera_incertae_sedis | 0.78 | 0.00 | 1.49 | 0.00 | 0.00 |
| Bryobacter | 0.78 | 0.00 | 0.00 | 0.00 | 0.72 |
| Fulvivirga | 0.78 | 0.00 | 0.76 | 0.72 | 0.36 |
| Gp23 | 0.78 | 0.00 | 2.63 | 0.00 | 0.00 |
| Mycoplana | 0.78 | 1.96 | 0.00 | 0.00 | 0.00 |
| Orientia | 0.78 | 0.00 | 2.25 | 5.14 | 2.53 |
| Rubribacterium | 0.78 | 0.00 | 0.00 | 0.00 | 0.00 |
| Shinella | 0.78 | 1.56 | 1.11 | 3.26 | 19.95 |
| Thermogymnomonas | 0.78 | 0.00 | 9.02 | 9.19 | 1.09 |
| Amaricoccus | 0.78 | 0.00 | 0.00 | 0.00 | 0.00 |
| Archangium | 0.78 | 0.00 | 0.00 | 0.00 | 0.00 |
| Fulvimonas | 0.78 | 1.95 | 0.00 | 0.00 | 0.00 |
| Geosporobacter | 0.78 | 0.00 | 6.38 | 0.00 | 0.00 |
| Proteiniclasticum | 0.78 | 2.35 | 7.89 | 6.39 | 9.78 |
| Pullulanibacillus | 0.78 | 0.00 | 0.00 | 0.00 | 0.00 |
| Saccharibacillus | 0.78 | 1.18 | 0.00 | 0.00 | 0.00 |
| Stappia | 0.78 | 0.00 | 0.00 | 0.00 | 0.00 |
| Thermoflavimicrobium | 0.78 | 0.00 | 2.63 | 1.50 | 0.00 |
| Angustibacter | 0.77 | 3.95 | 0.00 | 0.00 | 0.36 |
| Catellatospora | 0.77 | 0.38 | 0.76 | 0.00 | 0.00 |
| Dactylosporangium | 0.77 | 1.59 | 0.38 | 0.36 | 1.08 |
| Ferrithrix | 0.77 | 2.35 | 0.38 | 0.00 | 0.00 |
| OD1_genera_incertae_sedis | 0.77 | 1.56 | 9.74 | 34.33 | 2.53 |
| Thiohalomonas | 0.77 | 0.00 | 0.00 | 0.00 | 0.00 |
| Bacteriovorax | 0.77 | 5.00 | 0.76 | 3.39 | 0.00 |
| Blastococcus | 0.77 | 16.41 | 2.64 | 0.75 | 2.92 |
| Centipeda | 0.77 | 1.59 | 4.11 | 3.76 | 24.29 |
| Flavitalea | 0.77 | 9.46 | 1.52 | 2.56 | 35.98 |
| Inquilinus | 0.77 | 1.20 | 5.62 | 1.11 | 1.09 |
| Methanosarcina | 0.77 | 0.00 | 1.12 | 1.47 | 0.73 |
| Nitrosomonas | 0.77 | 0.00 | 0.00 | 0.36 | 0.00 |
| Nonomuraea | 0.77 | 0.39 | 0.00 | 0.00 | 1.08 |
| Parasegetibacter | 0.77 | 2.39 | 40.14 | 91.91 | 137.72 |
| Sphaerisporangium | 0.77 | 0.00 | 0.00 | 0.00 | 0.00 |
| Sphingopyxis | 0.77 | 2.38 | 3.76 | 5.05 | 6.90 |
| Thermobrachium | 0.77 | 3.11 | 36.09 | 16.66 | 2.18 |
| Desulfarculus | 0.77 | 0.00 | 0.00 | 0.00 | 0.00 |
| Filomicrobium | 0.77 | 0.38 | 1.12 | 0.75 | 0.72 |
| Geothermobacter | 0.77 | 4.32 | 0.00 | 0.00 | 0.00 |
| Luteolibacter | 0.77 | 1.91 | 3.75 | 6.74 | 1.83 |
| Methylocapsa | 0.77 | 0.00 | 2.25 | 3.68 | 5.08 |
| Pseudoxanthomonas | 0.77 | 1.96 | 0.00 | 13.15 | 4.00 |
| Salinibacter | 0.77 | 0.00 | 0.00 | 0.00 | 0.00 |
| Salinispora | 0.77 | 1.57 | 0.74 | 0.00 | 0.00 |
| Sorangium | 0.77 | 3.12 | 5.26 | 3.32 | 4.71 |
| Acidisoma | 0.39 | 3.13 | 0.38 | 0.75 | 0.37 |
| Arthrobacter | 0.39 | 53.21 | 2.64 | 3.67 | 20.72 |
| Chelatococcus | 0.39 | 0.78 | 0.00 | 0.73 | 0.37 |
| Curvibacter | 0.39 | 1.15 | 2.99 | 15.65 | 3.27 |
| Ensifer | 0.39 | 1.22 | 3.40 | 11.67 | 11.95 |
| Hylemonella | 0.39 | 0.00 | 0.38 | 0.00 | 2.17 |
| Luedemannella | 0.39 | 0.39 | 6.35 | 1.08 | 1.08 |
| Pelotomaculum | 0.39 | 0.00 | 4.48 | 4.54 | 0.00 |
| Rhodovibrio | 0.39 | 0.78 | 0.00 | 0.00 | 0.00 |
| Thauera | 0.39 | 1.57 | 2.63 | 1.87 | 1.80 |
| Vogesella | 0.39 | 2.76 | 0.00 | 0.00 | 0.00 |
| Acidicaldus | 0.39 | 0.00 | 1.13 | 1.08 | 0.00 |
| Actinomadura | 0.39 | 3.92 | 1.90 | 2.97 | 0.00 |
| Actinomycetospora | 0.39 | 0.41 | 0.00 | 0.00 | 0.00 |
| Actinosynnema | 0.39 | 0.41 | 0.00 | 0.00 | 0.00 |
| Anoxybacillus | 0.39 | 4.06 | 0.00 | 0.00 | 4.72 |
| Aurantimonas | 0.39 | 0.00 | 0.00 | 0.00 | 0.36 |
| Clostridium XI | 0.39 | 0.39 | 3.02 | 4.82 | 1.81 |
| Delftia | 0.39 | 0.00 | 1.88 | 0.00 | 0.36 |
| Diaphorobacter | 0.39 | 0.79 | 2.63 | 1.14 | 1.10 |
| Flavobacterium | 0.39 | 1.15 | 2.28 | 2.24 | 1.82 |
| Kaistia | 0.39 | 0.00 | 0.00 | 0.00 | 0.36 |
| Nocardioides | 0.39 | 13.48 | 1.50 | 1.45 | 3.99 |
| Paenisporosarcina | 0.39 | 0.00 | 0.37 | 0.36 | 0.37 |
| Perlucidibaca | 0.39 | 0.76 | 14.36 | 8.86 | 5.81 |
| Porphyrobacter | 0.39 | 1.19 | 0.00 | 0.39 | 0.00 |
| Renibacterium | 0.39 | 5.62 | 0.37 | 0.00 | 0.37 |
| Roseiflexus | 0.39 | 1.93 | 0.00 | 0.00 | 0.00 |
| Rugosimonospora | 0.39 | 1.95 | 0.75 | 0.00 | 0.36 |
| Segetibacter | 0.39 | 9.64 | 0.00 | 0.00 | 19.58 |
| Selenomonas | 0.39 | 0.38 | 0.37 | 0.72 | 0.00 |
| Sinobacter | 0.39 | 0.79 | 0.00 | 0.00 | 0.00 |
| Subdivision5_genera_incertae_sedis | 0.39 | 0.39 | 3.00 | 2.20 | 0.00 |
| Acetitomaculum | 0.39 | 0.00 | 0.00 | 0.39 | 0.00 |
| Anaeroarcus | 0.39 | 0.77 | 2.61 | 1.14 | 0.00 |
| Armatimonas/Armatimonadetes_gp1 | 0.39 | 0.77 | 2.95 | 0.36 | 1.09 |
| Asanoa | 0.39 | 2.30 | 3.00 | 2.54 | 0.00 |
| Caedibacter | 0.39 | 0.00 | 0.00 | 0.39 | 0.00 |
| Catelliglobosispora | 0.39 | 1.15 | 4.13 | 0.36 | 0.00 |
| Cellvibrio | 0.39 | 1.20 | 0.38 | 0.00 | 0.36 |
| GpXI | 0.39 | 0.00 | 0.00 | 0.39 | 0.00 |
| Labrys | 0.39 | 1.59 | 0.00 | 0.00 | 0.00 |
| Lutispora | 0.39 | 0.38 | 0.76 | 0.36 | 1.09 |
| Methanocella | 0.39 | 0.38 | 4.14 | 4.12 | 0.72 |
| Microtetraspora | 0.39 | 1.18 | 0.00 | 0.00 | 0.00 |
| Papillibacter | 0.39 | 0.00 | 1.85 | 3.41 | 2.16 |
| Parachlamydia | 0.39 | 1.17 | 0.00 | 0.00 | 0.00 |
| Propionispira | 0.39 | 0.00 | 0.00 | 6.83 | 0.00 |
| Rickettsia | 0.39 | 0.00 | 0.00 | 0.36 | 0.00 |
| Roseomonas | 0.39 | 3.13 | 4.10 | 10.23 | 13.79 |
| Simplicispira | 0.39 | 2.75 | 1.13 | 28.87 | 7.68 |
| Syntrophomonas | 0.39 | 0.41 | 6.79 | 8.30 | 4.34 |
| Tepidibacter | 0.39 | 0.41 | 0.00 | 0.00 | 0.00 |
| Acetanaerobacterium | 0.00 | 0.00 | 0.38 | 0.00 | 0.00 |
| Acetonema | 0.00 | 0.00 | 0.00 | 0.39 | 0.00 |
| Achromobacter | 0.00 | 1.99 | 0.38 | 0.36 | 0.00 |
| Acidisphaera | 0.00 | 0.00 | 0.38 | 1.17 | 4.36 |
| Acidobacterium | 0.00 | 0.80 | 0.00 | 0.00 | 0.00 |
| Acinetobacter | 0.00 | 0.82 | 0.38 | 1.08 | 0.00 |
| Actinoallomurus | 0.00 | 1.18 | 0.00 | 0.00 | 0.73 |
| Actinoalloteichus | 0.00 | 0.41 | 0.38 | 0.00 | 0.00 |
| Aeromicrobium | 0.00 | 8.76 | 0.00 | 0.00 | 0.00 |
| Albidiferax | 0.00 | 0.00 | 0.00 | 52.93 | 4.68 |
| Alloiococcus | 0.00 | 0.00 | 1.11 | 1.11 | 0.00 |
| Aminobacter | 0.00 | 0.00 | 1.12 | 2.95 | 5.81 |
| Ammoniphilus | 0.00 | 0.00 | 0.38 | 1.12 | 0.00 |
| Amnibacterium | 0.00 | 0.39 | 0.75 | 0.00 | 0.00 |
| Amycolatopsis | 0.00 | 15.61 | 0.00 | 0.00 | 0.00 |
| Anaerofustis | 0.00 | 0.00 | 1.89 | 0.78 | 0.00 |
| Anaerophaga | 0.00 | 0.82 | 6.71 | 12.31 | 0.00 |
| Anaerosporobacter | 0.00 | 0.00 | 0.37 | 1.82 | 0.36 |
| Ancylobacter | 0.00 | 1.20 | 0.00 | 0.00 | 0.00 |
| Aneurinibacillus | 0.00 | 0.39 | 0.00 | 0.00 | 0.00 |
| Aquincola | 0.00 | 0.41 | 2.63 | 1.14 | 0.73 |
| Aquisalibacillus | 0.00 | 0.00 | 1.89 | 0.00 | 0.00 |
| Aspromonas | 0.00 | 5.14 | 0.00 | 0.00 | 1.82 |
| Asticcacaulis | 0.00 | 1.58 | 0.00 | 0.72 | 0.00 |
| Azospirillum | 0.00 | 1.96 | 8.66 | 24.30 | 64.98 |
| Bangiophyceae | 0.00 | 2.02 | 0.00 | 0.00 | 0.36 |
| Beggiatoa | 0.00 | 0.76 | 0.00 | 0.00 | 0.00 |
| Belnapia | 0.00 | 0.00 | 0.75 | 0.75 | 5.08 |
| Bosea | 0.00 | 1.17 | 3.77 | 7.00 | 6.54 |
| Burkholderia | 0.00 | 24.84 | 1.11 | 1.11 | 0.73 |
| Caloramator | 0.00 | 0.00 | 1.13 | 0.73 | 6.52 |
| Castellaniella | 0.00 | 0.78 | 0.00 | 0.00 | 0.00 |
| Catenulispora | 0.00 | 0.80 | 0.00 | 0.00 | 0.00 |
| Cellulomonas | 0.00 | 1.56 | 2.25 | 10.09 | 0.00 |
| Cesiribacter | 0.00 | 1.55 | 0.75 | 0.72 | 0.00 |
| Chloroflexus | 0.00 | 0.00 | 1.10 | 2.56 | 0.00 |
| Chryseobacterium | 0.00 | 0.78 | 0.00 | 0.39 | 0.00 |
| Clostridium IV | 0.00 | 0.00 | 0.00 | 0.00 | 0.73 |
| Clostridium XlVb | 0.00 | 0.00 | 0.00 | 0.00 | 0.36 |
| Cohnella | 0.00 | 0.00 | 0.37 | 0.36 | 0.73 |
| Collimonas | 0.00 | 0.80 | 0.00 | 0.00 | 2.18 |
| Croceicoccus | 0.00 | 0.00 | 0.00 | 4.41 | 25.37 |
| Crossiella | 0.00 | 1.19 | 0.38 | 0.00 | 0.00 |
| Cryptanaerobacter | 0.00 | 0.00 | 0.00 | 0.75 | 1.10 |
| Curtobacterium | 0.00 | 0.82 | 0.00 | 0.00 | 0.00 |
| Dehalobacter | 0.00 | 0.00 | 0.75 | 1.50 | 8.69 |
| Deinobacterium | 0.00 | 0.00 | 0.00 | 0.00 | 5.09 |
| Dendrosporobacter | 0.00 | 0.77 | 9.40 | 13.84 | 16.31 |
| Desulfitibacter | 0.00 | 0.78 | 0.00 | 0.00 | 0.00 |
| Desulfitobacterium | 0.00 | 0.00 | 4.51 | 2.24 | 5.46 |
| Desulfobacca | 0.00 | 0.00 | 0.75 | 1.48 | 0.00 |
| Desulfopila | 0.00 | 0.39 | 0.00 | 0.36 | 0.00 |
| Desulforegula | 0.00 | 0.00 | 0.00 | 0.72 | 0.36 |
| Desulfosporosinus | 0.00 | 0.79 | 1.87 | 2.59 | 5.45 |
| Desulfurispora | 0.00 | 0.00 | 0.00 | 0.00 | 0.00 |
| Dethiobacter | 0.00 | 0.00 | 0.00 | 0.00 | 0.73 |
| Dokdonella | 0.00 | 5.64 | 0.00 | 0.73 | 0.00 |
| Dyadobacter | 0.00 | 0.77 | 1.13 | 16.09 | 1.09 |
| Dyella | 0.00 | 5.97 | 0.00 | 0.00 | 0.36 |
| Empedobacter | 0.00 | 0.00 | 0.76 | 0.75 | 0.36 |
| Escherichia/Shigella | 0.00 | 0.00 | 0.00 | 0.78 | 0.00 |
| Ethanoligenens | 0.00 | 0.00 | 0.00 | 0.00 | 1.09 |
| Falsibacillus | 0.00 | 1.61 | 0.00 | 0.00 | 0.37 |
| Ferribacterium | 0.00 | 0.00 | 0.38 | 0.00 | 0.72 |
| Flavonifractor | 0.00 | 0.00 | 0.76 | 0.00 | 0.00 |
| Fodinicurvata | 0.00 | 0.00 | 1.89 | 1.11 | 0.73 |
| Fontibacillus | 0.00 | 0.79 | 0.00 | 0.00 | 0.00 |
| Frateuria | 0.00 | 1.63 | 0.00 | 0.00 | 0.00 |
| Geodermatophilus | 0.00 | 3.21 | 0.00 | 0.00 | 0.37 |
| Geothrix | 0.00 | 0.38 | 1.49 | 5.81 | 0.00 |
| Gordonia | 0.00 | 0.82 | 0.00 | 0.00 | 0.00 |
| GpIX | 0.00 | 0.00 | 0.75 | 0.73 | 0.00 |
| Granulicella | 0.00 | 1.61 | 0.00 | 0.00 | 0.00 |
| Heliothrix | 0.00 | 0.00 | 1.85 | 3.69 | 0.00 |
| Herbiconiux | 0.00 | 0.00 | 0.00 | 0.72 | 0.37 |
| Herpetosiphon | 0.00 | 1.59 | 0.00 | 0.00 | 0.00 |
| Hirschia | 0.00 | 0.00 | 0.37 | 0.00 | 1.09 |
| Hymenobacter | 0.00 | 1.98 | 0.00 | 0.73 | 0.36 |
| Kineosporia | 0.00 | 0.82 | 0.00 | 0.00 | 0.00 |
| Knoellia | 0.00 | 0.41 | 0.76 | 0.00 | 0.00 |
| Kribbella | 0.00 | 3.63 | 0.00 | 0.00 | 0.00 |
| Kutzneria | 0.00 | 1.61 | 0.00 | 0.00 | 0.00 |
| Lampropedia | 0.00 | 0.00 | 0.00 | 2.16 | 0.00 |
| Leifsonia | 0.00 | 6.37 | 1.11 | 1.48 | 3.28 |
| Meniscus | 0.00 | 0.00 | 0.00 | 0.00 | 1.45 |
| Methanoregula | 0.00 | 0.00 | 0.37 | 0.78 | 0.00 |
| Methylomicrobium | 0.00 | 0.78 | 0.38 | 0.00 | 0.00 |
| Methylomonas | 0.00 | 0.00 | 9.33 | 13.43 | 5.81 |
| Methylophilus | 0.00 | 1.63 | 0.00 | 3.39 | 0.00 |
| Methylovorus | 0.00 | 0.78 | 0.00 | 0.00 | 0.00 |
| Mitsuokella | 0.00 | 0.39 | 0.00 | 0.36 | 0.72 |
| Modestobacter | 0.00 | 3.98 | 0.37 | 0.36 | 0.00 |
| Mucilaginibacter | 0.00 | 1.20 | 0.75 | 1.08 | 0.00 |
| Nevskia | 0.00 | 1.59 | 0.00 | 0.00 | 0.00 |
| Nocardia | 0.00 | 1.20 | 0.00 | 0.00 | 0.00 |
| Oceanicaulis | 0.00 | 0.76 | 2.98 | 3.35 | 0.00 |
| Oligotropha | 0.00 | 0.39 | 0.38 | 0.72 | 1.09 |
| Orbus | 0.00 | 0.82 | 0.00 | 0.00 | 0.00 |
| Ornithinibacillus | 0.00 | 0.39 | 0.00 | 0.00 | 0.37 |
| Oryzihumus | 0.00 | 1.99 | 0.00 | 0.36 | 0.37 |
| Oscillibacter | 0.00 | 0.00 | 1.12 | 1.53 | 2.53 |
| Oxalophagus | 0.00 | 0.41 | 0.00 | 0.00 | 0.00 |
| Oxobacter | 0.00 | 3.49 | 21.00 | 28.76 | 82.18 |
| Pandoraea | 0.00 | 0.41 | 0.00 | 0.36 | 0.00 |
| Paracoccus | 0.00 | 0.00 | 0.76 | 0.72 | 0.00 |
| Parasporobacterium | 0.00 | 0.38 | 1.13 | 1.50 | 5.43 |
| Parvularcula | 0.00 | 1.59 | 0.00 | 0.00 | 0.37 |
| Pelomonas | 0.00 | 0.00 | 0.00 | 1.81 | 2.53 |
| Pelosinus | 0.00 | 0.00 | 0.76 | 1.09 | 0.36 |
| Pelospora | 0.00 | 0.00 | 1.13 | 2.26 | 1.45 |
| Phyllobacterium | 0.00 | 0.78 | 0.00 | 0.00 | 0.00 |
| Pilimelia | 0.00 | 3.19 | 0.00 | 0.36 | 0.00 |
| Pimelobacter | 0.00 | 0.78 | 0.00 | 0.00 | 0.00 |
| Planococcaceae_incertae_sedis | 0.00 | 3.16 | 0.75 | 0.73 | 2.18 |
| Planomicrobium | 0.00 | 0.00 | 0.00 | 0.78 | 0.00 |
| Planomonospora | 0.00 | 0.00 | 0.00 | 0.78 | 0.00 |
| Pleomorphomonas | 0.00 | 0.38 | 0.00 | 3.66 | 0.00 |
| Polaromonas | 0.00 | 0.00 | 0.38 | 1.17 | 15.91 |
| Polymorphospora | 0.00 | 0.39 | 0.00 | 0.00 | 0.00 |
| Prolixibacter | 0.00 | 0.00 | 0.00 | 0.00 | 0.73 |
| Promicromonospora | 0.00 | 1.61 | 0.00 | 0.00 | 0.00 |
| Propionispora | 0.00 | 0.00 | 0.00 | 0.72 | 0.73 |
| Propionivibrio | 0.00 | 0.77 | 2.62 | 3.33 | 0.73 |
| Pseudomonas | 0.00 | 5.19 | 3.01 | 1.14 | 0.00 |
| Rhizobium | 0.00 | 6.28 | 31.12 | 62.47 | 71.11 |
| Rhodanobacter | 0.00 | 20.81 | 0.38 | 0.00 | 0.00 |
| Rhodobium | 0.00 | 3.11 | 0.37 | 0.00 | 0.00 |
| Rhodoblastus | 0.00 | 0.00 | 0.00 | 3.64 | 0.00 |
| Rhodocyclus | 0.00 | 0.00 | 0.38 | 0.36 | 0.00 |
| Rhodocytophaga | 0.00 | 0.00 | 0.38 | 0.00 | 5.43 |
| Rhodopila | 0.00 | 1.17 | 2.25 | 3.84 | 6.52 |
| Rhodopseudomonas | 0.00 | 0.39 | 0.37 | 4.50 | 2.17 |
| Roseococcus | 0.00 | 0.00 | 0.00 | 0.36 | 2.52 |
| Rubritepida | 0.00 | 0.00 | 0.37 | 0.75 | 0.00 |
| Rudaea | 0.00 | 3.19 | 0.75 | 0.00 | 0.00 |
| Rummeliibacillus | 0.00 | 0.41 | 0.38 | 0.00 | 0.00 |
| Saccharothrix | 0.00 | 1.20 | 0.00 | 0.00 | 0.00 |
| Salinibacterium | 0.00 | 3.65 | 0.00 | 0.00 | 1.09 |
| Sarcina | 0.00 | 0.76 | 0.76 | 0.00 | 0.73 |
| Saxeibacter | 0.00 | 0.82 | 0.38 | 0.00 | 0.36 |
| Schlesneria | 0.00 | 0.82 | 0.00 | 0.00 | 0.00 |
| Shimazuella | 0.00 | 0.38 | 0.37 | 0.00 | 0.00 |
| Smaragdicoccus | 0.00 | 0.00 | 0.00 | 0.00 | 0.73 |
| Sphaerotilus | 0.00 | 0.00 | 0.00 | 1.09 | 0.00 |
| Sphingobacterium | 0.00 | 3.21 | 0.00 | 0.00 | 0.00 |
| Spirochaeta | 0.00 | 0.00 | 1.86 | 0.00 | 0.00 |
| Sporichthya | 0.00 | 0.38 | 0.38 | 0.72 | 0.00 |
| Sporocytophaga | 0.00 | 0.00 | 1.13 | 0.00 | 1.46 |
| Sporotalea | 0.00 | 0.00 | 0.00 | 0.39 | 1.09 |
| Sporotomaculum | 0.00 | 0.00 | 1.51 | 0.00 | 2.54 |
| Stakelama | 0.00 | 0.80 | 0.00 | 0.00 | 0.00 |
| Stenotrophomonas | 0.00 | 2.35 | 1.89 | 1.46 | 0.00 |
| Streptosporangium | 0.00 | 1.61 | 0.00 | 0.00 | 0.37 |
| Succinispira | 0.00 | 1.17 | 0.38 | 0.75 | 0.36 |
| Symbiobacterium | 0.00 | 0.00 | 0.76 | 1.84 | 0.36 |
| Syntrophothermus | 0.00 | 0.00 | 0.76 | 1.14 | 0.72 |
| Telmatospirillum | 0.00 | 0.00 | 23.20 | 0.00 | 0.00 |
| Terrabacter | 0.00 | 4.33 | 0.00 | 0.36 | 1.45 |
| Thermoactinomyces | 0.00 | 0.00 | 0.37 | 0.00 | 0.36 |
| Thermomonas | 0.00 | 1.63 | 1.14 | 0.00 | 0.00 |
| Thiobacillus | 0.00 | 0.00 | 10.89 | 0.00 | 2.18 |
| Thioprofundum | 0.00 | 1.16 | 0.75 | 1.82 | 0.00 |
| Turicibacter | 0.00 | 0.00 | 0.37 | 0.36 | 0.00 |
| Umezawaea | 0.00 | 3.63 | 0.00 | 0.00 | 0.00 |
| Virgibacillus | 0.00 | 1.19 | 0.00 | 0.00 | 0.00 |
| Xanthobacter | 0.00 | 1.17 | 1.13 | 8.76 | 4.37 |

Table I Principle coordinate analysis (PCoA) of GeoChip and 16S sequencing data with environmental properties among five arsenic contaminated soils from different geographic locations.

| Environmental variable | | R/*P* value | |
| --- | --- | --- | --- |
|  |  | GeoChip 4.0 | 16S OTUs |
| Soil physicochemical parameters | pH | **0.506/0.016** | **0.823/0.001** |
|  | SOC (%) | 0.272/0.146 | **0.694/0.005** |
|  | Available phosphorus (mg/kg) | **0.511/0.010** | **0.519/0.004** |
|  | Amorphous Fe (mg/kg) | 0.264/0.178 | **0.899/0.001** |
|  | Total As (μg/kg) | 0.146/0.392 | **0.945/0.001** |
|  | Phosphate extractable As (μg/kg) | 0.101/0.531 | **0.961/0.001** |

Table J Mantel tests of GeoChip and 16S sequencing data with environmental properties among five arsenic contaminated soils from different geographic locations.

| Environmental variable | | R/*P* value | |
| --- | --- | --- | --- |
|  |  | GeoChip 4.0 | 16S OTUs |
| Soil physicochemical parameters | pH | **0.367/0.006** | **0.320/0.039** |
|  | SOC (%) | 0.072/0.196 | 0.173/0.177 |
|  | Available phosphorus (mg/kg) | 0.101/0.148 | -0.159/0.800 |
|  | Amorphous Fe (mg/kg) | **0.240/0.022** | 0.132/0.177 |
|  | Total As (μg/kg) | 0.102/0.122 | 0.145/0.103 |
|  | Phosphate extractable As (μg/kg) | **0.333/0.008** | **0.476/0.001** |
